# Ergosteroid and Phenolic Constituents from the Mushroom *Sanghuangporus vaninii* with Anti-Inflammatory Activity

**DOI:** 10.3390/ijms27073315

**Published:** 2026-04-07

**Authors:** Yu-Xin Gao, Yue-Tong Zhu, Almutamad Sheikho, Qiu-Yu Zhao, Ya-Ru Wang, Yong-Hua Wang, Yu-Qi Gao, Jin-Ming Gao

**Affiliations:** 1 College of Life Sciences, Northwest University, Xi’an 710069, China; gyxshane2008@163.com (Y.-X.G.); zhaoqiuyu95@163.com (Q.-Y.Z.); wangayru1101@163.com (Y.-R.W.); 2 Shaanxi Key Laboratory of Natural Products & Chemical Biology, College of Chemistry & Pharmacy, Northwest A&F University, Yangling 712100, China; 15037143303@163.com (Y.-T.Z.); almutamadsheikho@nwafu.edu.cn (A.S.); jinminggao@nwsuaf.edu.cn (J.-M.G.); 3 College of Food Science and Technology, Northwest University, Xi’an 710069, China

**Keywords:** *Sanghuangporus vaninii*, ergosteroids, ECD calculation, anti-inflammatory, molecular docking

## Abstract

*Sanghuangporus vaninii* (Ljub.) is an edible and medicinal macrofungus that has become the main strain for artificial cultivation of Sanghuang. In this study, twenty-six compounds (**1**–**26**), including five previously undescribed ergosterols, named sanghusterols A–E (**1**–**5**), were isolated from the fruiting bodies of *S. vaninii*. Their structures were elucidated by spectroscopic methods and electronic circular dichroism (ECD) calculations. Compounds **1**, **15**, **17**, **21** and **25** exhibited potent inhibitory activity against NO production with the IC_50_ value of 8.3–14.8 μM and dose-dependently decreased iNOS and COX-2 protein expression in RAW264.7 cells. Molecular docking studies confirmed the capacity of compounds **1**, **15**, **17**, **21** and **25** to interact with iNOS and COX-2 proteins. These findings may provide a solid phytochemical and pharmacological basis for developing the mushroom as potential anti-inflammatory agents.

## 1. Introduction

Chronic inflammation is a key driver in the initiation, progression, invasion, and metastasis of tumors. In order to clear tumor cells, the immune system is activated and macrophages are stimulated, leading to the production of pro-inflammatory mediators including cytokines such as interleukin (IL)-1*β* and tumor necrosis factor (TNF)-*α* [1]. The genus *Sanghuangporus* comprises edible and medicinal fungi that have been used in China for over 2000 years to treat various diseases [2]. They contain many chemical components with various pharmacological effects, including anti-tumor, antioxidant, hypoglycemic, and anti-inflammatory properties, with their anti-tumor activity being particularly notable, and they are therefore known as ‘forest gold’ [3]. *Sanghuangporus vaninii* (Ljub.) can be artificially cultivated in the bag and cut log, the fruiting bodies of which are rich in sterols, polysaccharides, flavonoids, polyphenols, etc. [4]. Among sterol compounds, ergosterol constitutes the largest proportion. Through structural modifications such as ring cleavage, rearrangement, and degradation, ergosterol can form novel carbon skeletons and exhibit diverse biological activities, particularly its anti-inflammatory activity, which even surpasses that of certain pharmaceutical drugs [5,6].

To elucidate the anti-tumor active components of *S. vaninii* while continuing our ongoing research on extracting novel bioactive substances from large fungi [7,8,9,10,11], a chemical investigation on cultivated *S. vaninii* was carried out, leading to the isolation of eighteen steroids (**1**–**18**), including five new ones (**1**–**5**), along with other eight known compounds (**19**–**26**) (Figure 1). The inhibitory effects of compounds **1**–**13**, **15**–**21**, **25** and **26** on anti-inflammatory activity were evaluated, and compounds **1**, **15**, **17**, **21** and **25** significantly inhibited the release of NO in LPS-stimulated RAW264.7 cells, with IC_50_ values ranging from 8.3 to 14.8 μM. Moreover, they blocked the expression of pro-inflammatory cytokines (iNOS and COX-2) in a dose-dependent manner. 1D/2D NMR and HRESIMS spectra of compound **1**–**5** (Figures S1–S35), ^1^H and ^13^C NMR spectra of compound **6**–**21** and **24**–**26** (Figures S36–S73), Dose–response curve for the compounds **1**, **15**, **17**, **21** and **25** (Figures S74–S78).

## 2. Results

### 2.1. Structure Elucidation

**Figure 2:**
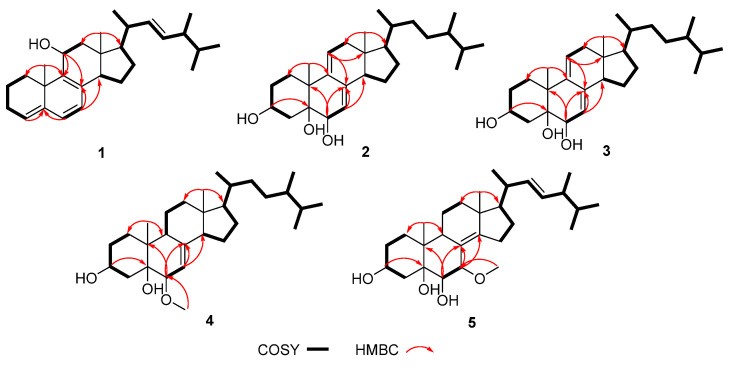
Key COSY and HMBC correlations of compounds **1**–**5**.

**Figure 3:**
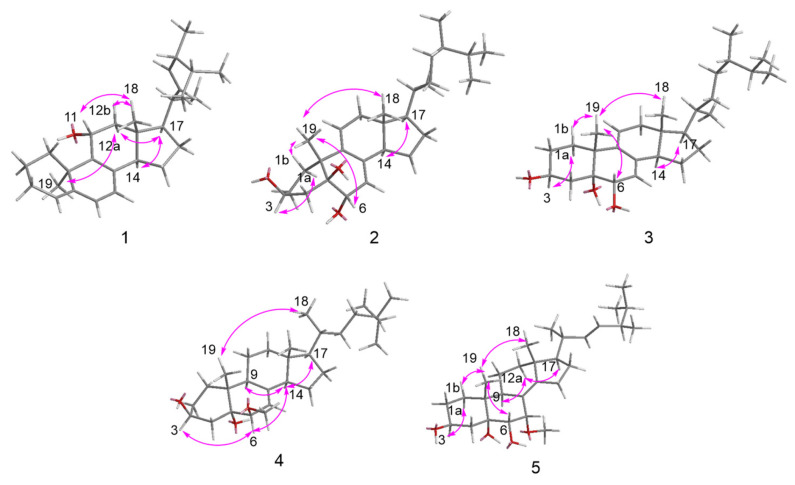
Key NOESY correlations of compounds **1**–**5**.

**Figure 4:**
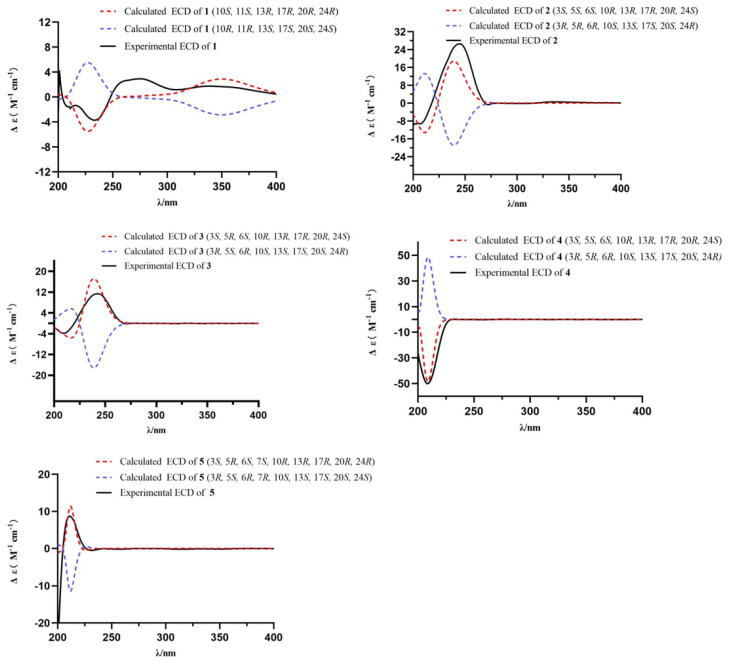
Comparison of the calculated vs experimental ECD spectra in MeOH for compounds **1**–**5**.

Compound **1** was isolated as a white powder, and its molecular formula was determined as C_28_H_42_O by HRESIMS and NMR data. The ^1^H NMR data displayed six methyl groups [*δ*_H_ 0.59 (3H, s), 0.82 (3H, d, *J* = 7.1 Hz), 0.84 (3H, d, *J* = 7.1 Hz), 0.92 (3H, d, *J* = 6.8 Hz), 1.02 (3H, d, *J* = 6.5 Hz), and 1.25 (3H, s)], one oxygenated methine 3.61 (1H, m), and five olefinic protons [*δ*_H_ 5.16 (1H, dd, *J* = 15.3, 8.2 Hz), 5.23 (1H, dd, *J* = 15.3, 7.4 Hz), 5.40 (1H, d, *J* = 5.9 Hz), 5.52 (1H, d, *J* = 6.4 Hz), and 5.68 (1H, d, *J* = 5.9 Hz)] (Table 1). The ^13^C NMR spectrum revealed 28 carbon resonances comprising six methyl groups, six methylenes, eleven methines (including an oxygenated carbon), and five quaternary carbons (Table 2). A further comprehensive analysis of the 2D NMR spectra is required. The ^1^H-^1^H COSY correlations (Figure 2) revealed the presence of four spin-coupling systems in **1**, including H_2_-1/H_2_-2/H_2_-3/H-4; H-6/H-7; H-11/H_2_-12; and H-14/H_2_-15/H_2_-16/H-17/H-20/(H_3_-21)/H-22/H-23/H-24(H_3_-28)/H-25/H_3_-26(H_3_-27). The HMBC correlations (Figure 2) from H_3_-19 to C-1, C-9, and C-10; from H-4/H-6 to C-5; from H-7 to C-8/C-14; from H-11 to C-8/C-9; and from H_3_-18 to C-12/C-13/C-17 established the carbon connectivity, and the gross structure of **1** is much similar to the ergosteroid skeleton as ergosta-4,6,8,22*E*-tetraen-11*β*-ol from *Coprinus setulosus* [12]. The only difference observed in the ^13^C NMR spectrum was that the chemical shift of C-19 in **1** (*δ*_C_ 30.6) moved downfield compared to C-19 in ergosta-4,6,8,22*E*-tetraen-11*β*-ol (*δ*_C_ 22.4). The NOE correlations (Figure 3) of H-11/H_3_-18, H-12b/H_3_-18, H-12a/H_3_-19, H-12a/H-17, and H-17/H-14 demonstrated that H_3_-19, 11-OH, H-14, and H-17 were *α*-oriented, while H_3_-18 was *β*-oriented. The double bond Δ^22,23^ was determined as *E*-configuration by the large coupling constant (*J* = 15.3 Hz). The experimental and calculated ECD spectra demonstrated a high degree of congruence (Figure 4), suggesting that the absolute configuration of **1** is 10*S*,11*S*,13*R*,17*R*,20*R*,24*R*, and named as (22*E*,24*R*,10*S*)-ergosta-4,6,8(9),22-tetraen-11*α*-ol.

Compound **2** was obtained as a white powder. Its molecular formula was determined to be C_28_H_46_O_3_ by HRESIMS data, which also indicated six degrees of unsaturation. The 1D and 2D NMR spectra revealed that compound **2** contained 28 carbons, which were classified as six methyl groups, eight methylenes, nine methines and five quaternary carbons (Table 1 and Table 2). This suggests that **2** may be an ergosteroid. The characteristic signals of two singlet methyl groups at *δ*_H_ 0.57 (3H, s) and 0.79 (3H, s), and four doublet methyls at *δ*_H_ 0.82 (3H, d, *J* = 7.0 Hz), 0.82 (3H, d, *J* = 6.8 Hz), 0.89 (3H, d, *J* = 7.0 Hz), and 0.97 (3H, d, *J* = 7.0 Hz), as observed in the ^1^H NMR, further indicated the ergosteroid skeleton of **2**. Combining with 2D NMR data (Figure 2), the structure of compound **2** was similar to the gross structure of (22*E*,24*R*)-ergosta-7,9(11),22-triene-3*β*,5*α*,6*β*-triol [13]; the only difference was that 22,23-position carbons of compound **2** were an ethylidene group. By combining NOESY spectrum and the biosynthetic pathway [13], the relative configuration of compound **2** was determined to be *rel*-3*S*,5*S*,6*S*,10*R*,13*R*,17*R*,20*R*,24*S* (Figure 3). The absolute configuration of **6** was determined by comparing the experimental and calculated ECD spectra (Figure 4). Therefore, compound **2** was elucidated as (24*R*)-ergosta-7,9(11)-diene-3*β*,5*β*,6*α*-triol.

Compound **3** had the same molecular formula (C_28_H_46_O_3_) as **2** according to their (+)-HRESIMS and the ^13^C NMR data. Through scrutiny of the 1D and 2D NMR data of **3** and **2**, only slight differences were observed for the vicinal protons and carbons adjacent to the C-5 CH group. The same HMBC correlations were reported for both compounds, implying that they are stereoisomers (Figure 2). Similar NOESY patterns and ECD data for compounds **3** and **2** indicated that the only difference between them is the configuration of C-5 (Figure 3 and Figure 4). Therefore, compound **3** was assigned as a 5-epimer of **2** and named (24*R*)-ergost-7,9(11)-dien-3*β*,5*α*,6*α*-triol.

The molecular formula of (24*R*)-6*β*-methoxyergosta-7-ene-3*β*,5*α*-diol (**4**), white powder, was determined to be C_29_H_50_O_3_ (five degrees of unsaturation) based on its HRESIMS data at *m*/*z* 469.3653 [M + Na]^+^ (calcd for C_29_H_50_O_3_Na, 469.3652). The ^1^H and ^13^C NMR data for **4** were greatly similar to those for **3** with the absence of a double bond at C-9/C-11 positions (*δ*_C_ 44.8 and 23.0) and the additional presence of a methoxy group attached at C-6 in **4** (*δ*_H_ 3.39; *δ*_C_ 58.2) (Table 1 and Table 2). This conclusion was supported by ^1^H-^1^H COSY correlation between H-9 (*δ*_H_ 2.02) and H_2_-11 (*δ*_H_ 1.56) and HMBC correlations from H-9 (*δ*_H_ 2.02) to C-7 (*δ*_C_ 116.0), C-5 (*δ*_C_ 78.0), and 6-OCH_3_ (*δ*_H_ 3.39) to C-6 (*δ*_C_ 83.9) (Figure 2). The relative configuration of **4** was consistent with **3**, as evidenced by NOESY correlations of H-3/H-6, H-6/H-14, H-9/H-14, H-14/H-17, and H_3_-18/H_3_-19 (Figure 3). The calculated ECD spectrum of 3*S*,5*S*,6*S*,10*R*,13*R*,17*R*,20*R*,24*S* matched with the experimental ECD data (Figure 4).

Compound **5** was acquired as a white powder and exhibited a [M + Na]^+^ peak at *m*/*z* 483.3447 in the HRESIMS spectrum, disclosing its molecular formula to be C_29_H_48_O_4_. Comprehensive analysis of the ^1^H and ^13^C NMR spectra (Table 1 and Table 2) for **5** disclosed that they were quite similar to those of the reported metabolite (22*E*,24*R*)-6*β*,7*α*-dimethoxyergosta-8(14),22-diene-3*β*,5*α*-diol (**6**) [13] except for signals of a methoxy group. Only one methoxyl (*δ*_H_ 3.22, *δ*_C_ 53.7) was observed in compound **5**. Through further detailed analysis of 2D NMR data, the only difference between **5** and **6** was that the methoxy group at C-6 disappeared, which was corroborated by the HMBC relationships from H-6 to C-8 and C-10, from 7-OCH_3_ to C-7, and from H-7 to C-5 and C-14 (Figure 2). The smaller coupling constants (*J*_H6,H7_ = 2.4 Hz) indicated a *cis* configuration, while the NOE-related signals between H-6 and H_3_-19 suggested both adopt the *β* configuration (Figure 3). Similarly, the double bond Δ^22,23^ was determined to be the *E* configuration, as indicated by the large coupling constants (*J* = 15.4 Hz). The absolute configuration in **5** was then affirmed as 3*S*,5*R*,6*S*,7*S*,10*R*,13*R*,17*R*,20*R*,24*R* according to the perfect similarity between the calculated and the experimental ECD spectra (Figure 4). Therefore, the structure of **5** was verified as (22*E*,24*R*)-7*α*-methoxyergosta-8(14),22-diene-3*β*,5*α*,6*α*-triol.

Twenty-one known compounds were determined as (22*E*,24*R*)-6*β*,7*α*-dimethoxyergosta-8(14),22-diene-3*β*,5*α*-diol (**6**) [13], ergosterol (**7**) [13], penicisterol F (**8**) [14], (22*E*,24*R*)-ergosta-7,9(11),22-triene-3*β*,5*β*,6*α*-triol (**9**) [13], 6*β*-methoxyergosta-7,9(11),22*E*-triene-3*β*,5*α*-diol (**10**) [15], 22*E*-7*α*-methoxy-5*α*,6*α*-epoxyergosta-8(14),22-dien-3*β*-ol (**11**) [16], ergosta-7,22-dien-6*β*-methoxy-3*β*,5*α*-diol (**12**) [17], 3*β*,5*α*,9*α*-trihydroxyergosta-7,22-dien-6-one (**13**) [18], (22*E*,24*R*)-ergosta-7,22-dien-3*β*,5*α*-diol-6-one (**14**) [13], 3*β*,5*α*,9*α*,14*α*-tetrahydroxy-(22*E*,24*R*)-ergosta-7,22-dien-6-one (**15**) [19], (22*E*,24*S*)-6-O-methyl-24-methylcholesta-7,22-diene-3*β*,5*α*,6*β*,9*α*-tetrol (**16**) [20], 3*β*-hydroxy-5,9-epoxy-(22*E*,24*R*)-ergosta-7,22-dien-6-one (**17**) [13], (22*E*,24*R*)-ergosta-7,22-dien-3*β*,5*α*-diol-6,5-olide (**18**) [21], (2*R*)-1,2-ethanediol, 1-(3-ethylphenyl)-, 1,2-dibenzoate (**19**) [22], (2*R*)-1,2-ethanediol, 1-(4-ethylphenyl)-, 1,2-dibenzoate (**20**) [22], inoscavin B (**21**) [23], (*E*)-4-(3,4-dihydroxyphenyl)but-3-en-2-one (**22**) [24], 3,4-dihydroxybenzoate (**23**) [25], 3-N-acetyl-*β*-oxotryptamine (**24**) [26], cinnamic acid bornyl ester (**25**) [27], and chrysogeside E (**26**) [28] by comparing their spectroscopic data with those reported in the literature.

### 2.2. Anti-Inflammatory Assays

**Figure 5:**
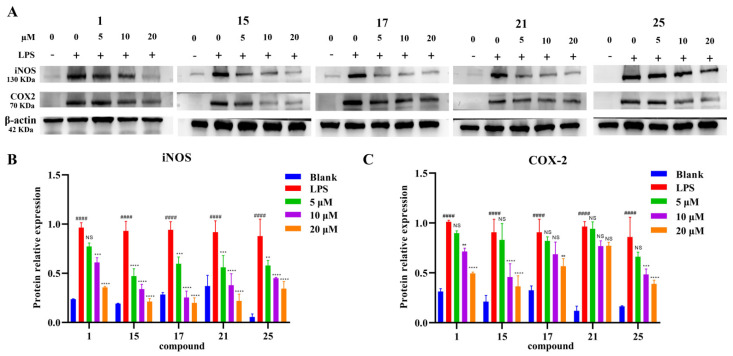
Western blotting images (**A**) and density analysis (**B**,**C**) of compounds **1**, **15**, **17**, **21** and **25** on the expression of iNOS and COX-2 in LPS-stimulated RAW264.7 cells. Compared to the control, ^####^ *p* < 0.0001. Compared to the LPS-treated group, NS: not significant, ** *p* < 0.01, *** *p* < 0.001, and **** *p* < 0.0001.

**Figure 6:**
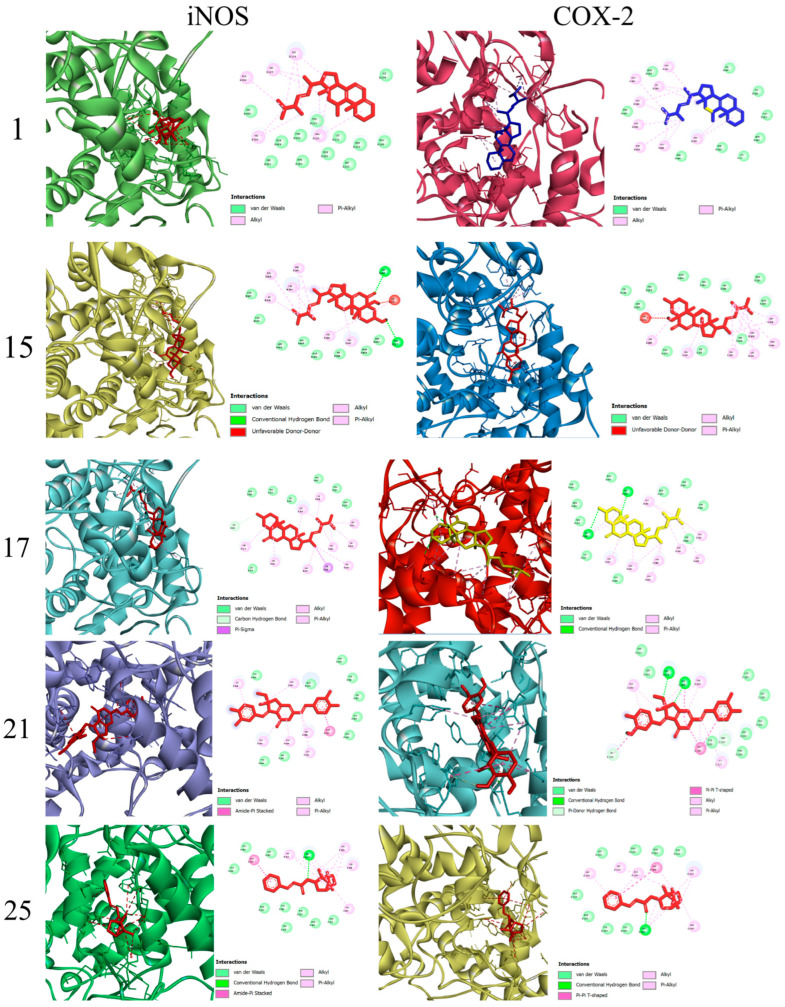
Molecular docking results of interactions between compounds **1**, **15**, **17**, **21**, and **25** and the core targets iNOS (PDB ID:4CX7) and COX-2 (PDB ID: 3VRJ).

Compounds **1**–**13**, **15**–**22** and **25** were evaluated for their anti-inflammatory activity in a classical model of LPS-induced NO release in RAW264.7 cells. All tested compounds had no obvious effect on cell viability at a concentration of 20 μM. As shown in Table 3, compounds **1**, **15**, **17**, **21** and **25** exhibited inhibitory effects against LPS-induced NO production in RAW264.7 macrophages with IC_50_ values ranging from 8.3 to 14.8 μM, being comparable to the well-known NO inhibitor, dexamethasone (IC_50_ = 9.7 μM) [29]. Compounds **15**, **17**, **21** and **25** all contain an *α*,*β*-unsaturated ketone structural moiety. As Michael acceptors, these moieties could potentially react with nucleophiles involved in NO biosynthesis in biological systems. As shown in Figure 5, a Western blotting assay revealed that compounds **1**, **15**, **17**, **21** and **25** dose-dependently inhibited LPS-induced iNOS and COX-2 expression in RAW264.7 macrophages at 5, 10, and 20 μM. Molecular docking is a theoretical simulation method that studies intermolecular interactions and predicts their binding patterns and affinity. The binding modes and forces between the compounds **1**, **15**, **17**, **21** and **25** and inflammatory factors (iNOS and COX-2) were investigated using AutoDockTools software. The results of the molecular docking analysis indicated that compounds **1**, **15**, **17**, **21** and **25** exhibited a high affinity for iNOS and COX-2, with a binding energy arrange from −8.8 to −7.2 kcal/mol (Figure 6 and Table 4).

## 3. Materials and Methods

### 3.1. General Experimental Procedures

Optical rotation (OR) was measured on a Rudolph Autopol III automatic polarimeter at 20 °C (Rudolph Research Analytical, Hackettstown, NJ, USA). Ultraviolet (UV) and experimental Circular Dichroism (ECD) spectra were recorded on a Chirascan spectrometer (Applied Photophysics Ltd., Leatherhead, UK). IR spectra were obtained on Bruker TENSOR 27 spectrometer (Bruker, Mannheim, Germany). NMR spectra were recorded on a Bruker AM-400 NMR spectrometer (Bruker, Mannheim, Germany). HRESIMS data was completed by a LC-30A + TripleTOF5600+(AB SCIEX, Framingham, MA, USA).

### 3.2. Fungal Material

The bag-cultivated sporocarps of *S. vaninii* were collected from Yulin City, Shaanxi Province, in December 2022. The strain was identified by Pro. Shuang Tian Du of College of Life Sciences and preserved in the key laboratory of chemical biology of natural products, Northwest A&F University, Yangling, Shaanxi.

### 3.3. Extraction and Isolation

The 20 kg dried fruiting bodies of *S. vaninii* were pulverized, and the powder was extracted with methanol by heating with reflux. The methanol extract was evaporated to afford the crude product (1.17 kg). The crude extract was dissolved in distilled water (2.0 L) and then extracted three times with petroleum ether (PE), dichloromethane (DCM), ethyl acetate (EA), and n-butanol (n-BuOH), successively. The EA and *n*-BuOH extracts (143.0 g) were subjected to a small pore resin coagulant gel column (MCI) with MeOH/H_2_O (40%, 60%, 80%, 100%) to get four fractions (Fr.A~Fr.D). Fr.A was separated by silica gel CC eluting stepwise with DCM-MeOH (40:1 → 2:1) to give Fr.A1-A3, and then Fr.A1 and A2 were further purified by Sephadex LH-20 (DCM/CH_3_OH, *v*/*v*, 1:1) and semi-prep RP-HPLC (CH_3_OH/H_2_O, 45%) to yield compounds **21** (23.8 min, 2.6 mg), **23** (28.6 min, 3.0 mg), **24** (25.5 min, 2.0 mg), and **25** (29.0 min, 2.5 mg). Fr.D was applied to a silica gel CC (300–400 mesh) eluting with CH_2_Cl_2_–MeOH (100:1–5:1, *v*/*v*) to afford six subfractions (Fr. D1~D6). Fr. D1 was separated with silica gel CC (PE/EA, 20:1 to 2:1) to obtain three fractions (Fr.D1-1~Fr.D1-3), and Fr.D1-3 was further purified by semipreparative HPLC (MeOH/H_2_O, 76:24, *v*/*v*) to yield compounds **1** (33.1 min, 3.8 mg) and **6** (36.1 min, 29.0 mg). Fr.D1-1 was chromatographed with silica gel CC (PE–EA, 20:1 to 2:1) to give three fractions (Fr.D1-1-1~Fr.D1-1-3), then Fr.D1-1-2 and Fr.D1-1-3 were purified by RP-HPLC (MeOH/H_2_O, 76:24, *v*/*v*) to afford compounds **22** (24.2 min, 1.5 mg), **19** (28.3 min, 4.7 mg) and **20** (33.1 min, 2.9 mg). Fraction D3 was subjected to silica gel CC (300–400 mush) eluting with DCM/MeOH (from 20:1 to 2:1, *v*/*v*) to obtain four fractions (Fr.D3-1~Fr.D3-4). After that, fraction D3-1 was separated by prep-HPLC (MeOH/H_2_O, 76:24, *v*/*v*), which produced compounds **3** (16.1 min, 4.8 mg), **8** (19.9 min, 21.0 mg), **9** (29.4 min, 3.3 mg), **10** (32.1 min, 46.0 mg), and **11** (35.7 min, 4.5 mg). Compounds **2** (24.4 min, 3.4 mg) and **7** (28.3 min, 19.0 mg) were acquired from fraction D3-2 by prep-HPLC (MeOH/H_2_O = 72:28, *v*/*v*). Fr.D4 was first subjected to a Sephadex LH-20 column (CH_3_OH/CHCl_3_, 1:1, *v*/*v*) to yield two subfractions (Fr.D4-1 and Fr.D4-2), the purification of Fr.D4-1 was chromatographed with prep-HPLC (MeOH/H_2_O = 67:33, *v*/*v*) to yield compounds **4** (21.2 min, 1.5 mg), **5** (24.8 min, 2.4 mg), **12** (26.4 min, 15.0 mg), **13** (32.1 min, 6.8 mg), **14** (27.9 min, 2.1 mg), **15** (36.6 min, 5.5 mg), and **16** (39.1 min, 28.0 mg). Compounds **17** (15.5 min, 3.7 mg) and **18** (20.8 min, 11.0 mg) were obtained from fraction D4-2 by prep-HPLC (MeOH/H_2_O = 62:38, *v*/*v*). Fr.D5 was partitioned on Sephadex LH-20 and silica gel column chromatography to get four subfractions (Fr. D5-1~Fr. D5-4). Compound **5** (22.2 min, 15.0 mg) was obtained by purifying Fr. D5-2 with semipreparative HPLC (MeOH/H_2_O = 68:32, *v*/*v*).

#### 3.3.1. Sanghusterol A = (22*E*,24*R*,10*S*)-ergosta-4,6,8(9),22-tetraen-11α-ol (**1**)

White powder; ${\left[\alpha \right]}_{D}^{20}$ −18.5 (c 0.1, MeOH); UV (MeOH): *λmax* (log ε) 225 (4.47), 256 (4.77) nm; CD (MeOH): *λmax* (Δε) 235 (−4.39), 275 (+3.48), 341 (+2.06) nm; IR *ν*_max_ 2955, 2926, 1711, 1664, 1465, 1372, 1100, 998, 869 cm^−1^; ^1^H and ^13^C NMR data, see Table 1 and Table 2; HRESIMS: *m*/*z* 395.3309 [M + H]^+^ (calcd for C_28_H_43_O, 395.3308).

#### 3.3.2. Sanghusterol B = (24*R*)-ergosta-7,9(11)-diene-3β,5β,6α-triol (**2**)

White powder; ${\left[\alpha \right]}_{D}^{20}$ +35.8 (c 0.1, MeOH); UV (MeOH): *λmax* (log ε) 248 (4.84) nm; CD (MeOH): *λmax* (Δε) 245 (+34.64) nm; IR *ν*_max_ 2967, 2880, 1707, 1455, 1376, 1221, 1061, 971 cm^−1^; ^1^H and ^13^C NMR data, see Table 1 and Table 2; HRESIMS: *m*/*z* 453.3341 [M + Na]^+^ (calcd for C_28_H_46_O_3_Na, 453.3339).

#### 3.3.3. Sanghusterol C = (24*R*)-ergost-7,9(11)-dien-3β,5α,6α-triol (**3**)

White powder; ${\left[\alpha \right]}_{D}^{20}$ +22.0 (c 0.1, MeOH); UV (MeOH): *λmax* (log ε) 248 (4.45) nm; CD (MeOH): *λmax* (Δε) 209 (−5.03), 242 (+14.82) nm; IR *ν*_max_ 2953, 2867, 1459, 1376, 1257, 1165, 1033, 983, 840 cm^−1^; ^1^H and ^13^C NMR data, see Table 1 and Table 2; HRESIMS: *m*/*z* 453.3341 [M + Na]^+^ (calcd for C_28_H_46_O_3_Na, 453.3339).

#### 3.3.4. Sanghusterol D = (24*R*)-6β-methoxyergosta-7-ene-3β,5α-diol (**4**)

White powder; ${\left[\alpha \right]}_{D}^{20}$ −41.0 (c 0.1, MeOH); UV (MeOH): *λmax* (log ε) 205 (4.55) nm; CD (MeOH): *λmax* (Δε) 208 (−67.80) nm; IR *ν*_max_ 2951, 2869, 1712, 1664, 1463, 1378, 1157, 1092, cm^−1^; ^1^H and ^13^C NMR data, see Table 1 and Table 2; HRESIMS: *m*/*z* 469.3653 [M + Na]^+^ (calcd for C_29_H_50_O_3_Na, 469.3652).

#### 3.3.5. Sanghusterol E = (22*E*,24*R*)-7α-methoxyergosta-8(14),22-diene-3β,5α,6α-triol (**5**)

White powder; ${\left[\alpha \right]}_{D}^{20}$ −0.40 (c 0.1, MeOH); UV (MeOH): *λmax* (log ε) 200 (4.89) nm; CD (MeOH): *λmax* (Δε) 214 (+11.00) nm; IR *ν*_max_ 2955, 2872, 1703, 1451, 1378, 1079, 973 cm^−1^; ^1^H and ^13^C NMR data, see Table 1 and Table 2; HRESIMS: *m*/*z* 483.3447 [M + Na]^+^ (calcd for C_29_H_48_O_4_Na, 483.3445).

### 3.4. ECD Calculation Details

Gaussian 16 software was utilized for the execution of ECD calculations. Conformational optimization was performed in the gas phase via density functional theory (DFT) at the B3LYP/6-31G(d) level. Time-dependent density functional theory (TDDFT) was also adopted for ECD calculations, which were conducted in methanol (MeOH) using the polarizable continuum model (PCM) at the B3LYP/6-311G(d,p) level. ECD spectra were generated via SpecDis 1.7 software [30].

### 3.5. Anti-Inflammatory Activity

#### 3.5.1. Cell Culture

The RAW 264.7 macrophage cell line was acquired from Procell Life Science & Technology Co., Ltd. (Wuhan, China). These cells were cultured in Dulbecco’s Modified Eagle Medium (DMEM; Gibco, Grand Island, NY, USA), which was supplemented with 10% fetal bovine serum (FBS; ABW, Shanghai Nova Pharmaceutical Technology Co., Ltd., Shanghai, China) and 1% penicillin-streptomycin (Beyotime, Shanghai Beyotime Biotechnology Co., Ltd., Shanghai, China). All cell lines were maintained at 37 °C in a humidified incubator with 5% CO_2_.

#### 3.5.2. Cell Viability

The cytotoxicity of the test compounds on RAW 264.7 macrophages was assessed using a slightly modified version of a previously described protocol [7]. RAW 264.7 cells were seeded into 96-well plates at a density of 8 × 10^3^ cells per well and incubated for 24 h, followed by exposure to the test compounds at a concentration of 20 µM for another 24 h. Dexamethasone (20 µM) served as the positive control, while 0.1% dimethyl sulfoxide (DMSO) was used as the negative control. After incubation, 3-(4,5-dimethylthiazol-2-yl)-2,5-diphenyltetrazolium bromide (MTT) was added to each well to a final concentration of 0.5 mg/mL. The insoluble formazan crystals were harvested, dissolved in DMSO, and the absorbance was quantified using a microplate reader (BioRad, Bio-Rad Laboratories, Inc., Hercules, CA, USA) at a wavelength of 490 nm.

#### 3.5.3. NO Production in LPS-Induced RAW 264.7 Macrophages

RAW 264.7 macrophages were seeded into 96-well plates at a density of 1.5 × 10^4^ cells per well and incubated for 24 h. The cells were then treated with various concentrations of the test compounds and positive control (0, 0.6125, 1.25, 2.5, 5, 10, and 20 µM) in the presence or absence of lipopolysaccharide (LPS; 1 μg/mL, Sigma-Aldrich, St. Louis, MO, USA) for 24 h. The concentration of nitric oxide (NO) in the cell culture supernatant was quantified using a Griess reagent kit (Beyotime, China). Briefly, 100 μL of the culture supernatant from each group and standard curve solutions were added to a 96-well plate, followed by the addition of 100 μL of Griess reagent. The absorbance was detected with a multifunctional microplate reader at 540 nm. All experiments were conducted in triplicate, and the data were expressed as mean ± standard deviation (SD). Dexamethasone was employed as the positive control [7].

#### 3.5.4. Western Blotting

RAW 264.7 cells were seeded into six-well plates at a density of 5 × 10^5^ cells per well and treated with compounds **1**, **15**, **17**, **21**, and **25** at different concentrations (0, 5, 10, and 20 μM), followed by stimulation with LPS (1 μg/mL) for 24 h. The cells were collected and lysed on ice for 30 min. The supernatant was obtained by centrifugation at 12,000 rpm and 4 °C for 15 min, and the protein concentration was determined using a BCA protein assay kit (BC3710, Solarbio, Beijing, China). A total of 30 μg of protein from each supernatant sample was subjected to 10% sodium dodecyl sulfate-polyacrylamide gel electrophoresis (SDS-PAGE) for separation and then transferred onto polyvinylidene fluoride (PVDF) membranes (Millipore, Burlington, MA, USA). Non-specific binding sites were blocked with TBST buffer containing 5% non-fat milk for 2 h, followed by overnight incubation at 4 °C with primary antibodies. The membranes were then washed three times with TBST buffer and incubated with secondary antibodies at room temperature for 1 h. After three additional washes with TBST buffer, immunoreactive bands were visualized using an enhanced chemiluminescence (ECL) Western blotting kit (Sigma, St. Louis, MO, USA) and quantified using ImageJ 1.52a software. The bands were scanned with a GS-800 scanner and analyzed using a ChemiDoc XRS imaging system (Bio-Rad Life Sciences, Hercules, CA, USA). The primary antibody against β-actin (60008-1-Ig) was purchased from Proteintech (Wuhan, China), whereas COX2 (cat. no. 12282) and iNOS (cat. no. 13120) antibodies were obtained from Cell Signaling Technology (Beverly, MA, USA) [7]. All experiments were conducted in triplicate.

#### 3.5.5. Molecular Docking

Molecular docking analyses were performed with PyRx 0.8 software. The X-ray crystal structures of COX-2 (PDB ID: 1CX2) and iNOS (PDB ID: 6KEY) were downloaded from the RCSB Protein Data Bank (http://www.rcsb.org/pdb/, accessed on 31 March 2026). AutoDock 4.2.6 Tools were used to process the PDB structures, which involved adding hydrogen atoms and removing water molecules, and the processed structures were saved in PDBQT format. The 3D structures of the test compounds were optimized and saved in MOL2 format, then subsequently imported into AutoDock Tools. Molecular docking was performed using the AutoDock platform (https://autodock.scripps.edu, accessed on 31 March 2026), and the binding affinities between the compounds and their target proteins were evaluated according to the binding energy values. To visualize the molecular docking results, 2D interaction diagrams of the docking poses were generated using LigPlot+ 2.3.1, and 3D representations of the binding modes were visualized with PyMOL 3.1.

### 3.6. Statistical Analysis

All data are expressed as mean ± SEM from at least three independent experiments. Statistical significance for the LPS-induced iNOS and COX-2 inhibition assays was evaluated using one-way ANOVA followed by Dunnett’s post hoc test, with the untreated control group as the reference. A *p*-value < 0.05 was considered statistically significant. Data analysis was performed using GraphPad Prism 9.0.

## 4. Conclusions

In summary, 26 compounds, including five new steroids (**1**–**5**), were isolated from *S. vaninii*. Their structure was elucidated based on detailed spectroscopic analysis and quantum chemical calculations. Concurrently, compounds **1**, **15**, **17**, **21** and **25** displayed potent inhibitory effects on LPS-induced NO production in RAW264.7 macrophages, with IC_50_ values of 8.3–14.8 μM. Mechanistically, these compounds dose-dependently downregulated the protein expression levels of iNOS and COX-2, two pivotal mediators of the inflammatory response. These findings validate the traditional medicinal application of *S. vaninii* and provide a solid phytochemical and pharmacological basis for developing *S. vaninii*-derived compounds as potential anti-inflammatory agents.

## Figures and Tables

**Figure 1:**
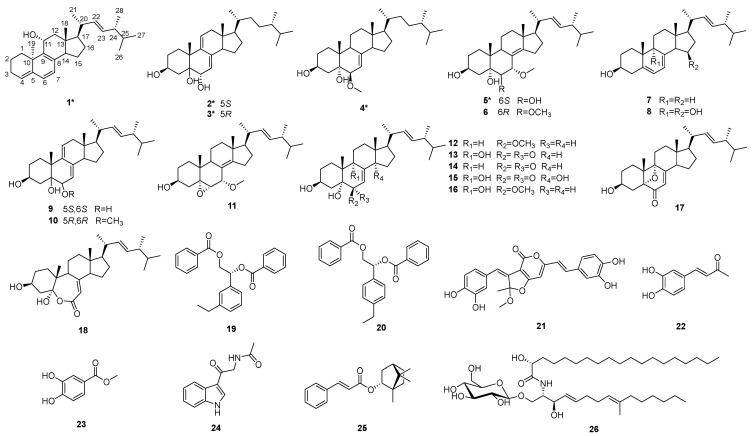
Structures of compounds **1**–**26** (*—new structures).

**Table 1:** ^1^H NMR data for compounds **1**–**5** (400 MHz, *J* in Hz).

No.	1 ^a^	2 ^a^	3 ^b^	4 ^b^	5 ^b^
1	1.51 (m), 1.72 (m)	1.51 (m), 1.72 (m)	1.70 (m), 1.88 (m)	1.60 (m),1.50 (m)	1.75 (m), 1.42 (m)
2	1.41 (m), 1.74 (m)	1.88 (m), 1.28 (m)	1.90 (m), 1.55 (m)	1.78 (m), 1.44 (m)	1.71 (m), 1.28 (m)
3	2.17 (m), 2.33 (m)	4.07 (m)	3.92 (dt, 16.1, 5.7)	3.98 (m)	3.96 (dt, 15.7, 5.7)
4	5.52 (d, 6.4)	1.71 (m),1.55 (m)	2.23 (m), 1.43 (m)	2.10 (m), 1.70 (m)	1.94 (m), 1.58 (m)
6	5.68 (d, 5.9)	4.26 (brs)	4.03 (brs)	3.17 (d, 4.9)	3.55 (d, 2.4)
7	5.40 (d, 5.9)	5.15 (brs)	5.12 (brs)	5.42 (d, 4.9)	4.13 (d, 2.4)
9				2.02 (m)	2.32 (m)
11	3.61 (m)	5.68 (m)	5.62 (d, 6.3)	1.56 (m)	1.60 (m)
12	2.47 (m), 2.38 (m)	2.35 (m), 2.18 (m)	2.38 (m), 2.18 (m)	2.10 (m), 1.33 (m)	2.02 (m), 1.20 (m)
14	2.22 (m)	2.23 (m)	2.37 (m)	1.96 (m)	
15	1.46 (m), 1.79 (m)	1.80 (m), 1.46 (m)	1.87 (m), 1.48 (m)	1.62 (m), 1.52 (m)	2.41 (m)
16	1.70 (m), 1.92 (m)	2.00 (m), 1.36 (m)	2.03 (m), 1.38 (m)	1.95 (m), 1.34 (m)	1.75 (m), 1.46 (m)
17	1.30 (m)	1.34 (m)	1.38 (m)	1.33 (m)	1.20 (m)
18	0.59 (s)	0.57 (s)	0.60 (s)	0.63 (s)	0.95 (s)
19	1.25 (s)	0.97 (s)	1.10 (s)	1.00 (s)	1.00 (s)
20	2.04 (m)	1.38 (m)	1.42 (m)	1.40 (m)	2.16 (m)
21	1.02 (d, 6.5)	0.97 (d, 6.6)	0.98 (d, 6.6)	0.98 (d, 6.6)	1.09 (d, 6.6)
22	5.16 (dd, 15.3, 8.2)	1.47 (m), 0.99 (m)	1.50 (m), 1.02 (m)	1.46 (m), 1.00 (m)	5.22 (dd, 15.4, 7.8)
23	5.23 (dd, 15.3, 7.4)	1.43 (m), 0.98 (m)	1.48 (m), 1.02 (m)	1.43 (m), 0.99 (m)	5.24 (dd, 15.4, 7.8)
24	1.87 (m)	1.21 (m)	1.25 (m)	1.24 (m)	1.86 (m)
25	1.47 (m)	1.58 (m)	1.60 (m)	1.60 (m)	1.47 (m)
26	0.82 (d, 7.1)	0.89 (d, 7.0)	0.91 (d, 7.0)	0.90 (d, 7.0)	0.86 (d, 6.8)
27	0.84 (d, 7.1)	0.82 (d, 7.0)	0.84 (d, 7.0)	0.83 (d, 7.0)	0.86 (d, 6.8)
28	0.92 (d, 6.8)	0.82 (d, 6.8)	0.84 (d, 6.8)	0.83 (d, 6.8)	0.94 (d, 6.9)
6-OCH_3_				3.39 (s)	
7-OCH_3_					3.22 (s)

^a^ Recorded in CDCl_3_. ^b^ Recorded in methanol-*d*_4_.

**Table 2:** ^13^C NMR data for compounds **1**–**5** (100 MHz).

No.	1 ^a^	2 ^a^	3 ^b^	4 ^b^	5 ^b^
1	38.5, CH_2_	30.3, CH_2_	31.2, CH_2_	33.6, CH_2_	30.2, CH_2_
2	23.0, CH_2_	32.0, CH_2_	31.7, CH_2_	31.7, CH_2_	31.6, CH_2_
3	43.0, CH_2_	68.4, CH	67.8, CH	68.3, CH	66.3, CH
4	122.6, CH	29.5, CH_2_	39.1, CH_2_	40.7, CH_2_	39.7, CH_2_
5	141.5, C	78.8, C	77.0, C	78.0, C	76.7, C
6	118.4, CH	72.5, CH	71.6, CH	83.9, CH	75.2, CH
7	115.8, CH	122.8, CH	121.7, CH	116.0, CH	80.4, CH
8	135.6, C	140.4, C	142.1, C	144.5, C	122.0, C
9	144.4, C	138.2, C	138.7, C	44.8, CH	36.6, CH
10	39.5, C	44.2, C	41.6, C	38.2, C	40.5, C
11	72.4, CH	123.5, CH	123.5, CH	23.0, CH_2_	19.0, CH_2_
12	41.7, CH_2_	43.8, CH_2_	43.7, CH_2_	40.8, CH_2_	37.3, CH_2_
13	42.3, C	43.6, C	43.6, C	44.7, C	43.5, C
14	51.2, CH	52.3, CH	52.2, CH	53.0, CH	153.3, C
15	29.1, CH_2_	24.2, CH_2_	24.2, CH_2_	24.0, CH_2_	25.2, CH_2_
16	32.4, CH_2_	29.4, CH_2_	29.5, CH_2_	28.9, CH_2_	27.1, CH_2_
17	56.3, CH	57.4, CH	57.4, CH	57.3, CH	57.2, CH
18	11.7, CH_3_	11.7, CH_3_	11.8, CH_3_	12.4, CH_3_	16.7, CH_3_
19	30.6, CH_3_	26.1, CH_3_	25.1, CH_3_	18.8, CH_3_	16.9, CH_3_
20	40.6, CH	37.6, CH	37.6, CH	37.9, CH	39.2, CH
21	20.8, CH_3_	19.1, CH_3_	19.1, CH_3_	19.5, CH_3_	20.5, CH_3_
22	135.5, CH	34.8, CH_2_	34.7, CH_2_	34.8, CH_2_	135.4, CH
23	132.2, CH	31.7, CH_2_	31.7, CH_2_	31.7, CH_2_	132.1, CH
24	42.9, CH	40.4, CH	40.4, CH	40.4, CH	43.0, CH
25	33.2, CH	32.7, CH	32.7, CH	32.7, CH	33.0, CH
26	20.1, CH_3_	20.9, CH_3_	20.9, CH_3_	20.9, CH_3_	19.1, CH_3_
27	19.8, CH_3_	18.0, CH_3_	18.0, CH_3_	18.0, CH_3_	18.7, CH_3_
28	17.7, CH_3_	15.9, CH_3_	15.9, CH_3_	15.9, CH_3_	16.8, CH_3_
6-OCH_3_				58.2, CH_3_	
7-OCH_3_					53.7, CH_3_

^a^ Recorded in CDCl_3_. ^b^ Recorded in methanol-*d*_4_.

**Table 3:** Inhibitory effects of compounds **1**–**13**, **15**–**21**, **25** and **26** on NO production in LPS-induced RAW 264.7 cells.

Comp.	IC_50_ (μM) ± SD (μM)	Comp.	IC_50_ (μM) ± SD (μM)
**1**	10.1 ± 0.1	**13**	NA
**2**	NA	**15**	8.3 ± 0.5
**3**	NA	**16**	NA
**4**	NA	**17**	10.6 ± 0.4
**5**	NA	**18**	NA
**6**	NA	**19**	NA
**7**	NA	**20**	NA
**8**	NA	**21**	8.9 ± 0.2
**9**	NA	**25**	14.8 ± 0.4
**10**	NA	**26**	NA
**11**	NA	DEX ^a^	9.7 ± 0.2
**12**	NA		

^a^ Positive control. NA: no active.

**Table 4:** Binding energy of compounds **1**, **15**, **17**, **21**, and **25** with iNOS and COX-2.

Compound	Binding Energy	
	iNOS	COX-2
**1**	−7.7	−8.2
**15**	−8.1	−8.4
**17**	−7.2	−8.8
**21**	−7.2	−8.2
**25**	−7.9	−8.2

## Data Availability

The original contributions presented in this study are included in the article/Supplementary Materials. Further inquiries can be directed to the corresponding authors.

## Supplementary Materials

The following supporting information can be downloaded at: https://www.mdpi.com/article/10.3390/ijms27073315/s1.
