# Supplementary material for: Ergosteroid and Phenolic Constituents from the Mushroom Sanghuangporus vaninii with Anti-Inflammatory Activity
Source: Int J Mol Sci. 2026 Apr 7;27(7):3315. doi: 10.3390/ijms27073315 (PMC13073346; doi:10.3390/ijms27073315)
Supplement: Supplementary file 1 [file ijms-27-03315-s001.zip › ijms-4124575-supplementary.pdf]

Ergosteroid and Phenolic Constituents from the Mushroom *Sanghuangporus vaninii* with Anti-Inflammatory Activity

Yu-Xin Gao [1,†], Yue-Tong Zhu [2,†], Almutamad Sheikho [2], Qiu-Yu Zhao [1], Ya-Ru Wang [1], Yong-Hua Wang [1,*], Yu-Qi Gao [3,*] and Jin-Ming Gao [2]

[1] College of Life Sciences, Northwest University, Xi'an 710069, China
[2] Shaanxi Key Laboratory of Natural Products & Chemical Biology, College of Chemistry & Pharmacy, Northwest A&F University, Yangling 712100, China
[3] College of Food Science and Technology, Northwest University, Xi'an 710069, China
* Correspondence: yhwang@nwu.edu.cn (Y.-H.W.); gyq1225@nwu.edu.cn (Y.-Q.G.)
† These authors contributed equally to this work.

List of supporting information

Figure S1. ^{1}H NMR spectrum of compound **1** (400MHz, $CDCl_3$)

Figure S2. ^{13}C NMR spectrum of compound **1** (100MHz, $CDCl_3$)

Figure S3. ^{1}H-^{1}H COSY spectrum of compound **1**

Figure S4. HSQC spectrum of compound **1**

Figure S5. HMBC spectrum of compound **1**

Figure S6. NOESY spectrum of compound **1**

Figure S7. HR-ESI-MS spectrum of compound **1**

Figure S8. ^{1}H NMR spectrum of compound **2** (400MHz, $CDCl_3$)

Figure S9. ^{13}C NMR spectrum of compound **2** (100MHz, $CDCl_3$)

Figure S10. ^{1}H-^{1}H COSY spectrum of compound **2**

Figure S11. HSQC spectrum of compound **2**

Figure S12. HMBC spectrum of compound **2**

Figure S13. NOESY spectrum of compound **2**

Figure S14. HR-ESI-MS spectrum of compound **2**

Figure S15. ^{1}H NMR spectrum of compound **3** (400MHz, methanol-d_4)

Figure S16. ^{13}C NMR spectrum of compound **3** (100MHz, methanol-d_4)

Figure S17. ^{1}H-^{1}H COSY spectrum of compound **3**

Figure S18. HSQC spectrum of compound **3**

Figure S19. HMBC spectrum of compound **3**

Figure S20. NOESY spectrum of compound **3**

Figure S21. HR-ESI-MS spectrum of compound **3**

Figure S22. ^{1}H NMR spectrum of compound **4** (400MHz, methanol-d_4)

Figure S23. ^{13}C NMR spectrum of compound **4** (100MHz, methanol-d_4)

Figure S24. ^{1}H-^{1}H COSY spectrum of compound **4**

Figure S25. HSQC spectrum of compound **4**

Figure S26. HMBC spectrum of compound **4**

Figure S27. NOESY spectrum of compound **4**

Figure S28. HR-ESI-MS spectrum of compound **4**

Figure S29. ^{1}H NMR spectrum of compound **5** (400MHz, methanol-d_4)

Figure S30. ^{13}C NMR spectrum of compound **5** (100MHz, methanol-d_4)

Figure S31. ^{1}H-^{1}H COSY spectrum of compound **5**

Figure S32. HSQC spectrum of compound **5**

Figure S33. HMBC spectrum of compound **5**

Figure S34. NOESY spectrum of compound **5**

Figure S35. HR-ESI-MS spectrum of compound **5**

Figure S36. ^{1}H NMR spectrum of compound **6** (400MHz, methanol-d_4)

Figure S37. ^{13}C NMR spectrum of compound **6** (100MHz, methanol-d_4)

Figure S38. ^{1}H NMR spectrum of compound **7** (400MHz, $CDCl_3$)

Figure S39. ^{13}C NMR spectrum of compound **7** (100MHz, $CDCl_3$)

Figure S40. ^{1}H NMR spectrum of compound **8** (400MHz, methanol-d_4)

Figure S41. ^{13}C NMR spectrum of compound **8** (100MHz, methanol-d_4)

Figure S42. ^{1}H NMR spectrum of compound **9** (400MHz, methanol-d_4)

Figure S43. ^{13}C NMR spectrum of compound **9** (100MHz, methanol-d_4)

Figure S44. ^{1}H NMR spectrum of compound **10** (400MHz, methanol-d_4)

Figure S45. ^{13}C NMR spectrum of compound **10** (100MHz, methanol-d_4)

Figure S46. ^{1}H NMR spectrum of compound **11** (400MHz, methanol-d_4)

Figure S47. ^{13}C NMR spectrum of compound **11** (100MHz, methanol-d_4)

Figure S48. ^{1}H NMR spectrum of compound **12** (400MHz, DMSO-d_6)

Figure S49. ^{13}C NMR spectrum of compound **12** (100MHz, DMSO-d_6)

Figure S50. ^{1}H NMR spectrum of compound **13** (400MHz, DMSO-d_6)

Figure S51. ^{13}C NMR spectrum of compound **13** (100MHz, DMSO-d_6)

Figure S52. ^{1}H NMR spectrum of compound **14** (400MHz, DMSO-d_6)

Figure S53. ^{13}C NMR spectrum of compound **14** (100MHz, DMSO-d_6)

Figure S54. ^{1}H NMR spectrum of compound **15** (400MHz, $CDCl_3$)

Figure S55. ^{13}C NMR spectrum of compound **15** (100MHz, $CDCl_3$)

Figure S56. ^{1}H NMR spectrum of compound **16** (400MHz, methanol-d_4)

Figure S57. ^{13}C NMR spectrum of compound **16** (100MHz, methanol-d_4)

Figure S58. ^{1}H NMR spectrum of compound **17** (400MHz, methanol-d_4)

Figure S59. ^{13}C NMR spectrum of compound **17** (100MHz, methanol-d_4)

Figure S60. ^{1}H NMR spectrum of compound **18** (400MHz, methanol-d_4)

Figure S61. ^{13}C NMR spectrum of compound **18** (100MHz, methanol-d_4)

Figure S62. ^{1}H NMR spectrum of compound **19** (400MHz, $CDCl_3$)

Figure S63. ^{13}C NMR spectrum of compound **19** (100MHz, $CDCl_3$)

Figure S64. ^{1}H NMR spectrum of compound **20** (400MHz, $CDCl_3$)

Figure S65. ^{13}C NMR spectrum of compound **20** (100MHz, $CDCl_3$)

Figure S66. ^{1}H NMR spectrum of compound **21** (400MHz, methanol-d_4)

Figure S67. ^{13}C NMR spectrum of compound **21** (100MHz, methanol-d_4)

Figure S68. ^{1}H NMR spectrum of compound **24** (400MHz, methanol-d_4)

Figure S69. ^{13}C NMR spectrum of compound **24** (100MHz, methanol-d_4)

Figure S70. ^{1}H NMR spectrum of compound **25** (400MHz, $CDCl_3$)

Figure S71. ^{13}C NMR spectrum of compound **25** (100MHz, $CDCl_3$)

Figure S72. ^{1}H NMR spectrum of compound **26** (400MHz, DMSO-d_6)

Figure S73. ^{13}C NMR spectrum of compound **26** (100MHz, DMSO-d_6)

Figure S74. Dose–response curve for the compound **1**.

Figure S75. Dose–response curve for the compound **15**.

Figure S76. Dose–response curve for the compound **17**.

Figure S77. Dose–response curve for the compound **21**.

Figure S78. Dose–response curve for the compound **25**.

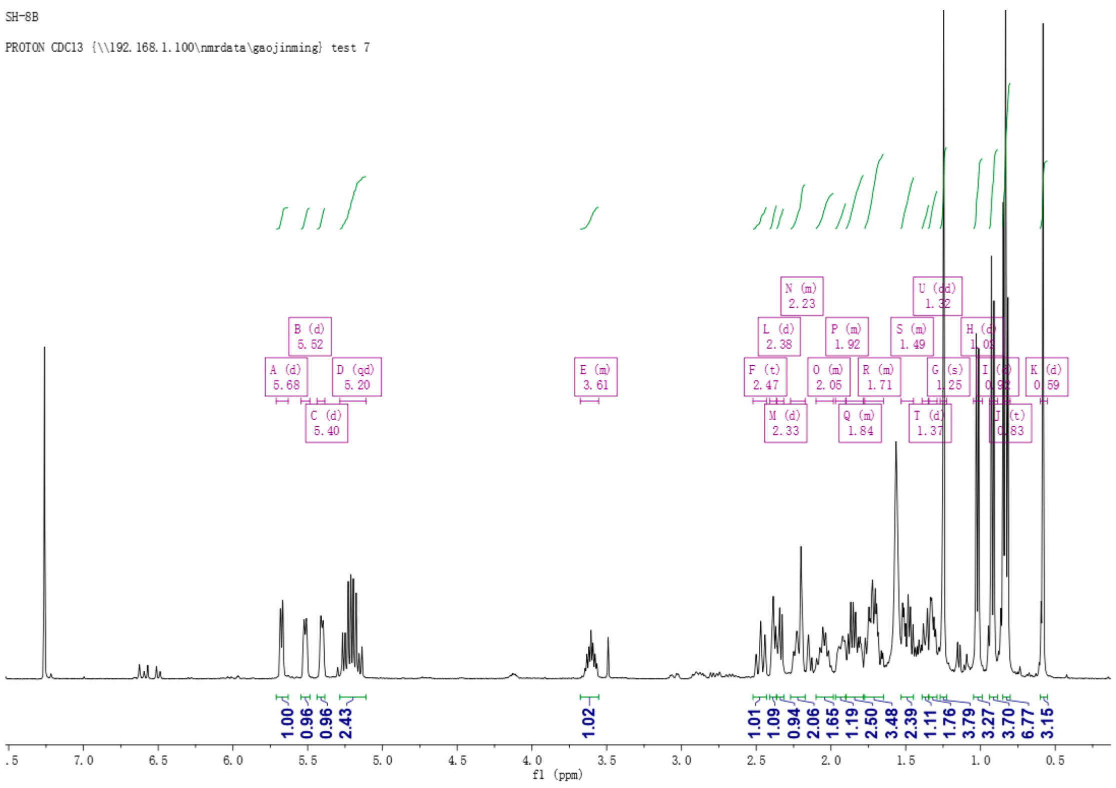

Figure S1. ^{1}H NMR spectrum of compound **1** (400MHz, $CDCl_3$)

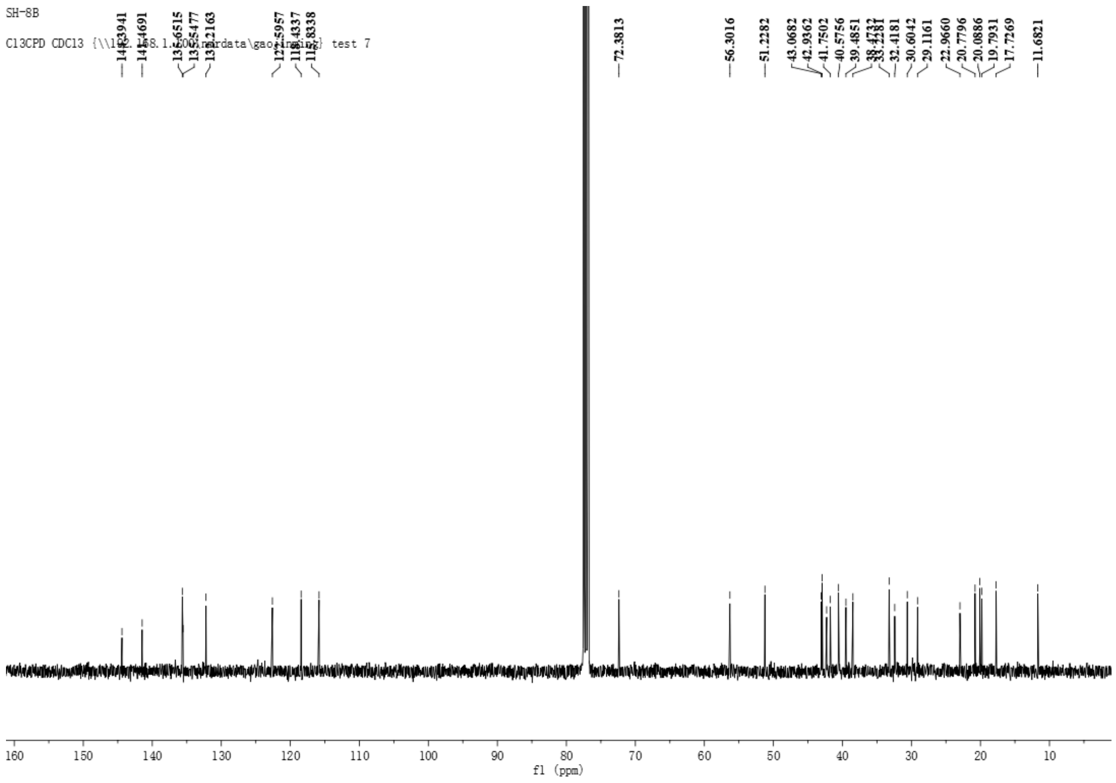

Figure S2. ^{13}C NMR spectrum of compound **1** (400MHz, $CDCl_3$)

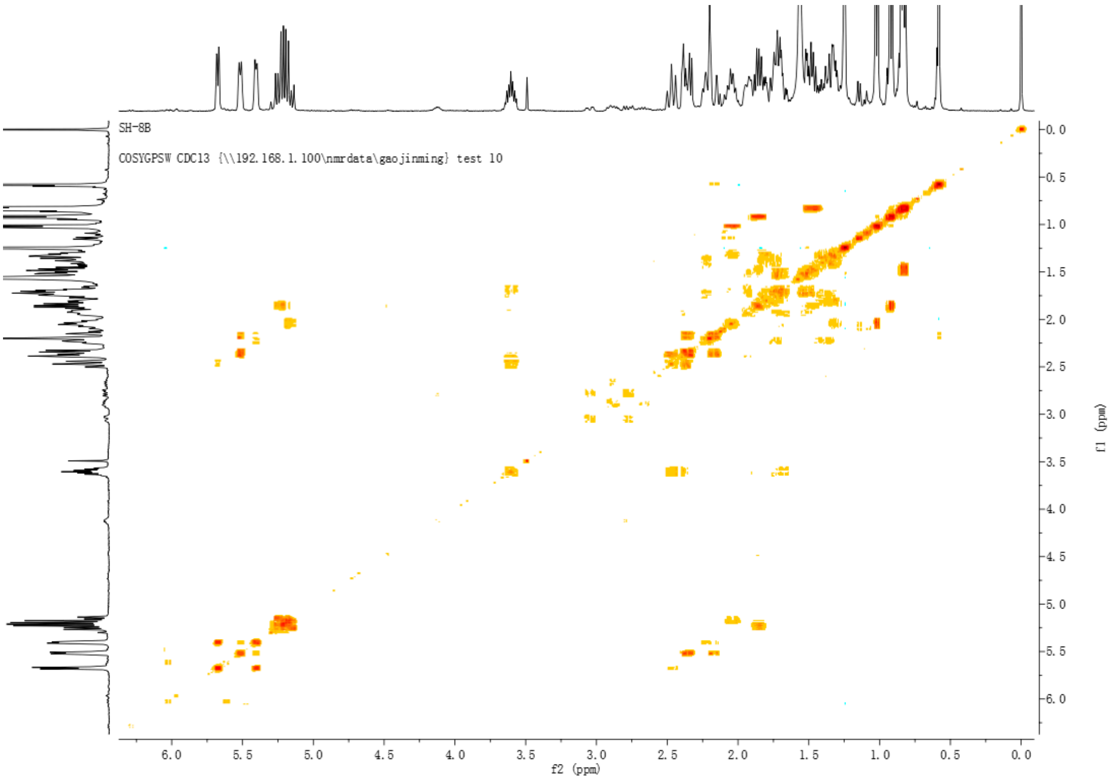

Figure S3. ^{1}H-^{1}H COSY spectrum of compound **1**

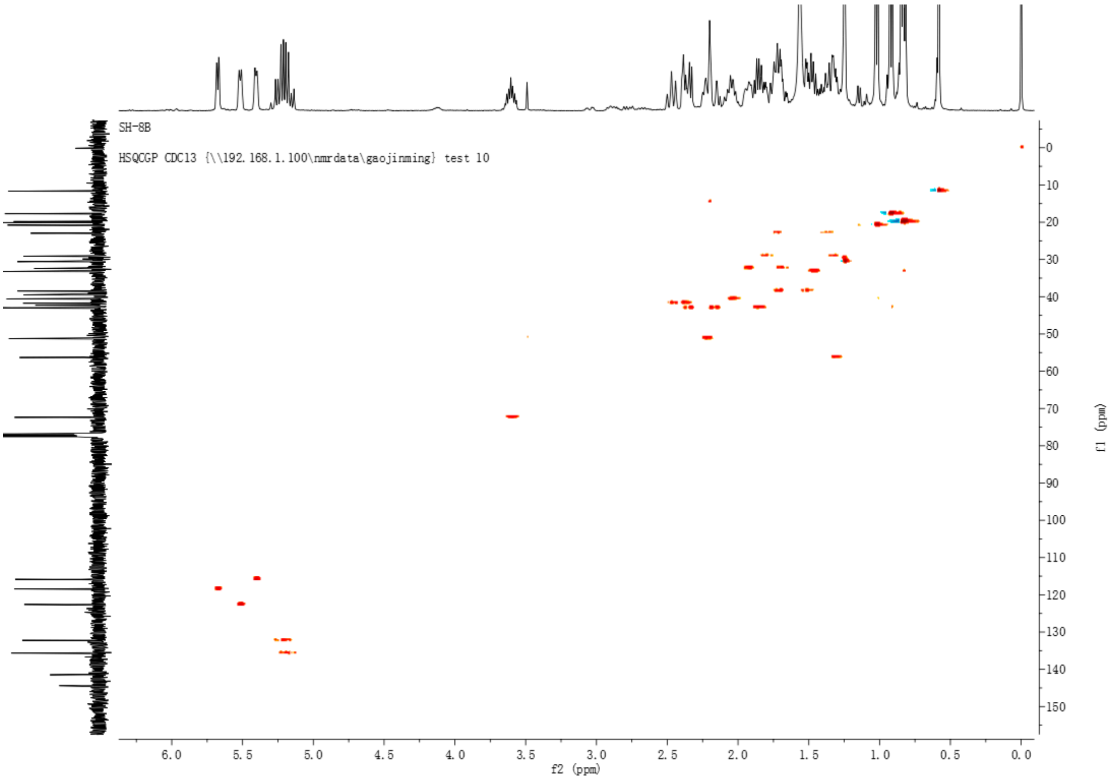

Figure S4. HSQC spectrum of compound **1**

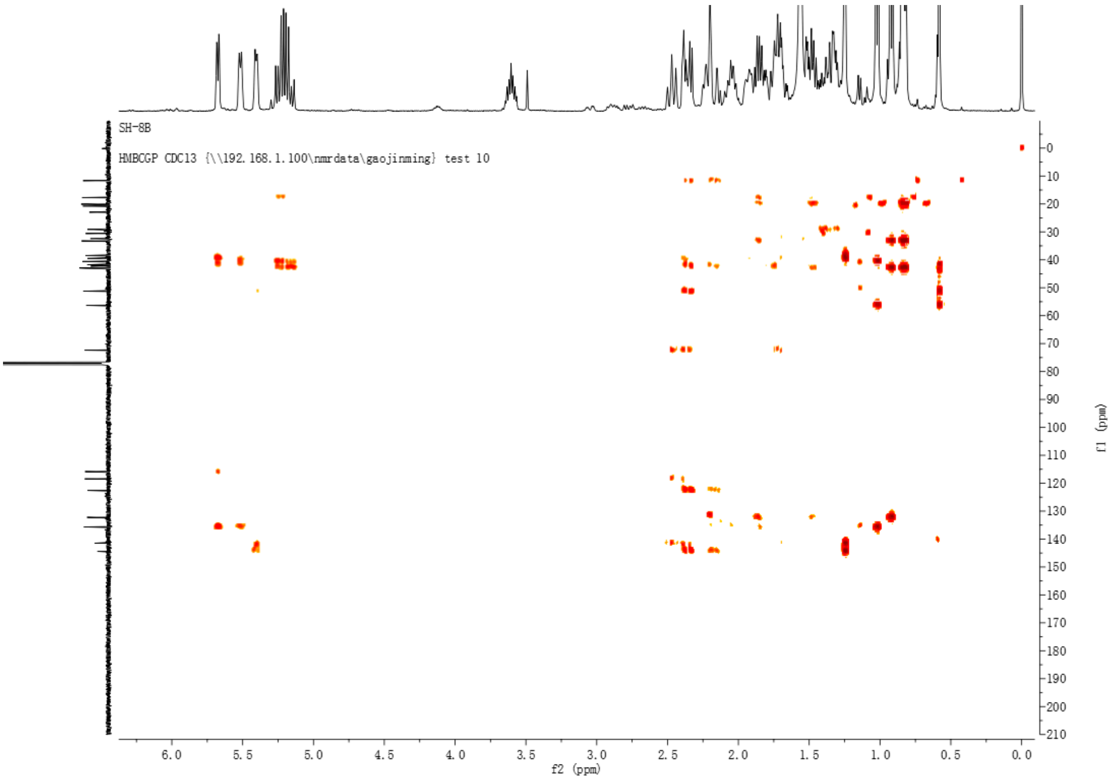

Figure S5. HMBC spectrum of compound **1**

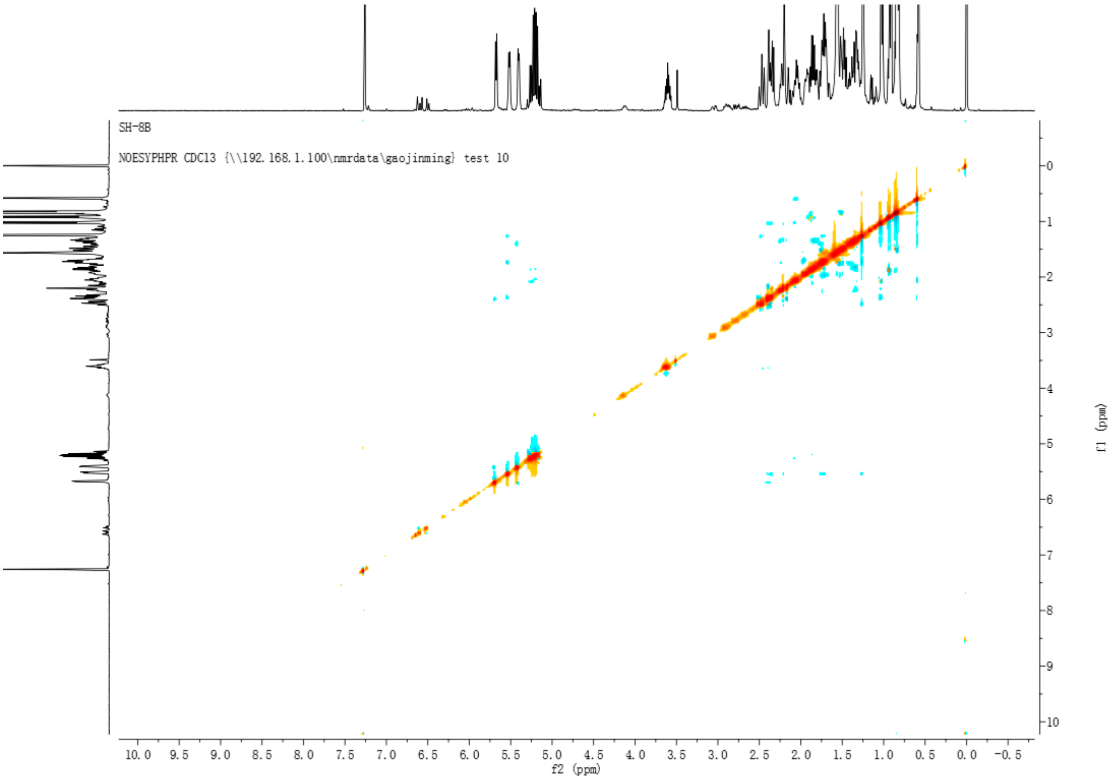

Figure S6. NOESY spectrum of compound **1**

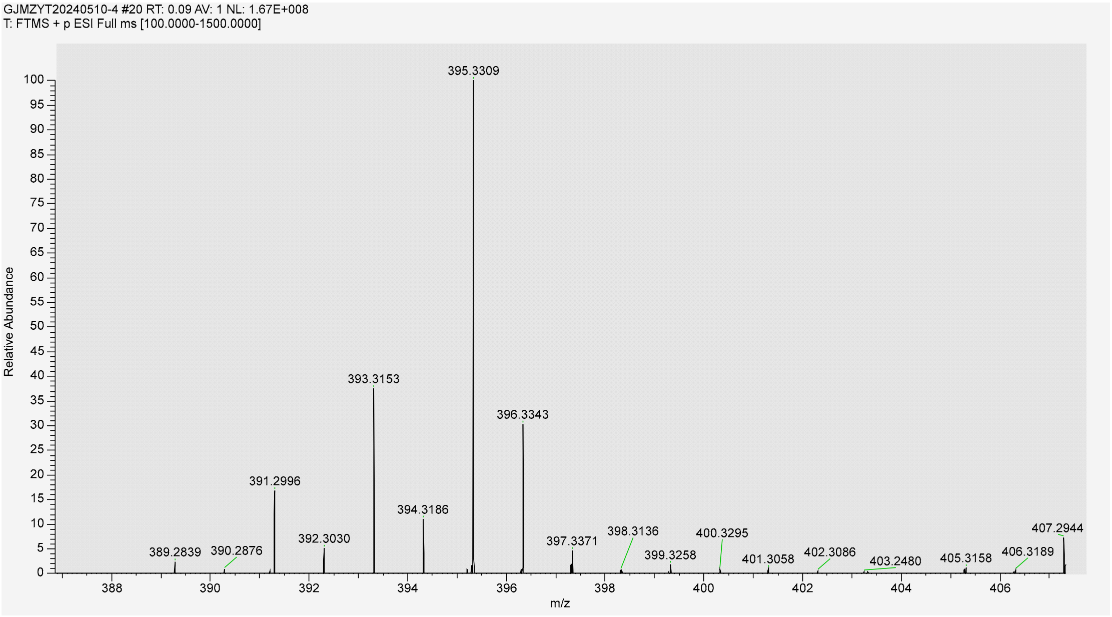

Figure S7. HRESIMS spectrum of compound **1**

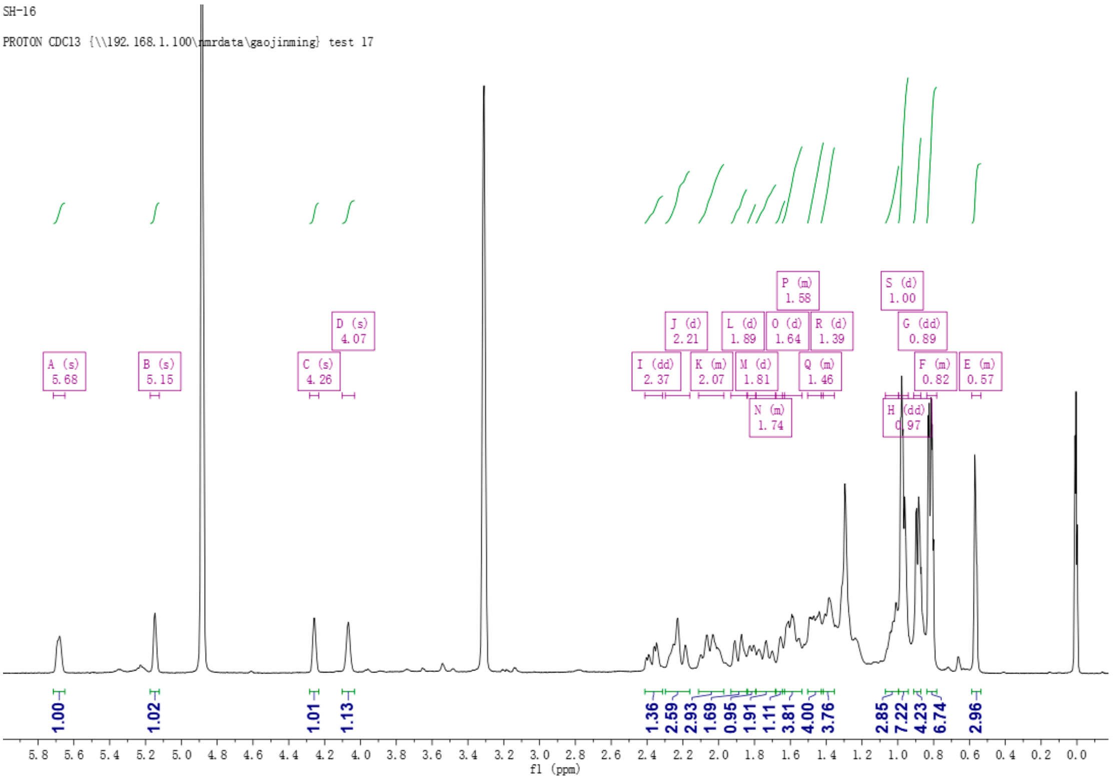

Figure S8. ^{1}H NMR spectrum of compound **2** (400MHz, $CDCl_3$)

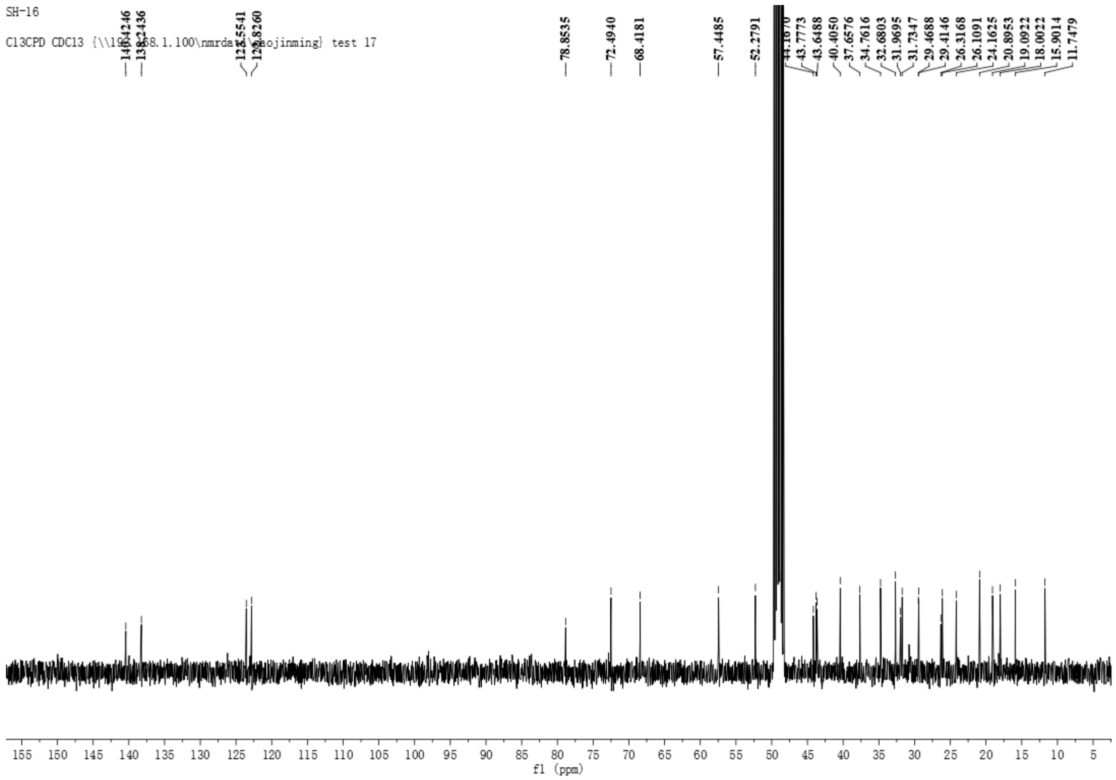

Figure S9. ^{13}C NMR spectrum of compound **2** (400MHz, $CDCl_3$)

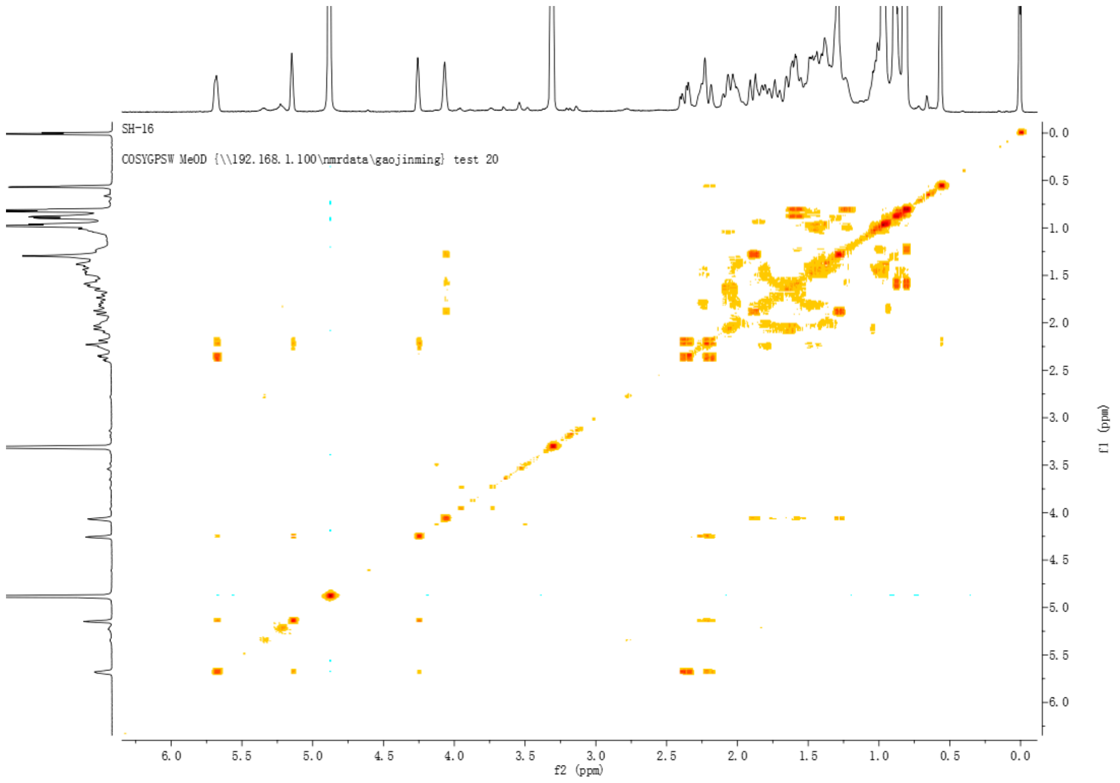

Figure S10. ^1H-^1H COSY spectrum of compound **2**

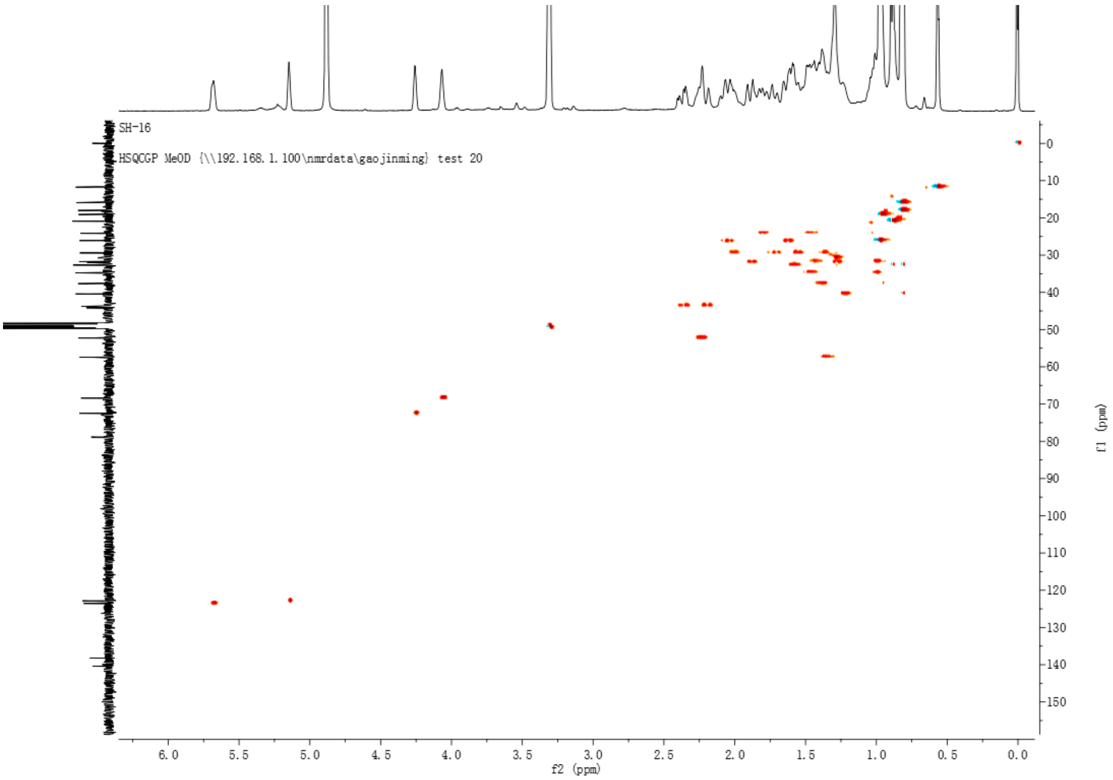

Figure S11. HSQC spectrum of compound **2**

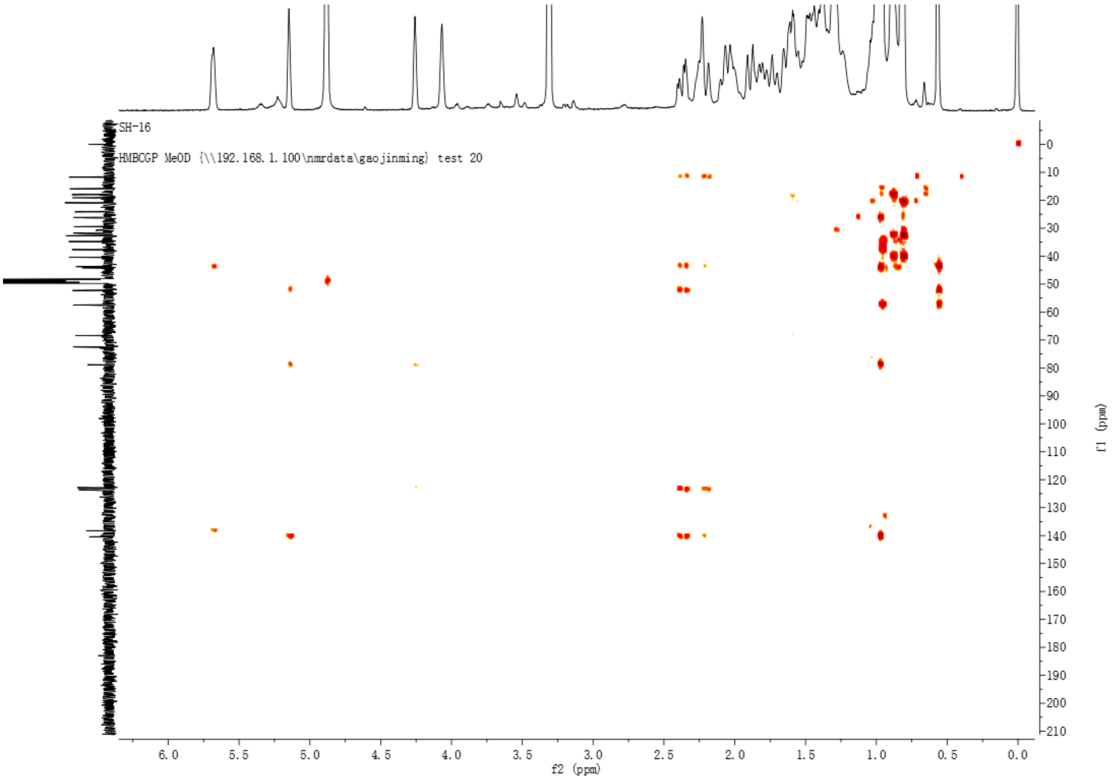

Figure S12. HMBC spectrum of compound **2**

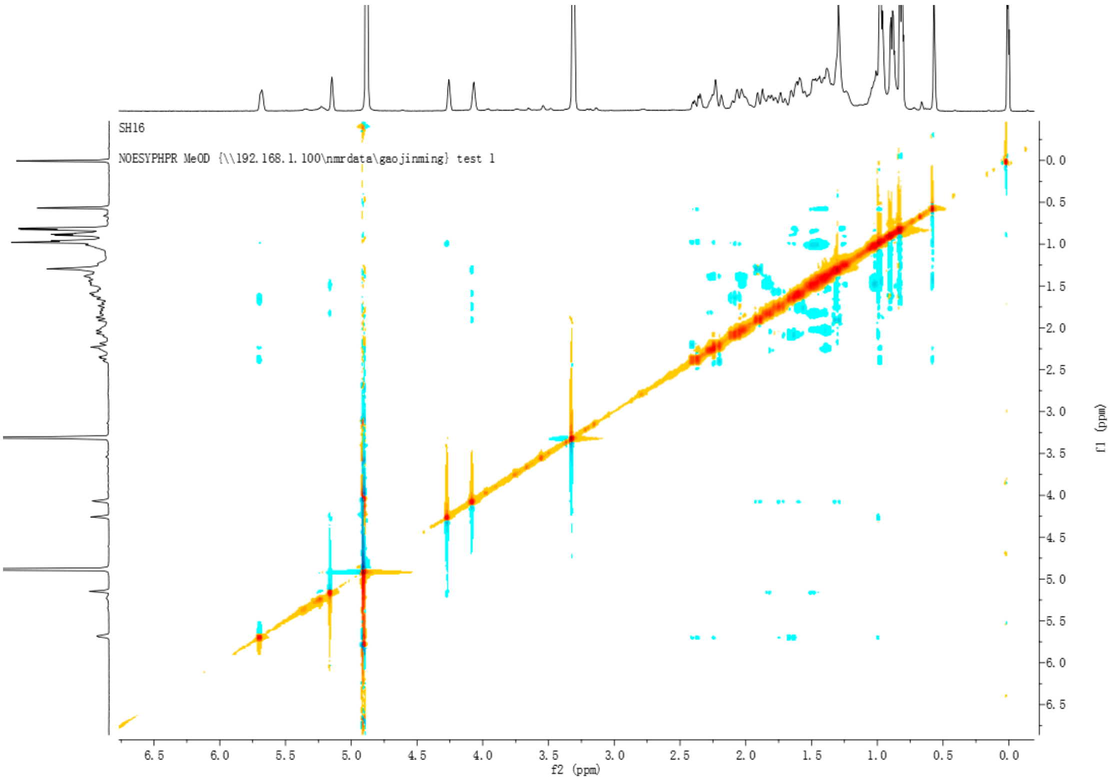

Figure S13. NOESY spectrum of compound **2**

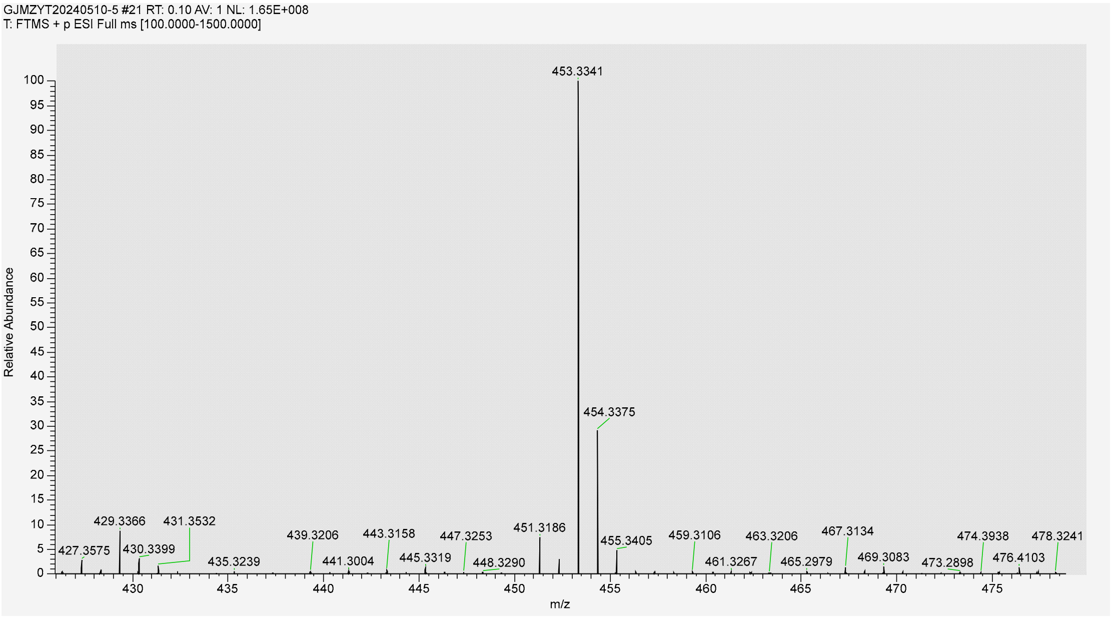

Figure S14. HRESIMS spectrum of compound **2**

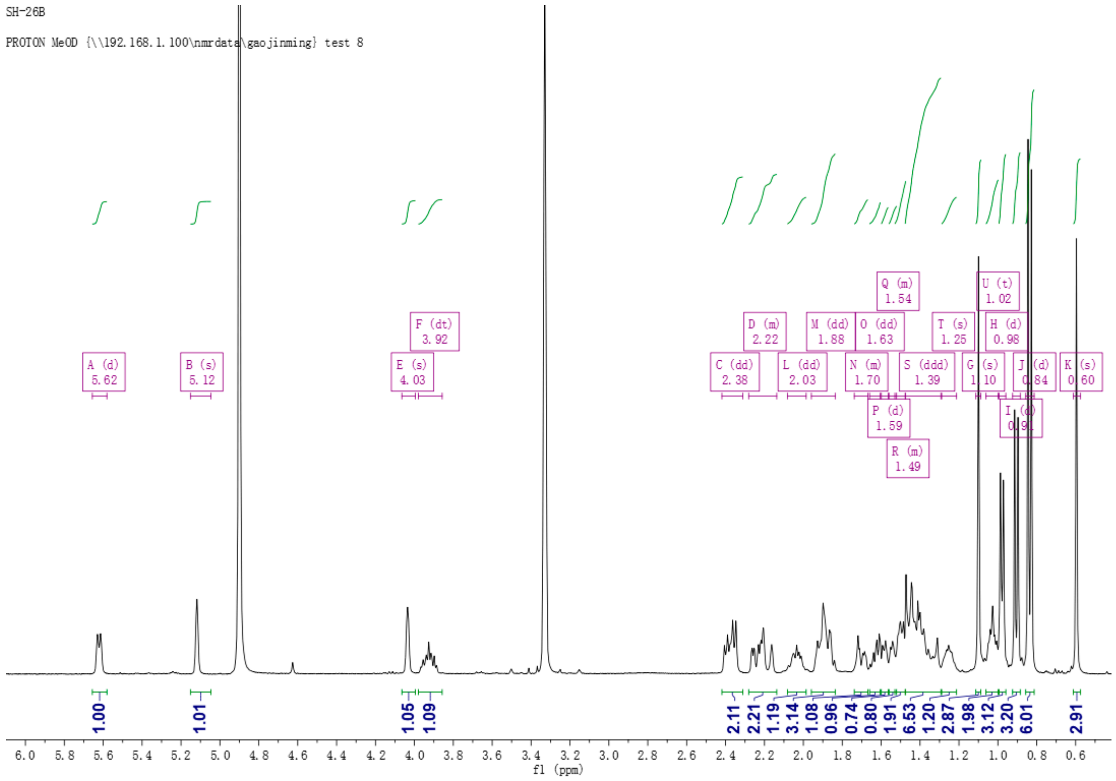

Figure S15. ^{1}H NMR spectrum of compound **3** (400MHz, methanol-d_4)

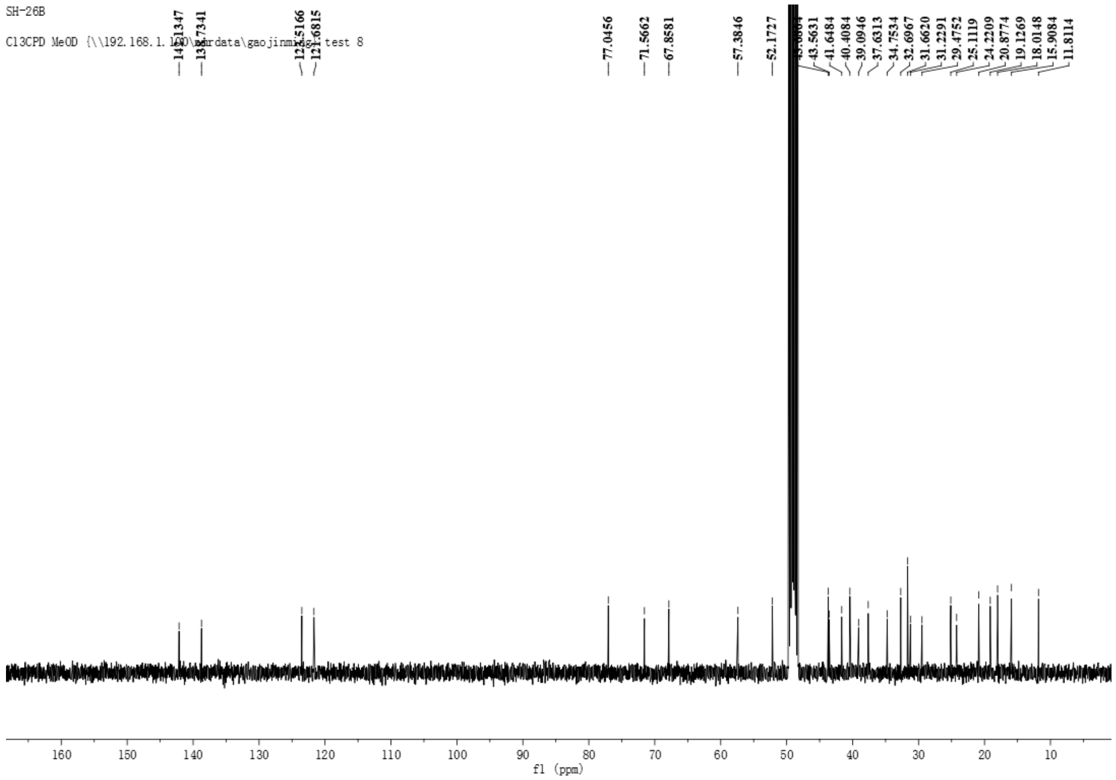

Figure S16. ^{13}C NMR spectrum of compound **3** (400MHz, methanol-d_4)

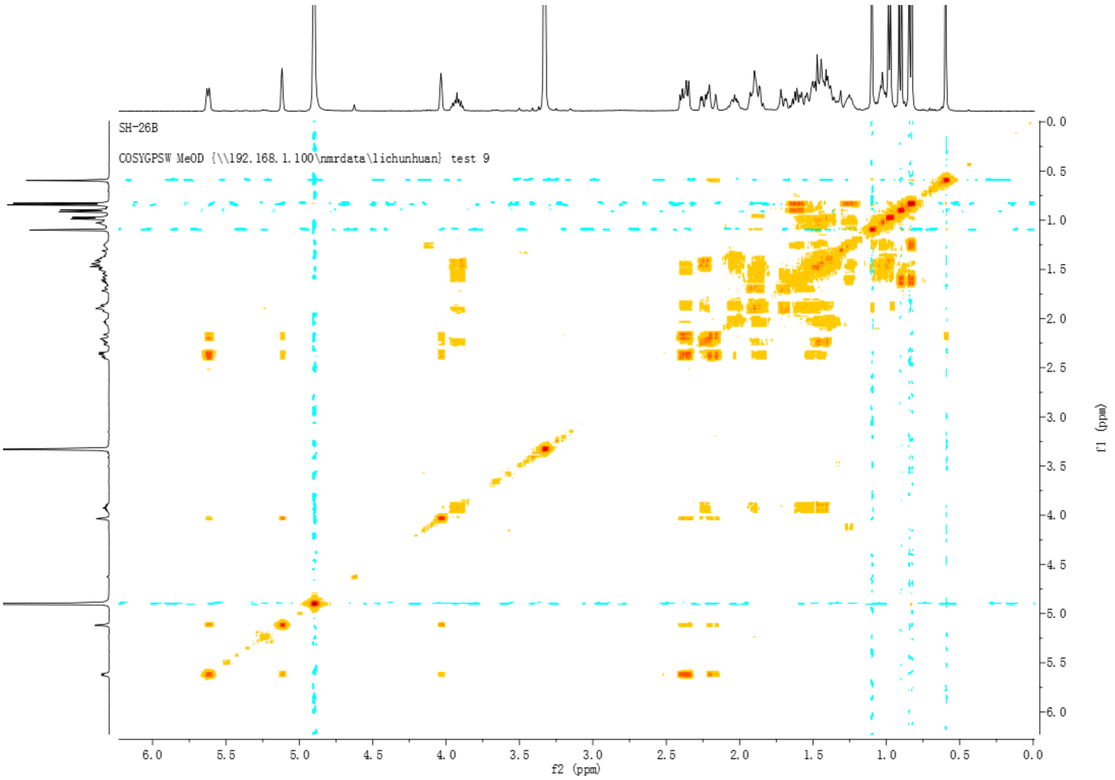

Figure S17. ^{1}H-^{1}H COSY spectrum of compound **3**

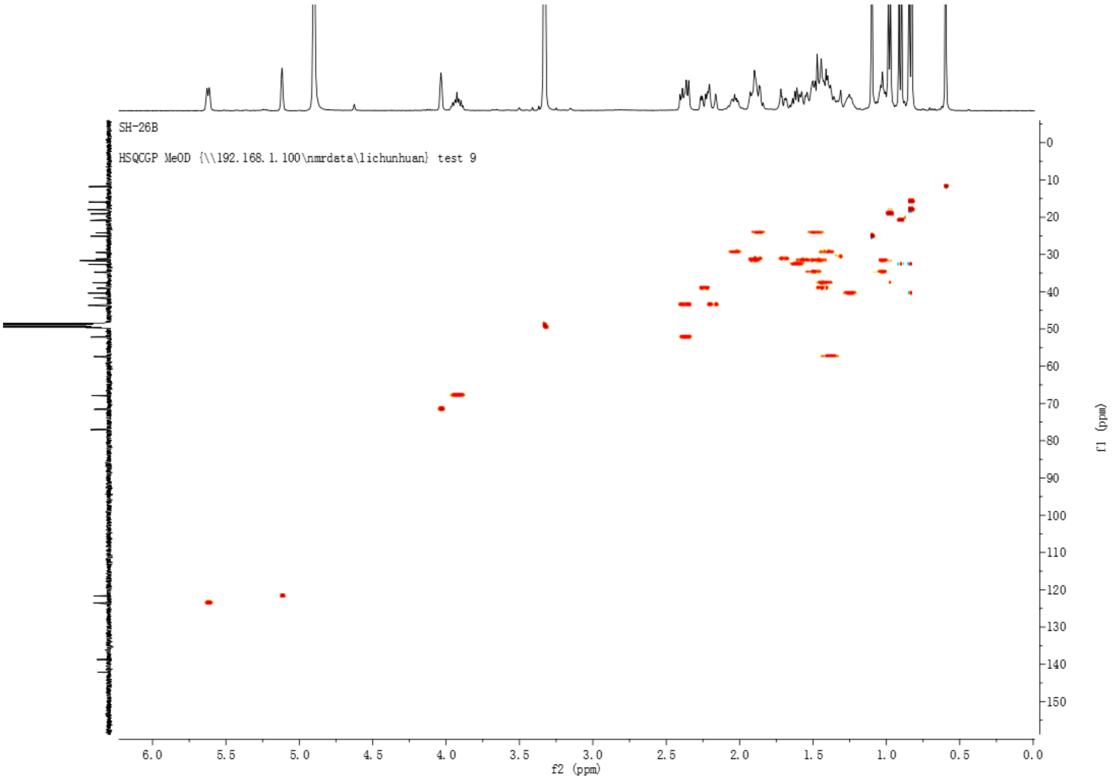

Figure S18. HSQC spectrum of compound **3**

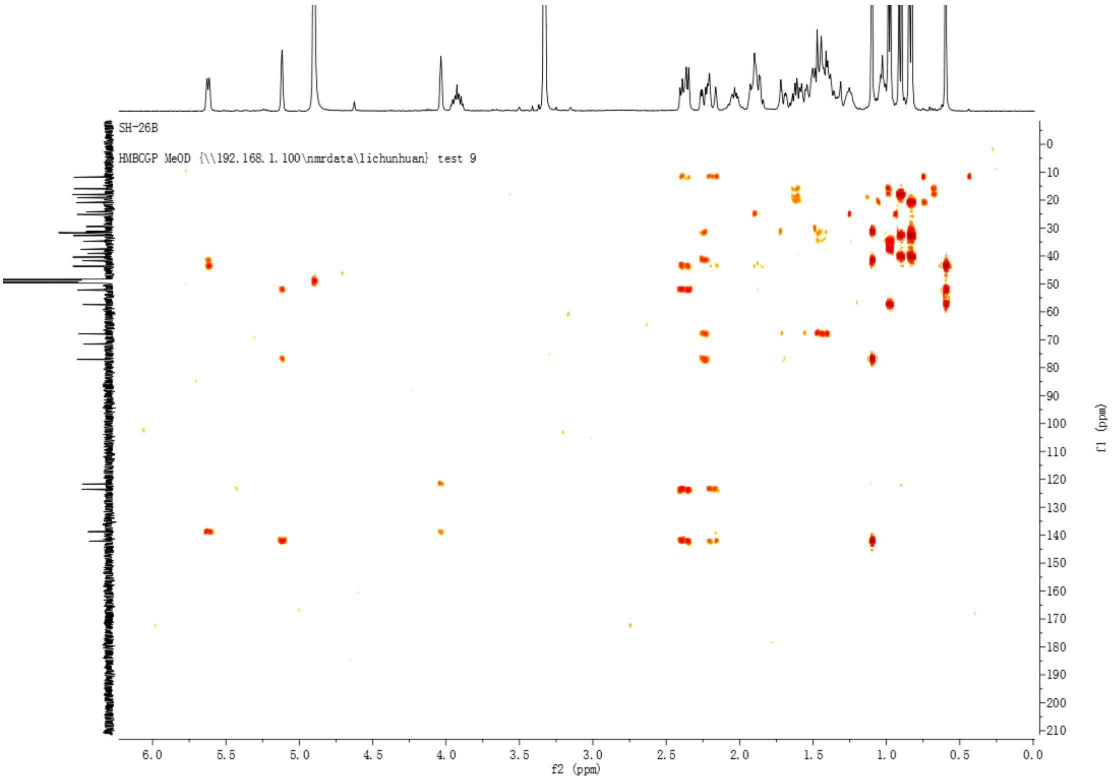

Figure S19. HMBC spectrum of compound **3**

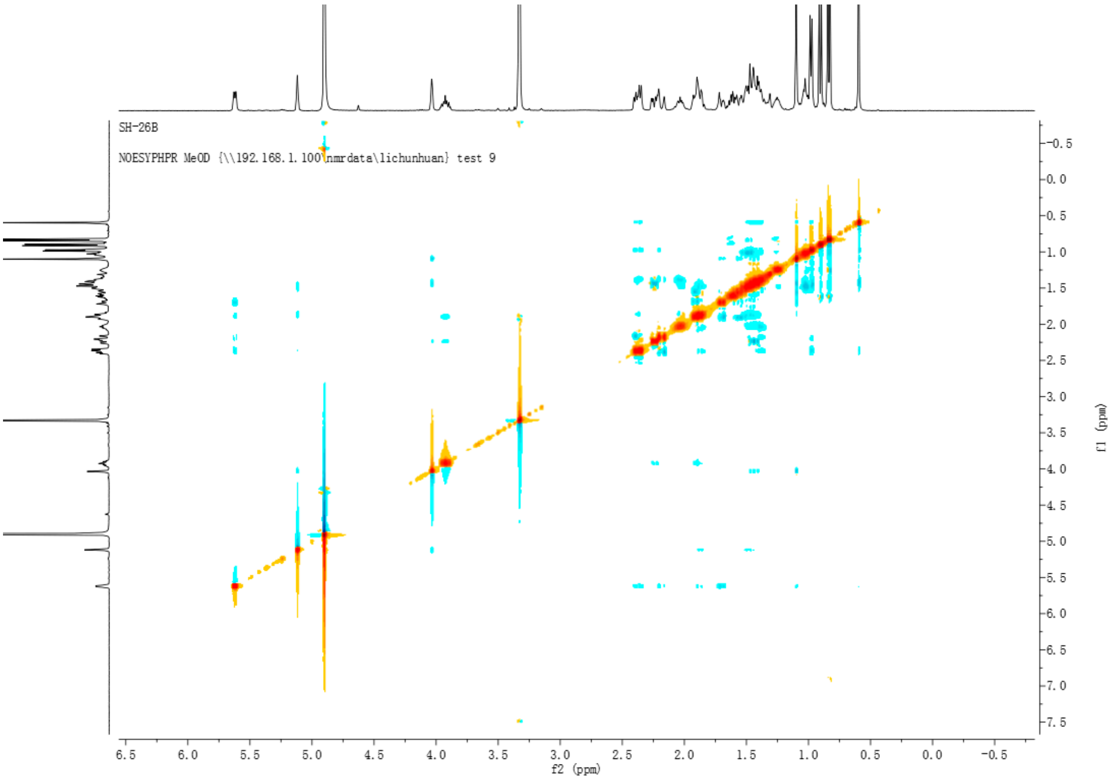

Figure S20. NOESY spectrum of compound **3**

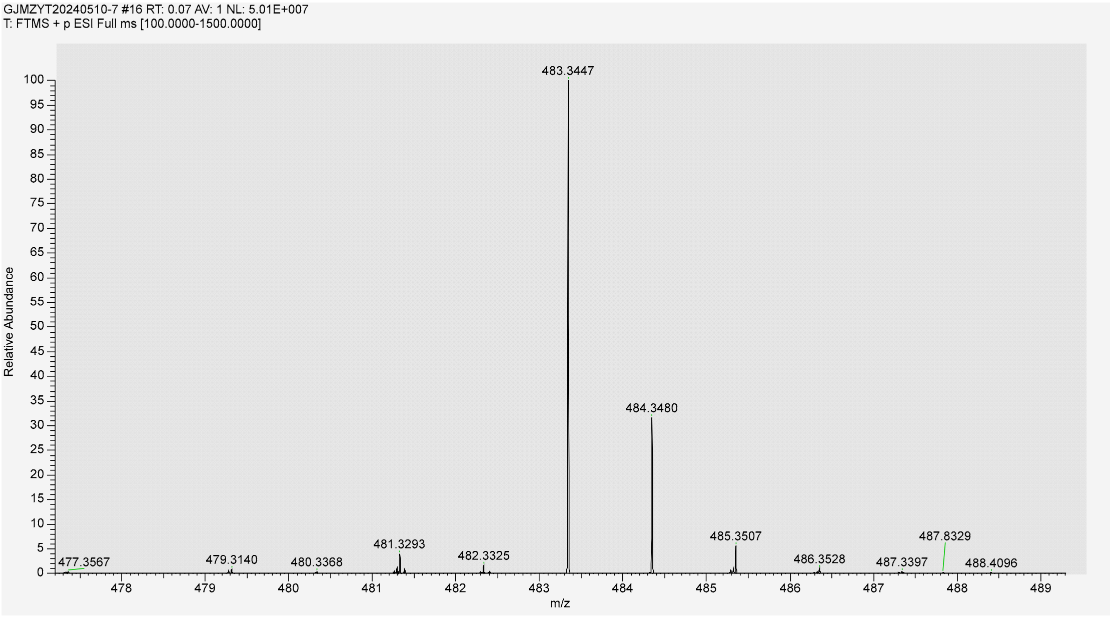

Figure S21. HRESIMS spectrum of compound **3**

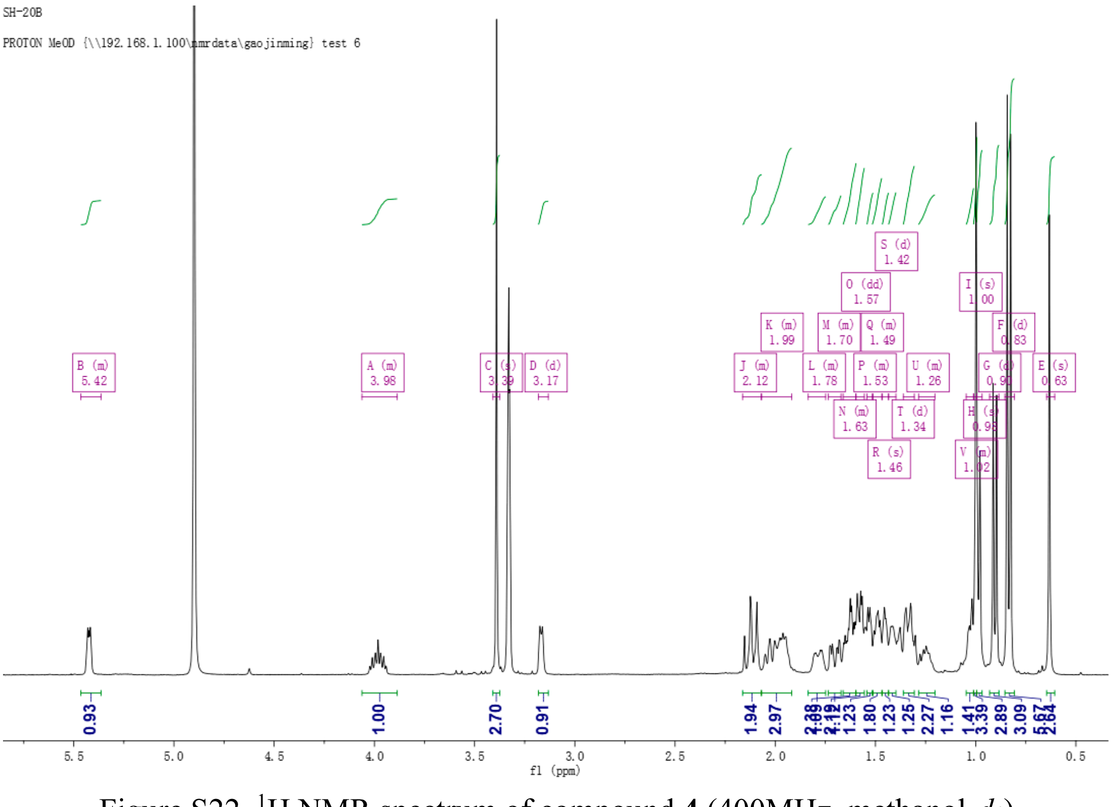

Figure S22. ^{1}H NMR spectrum of compound **4** (400MHz, methanol-d_4)

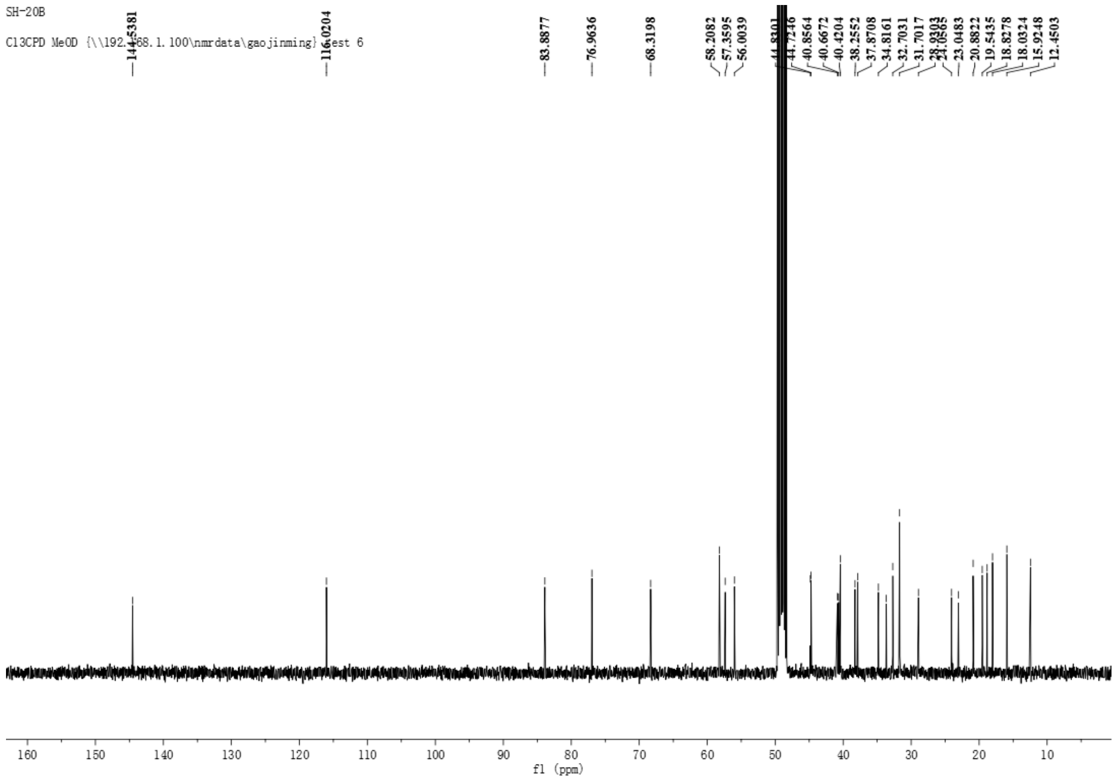

Figure S23. ^{13}C NMR spectrum of compound **4** (400MHz, methanol-d_4)

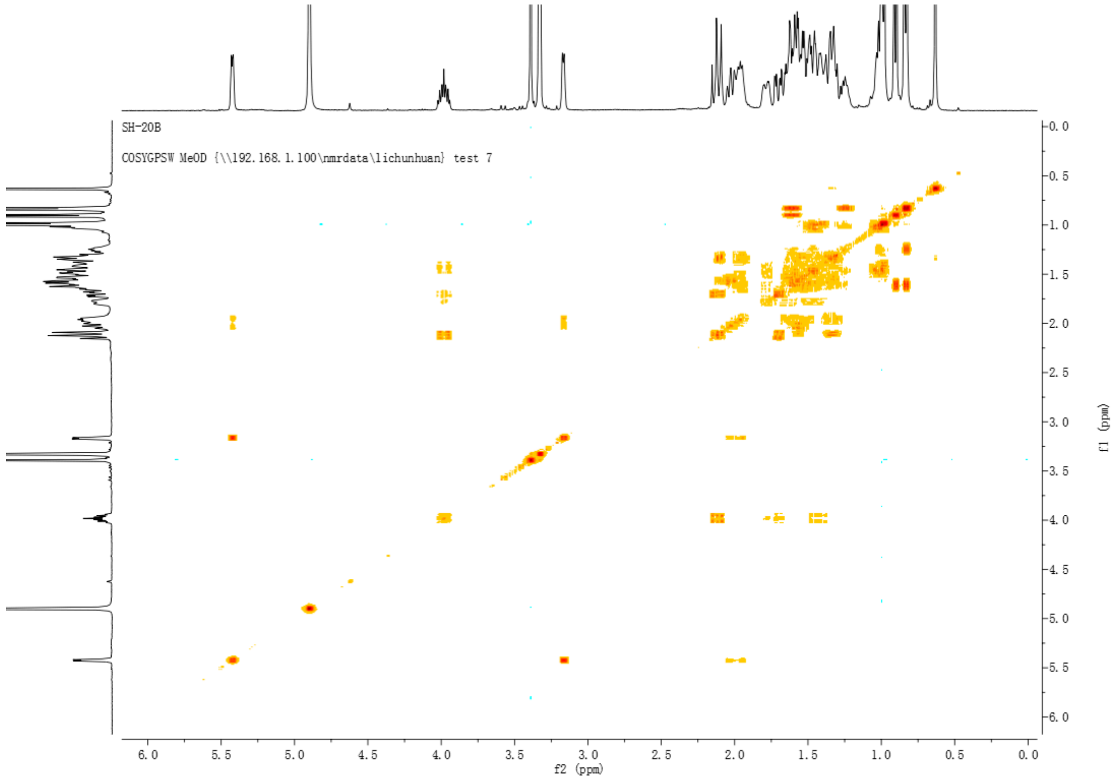

Figure S24. ^{1}H-^{1}H COSY spectrum of compound **4**

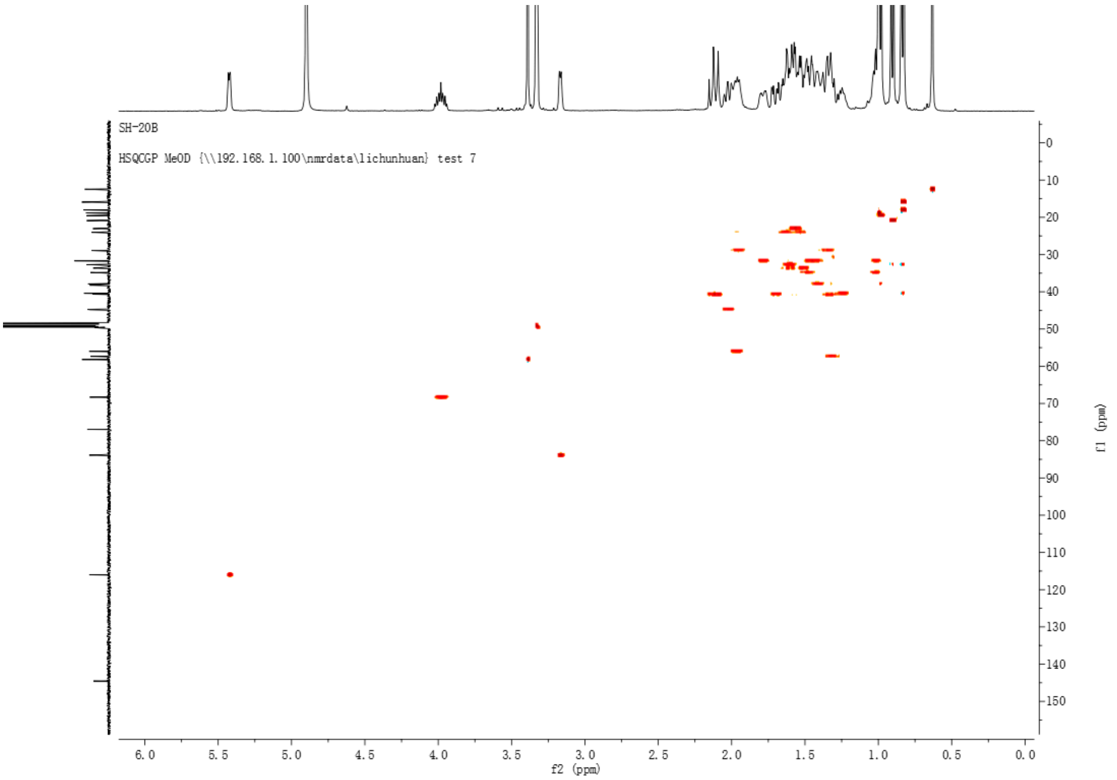

Figure S25. HSQC spectrum of compound **4**

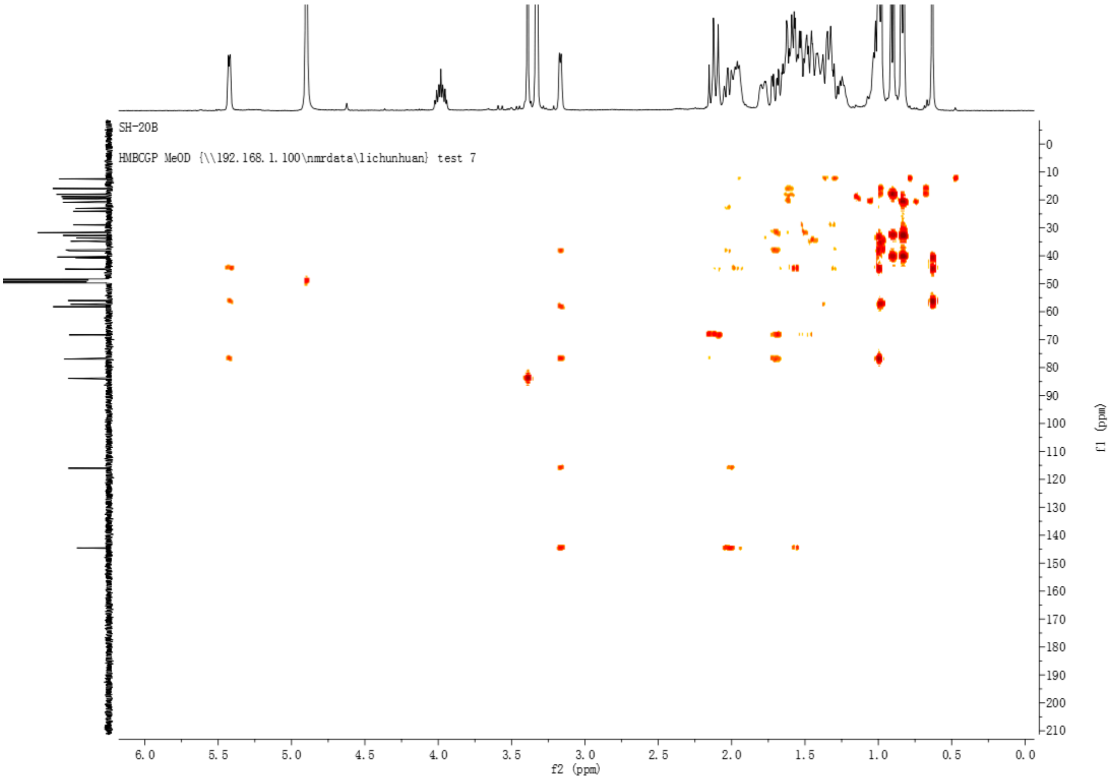

Figure S26. HMBC spectrum of compound **4**

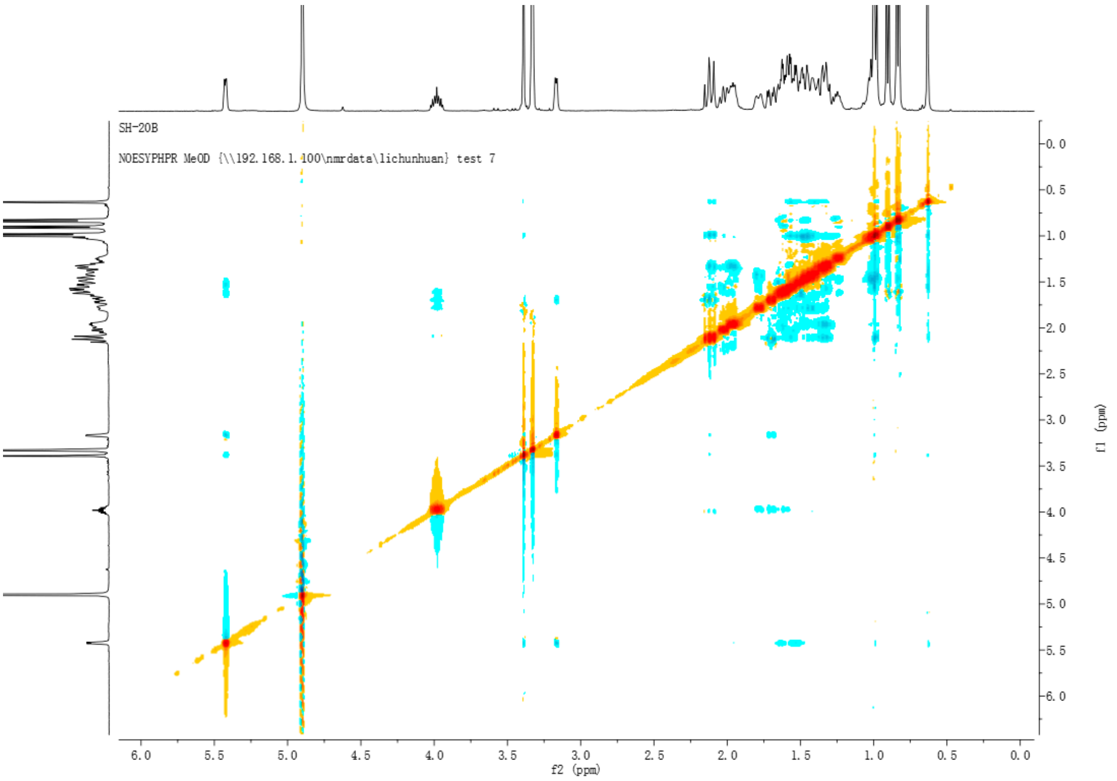

Figure S27. NOESY spectrum of compound **4**

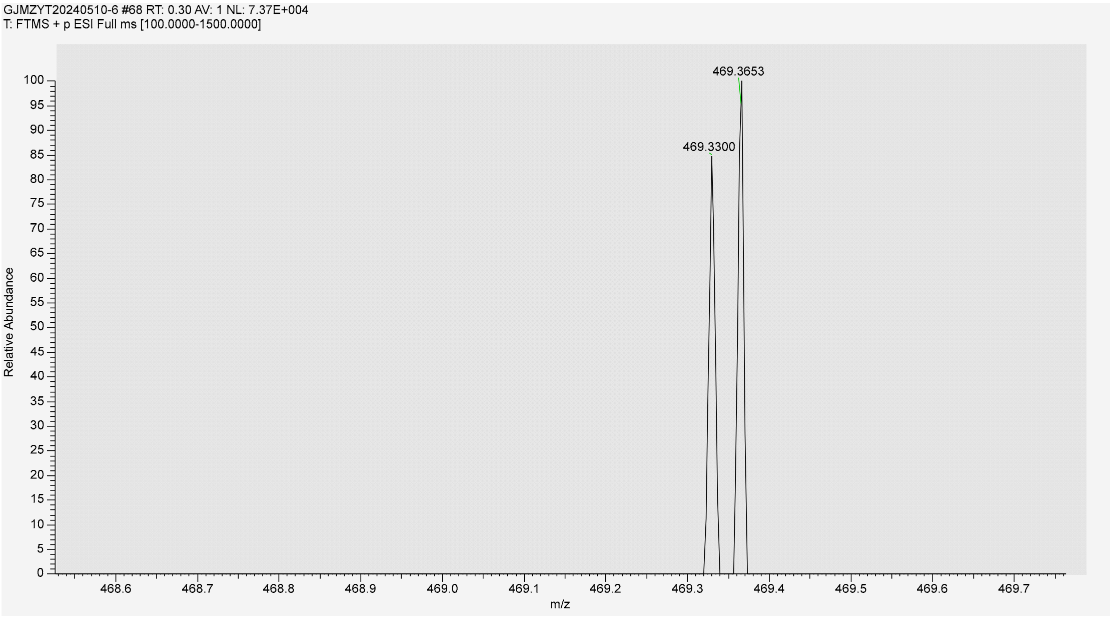

Figure S28. HRESIMS spectrum of compound **4**

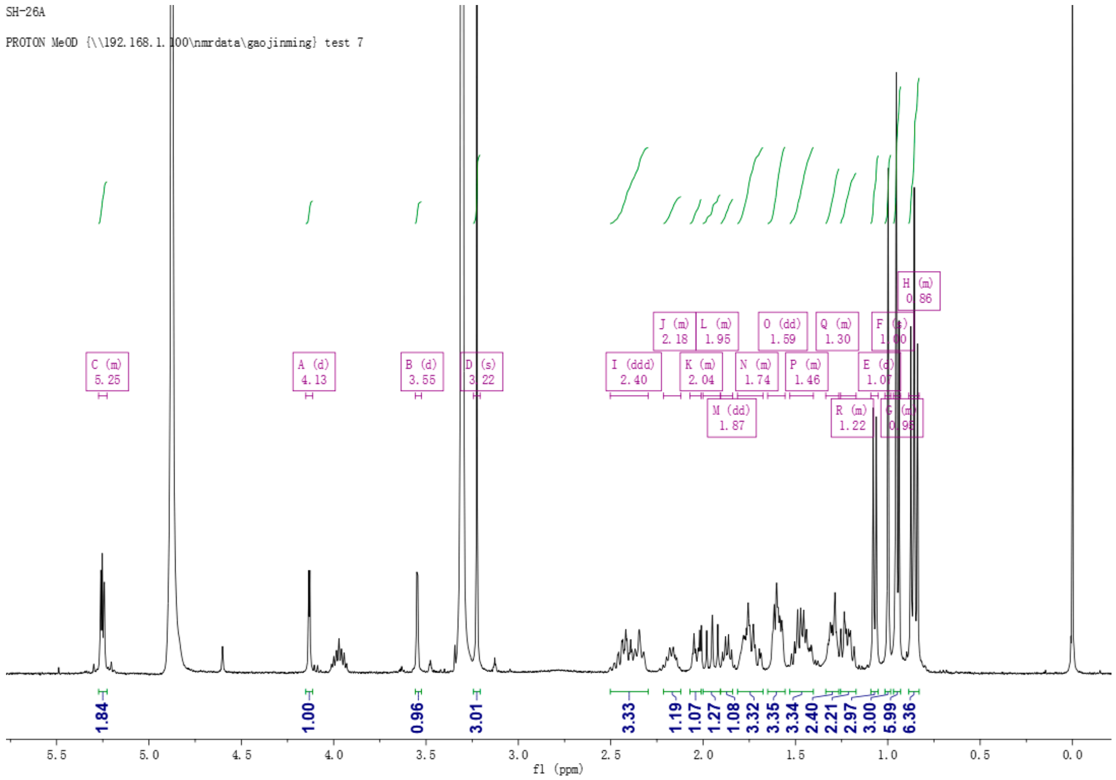

Figure S29. ^{1}H NMR spectrum of compound **5** (400MHz, methanol-d_4)

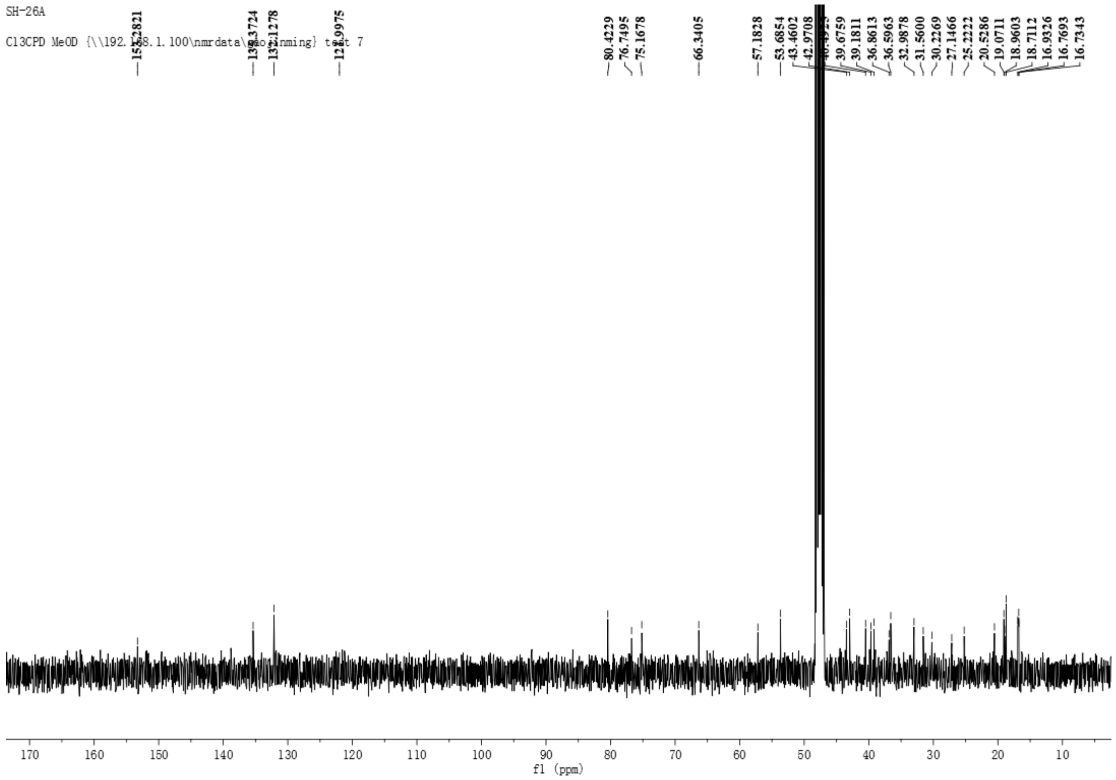

Figure S30. ^{13}C NMR spectrum of compound **5** (400MHz, methanol-d_4)

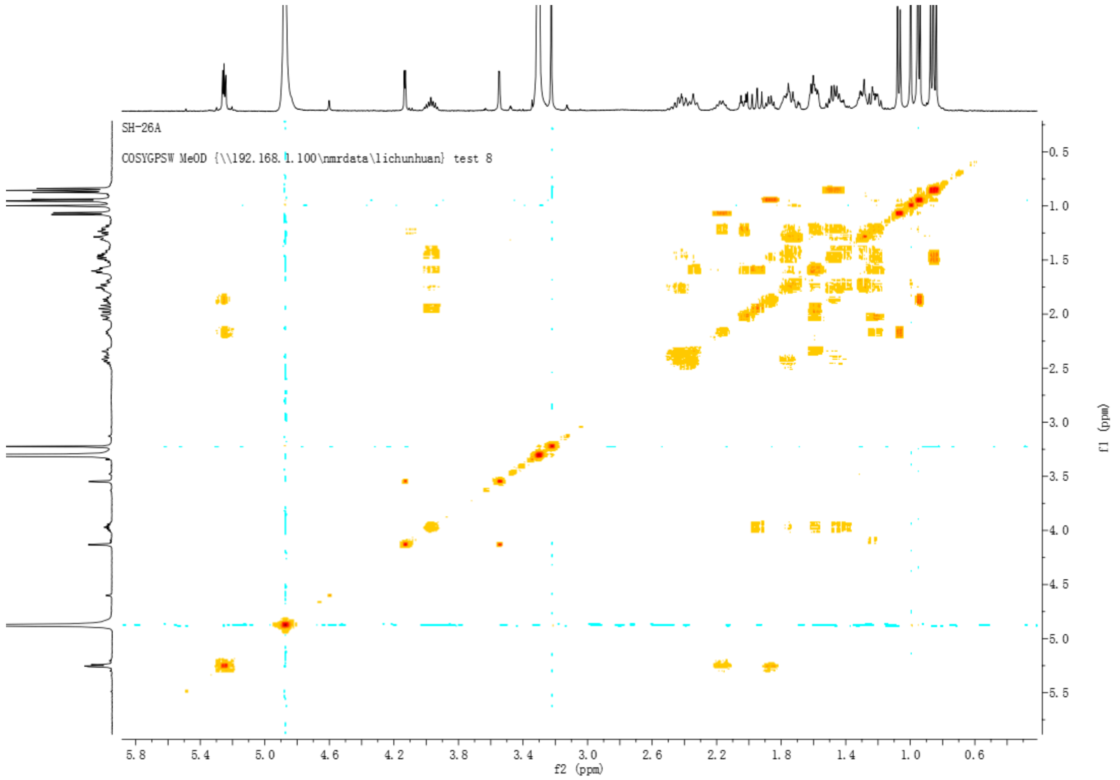

Figure S31. ^{1}H-^{1}H COSY spectrum of compound **5**

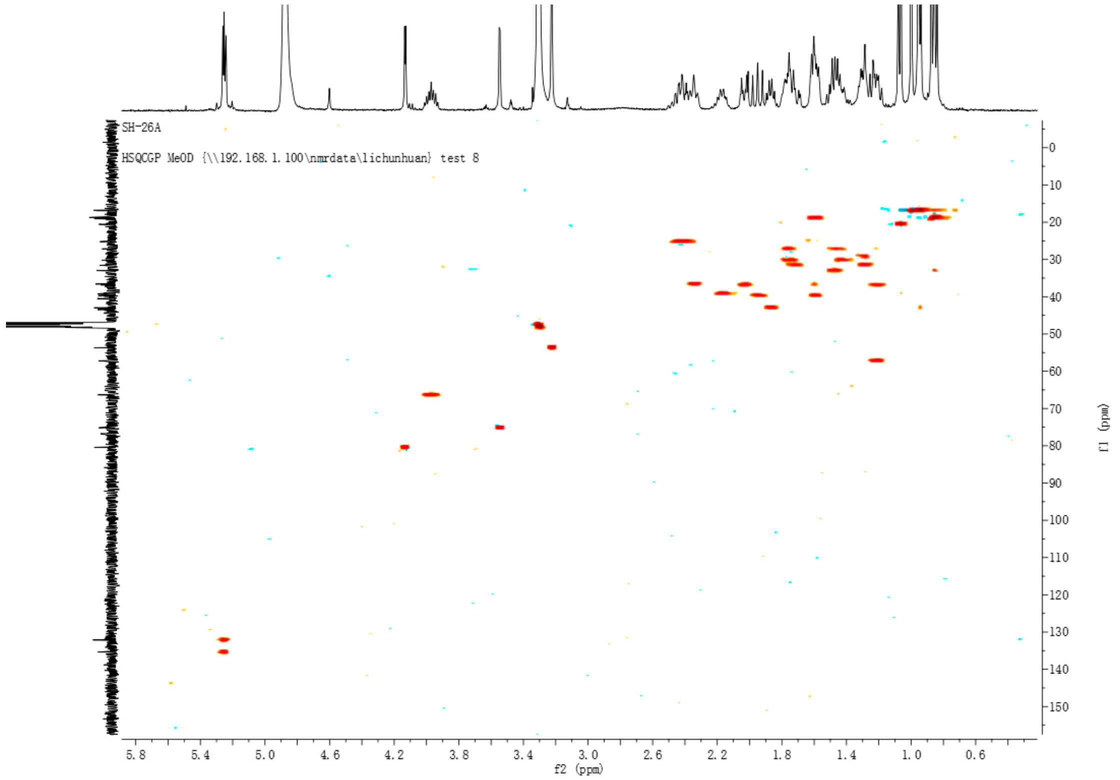

Figure S32. HSQC spectrum of compound **5**

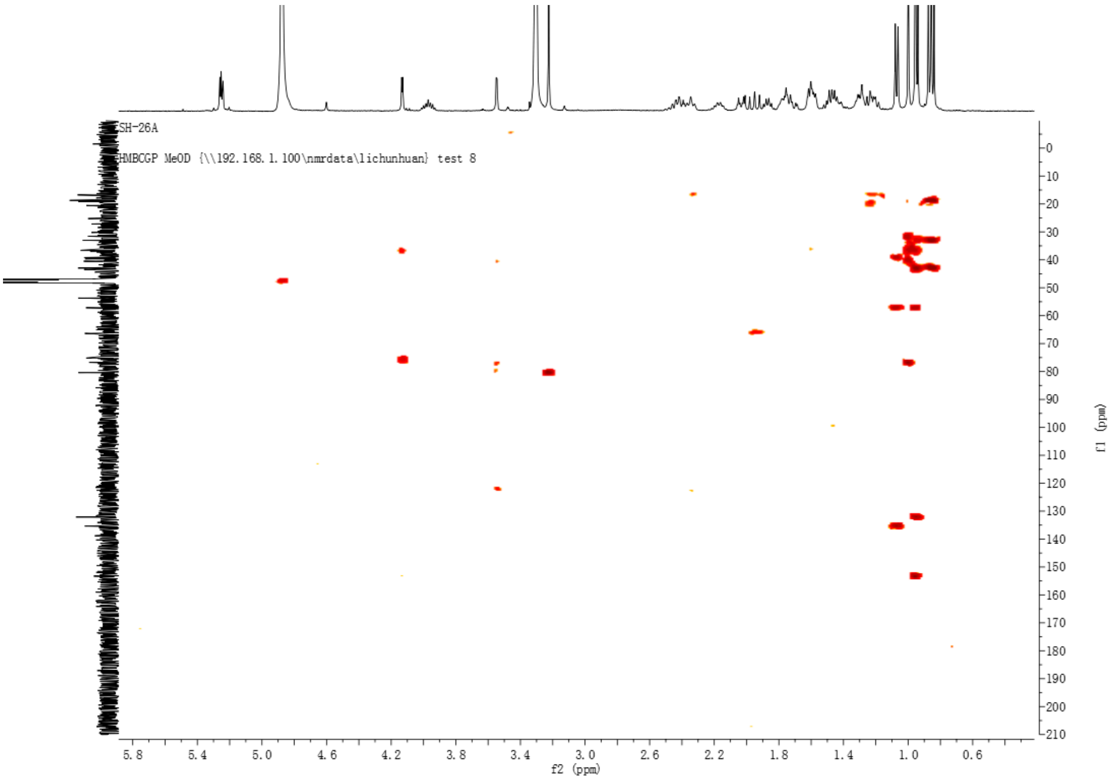

Figure S33. HMBC spectrum of compound **5**

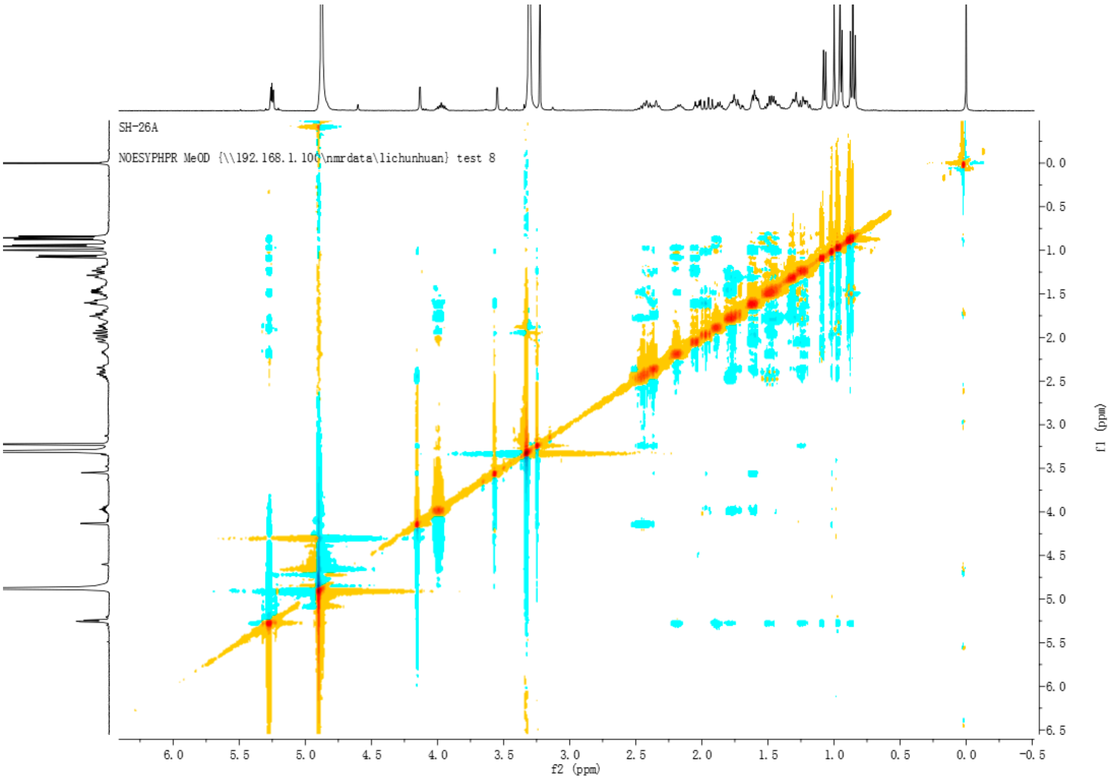

Figure S34. NOESY spectrum of compound **5**

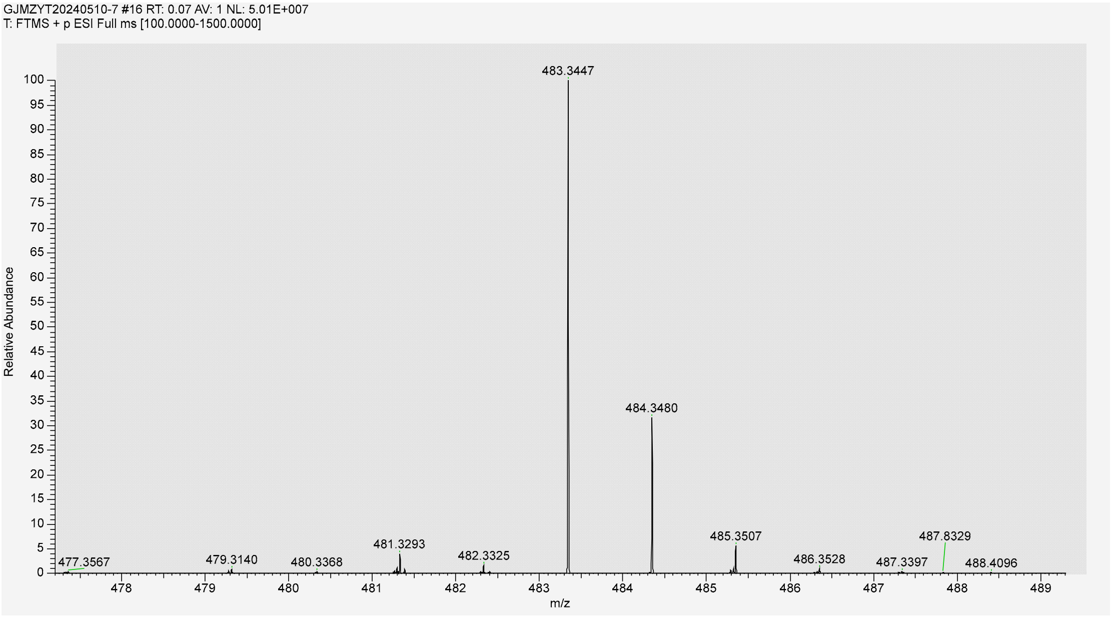

Figure S35. HRESIMS spectrum of compound **5**

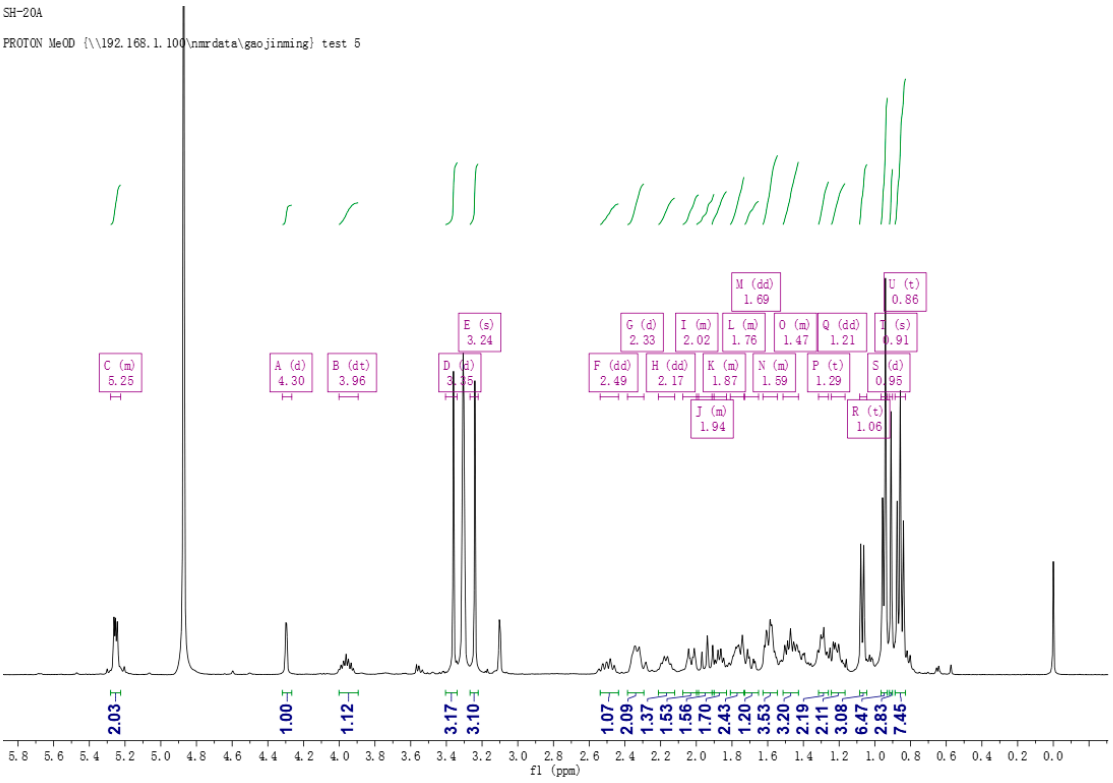

Figure S36. ^{1}H NMR spectrum of compound **6** (400MHz, methanol-d_4)

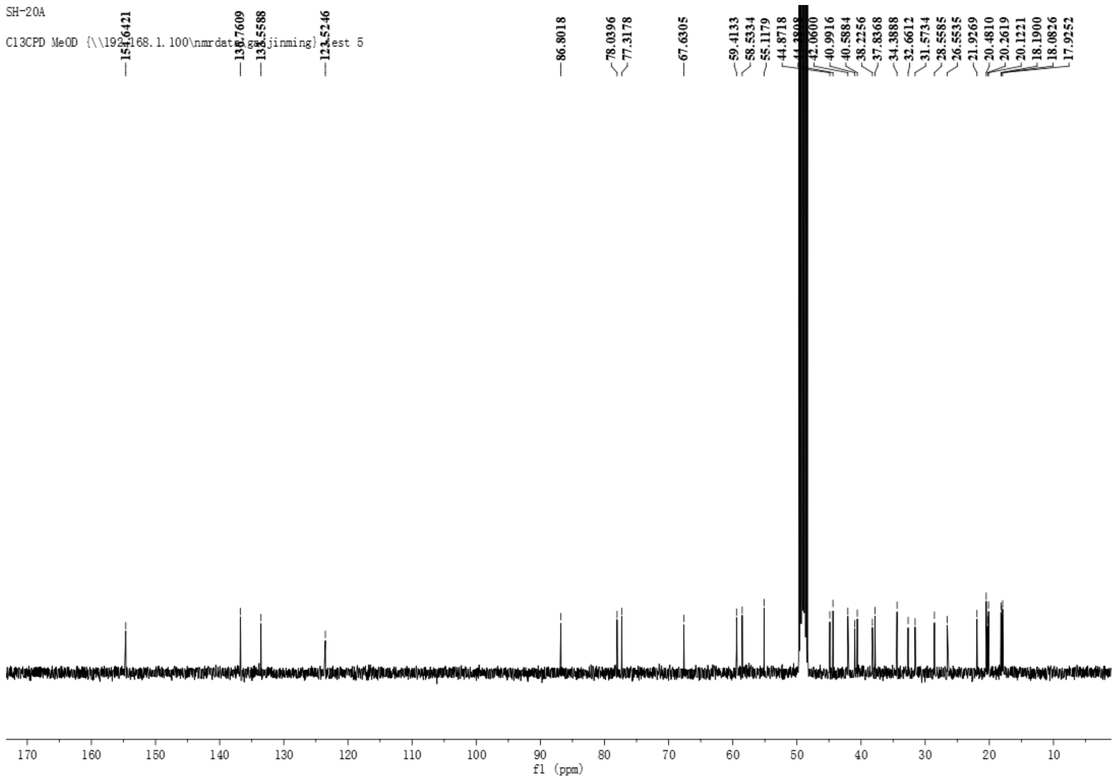

Figure S37. ^{13}C NMR spectrum of compound **6** (100MHz, methanol-*d*$_4$)

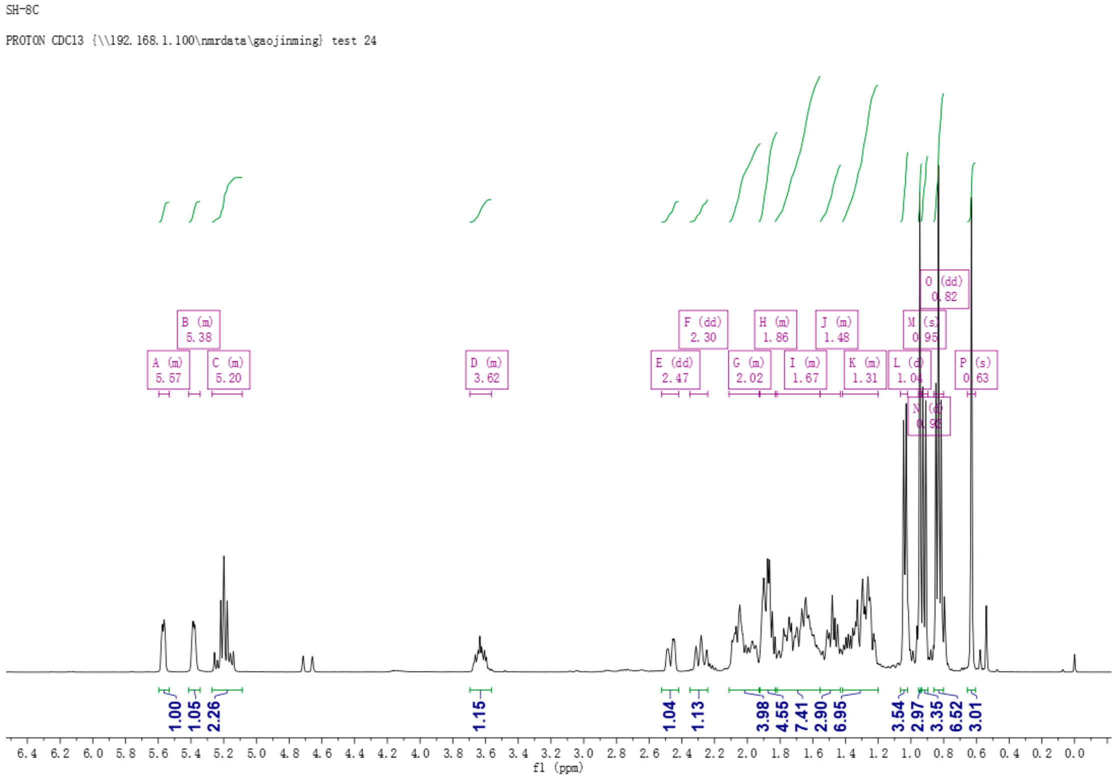

Figure S38. ^{1}H NMR spectrum of compound **7** (400MHz, $CDCl_3$)

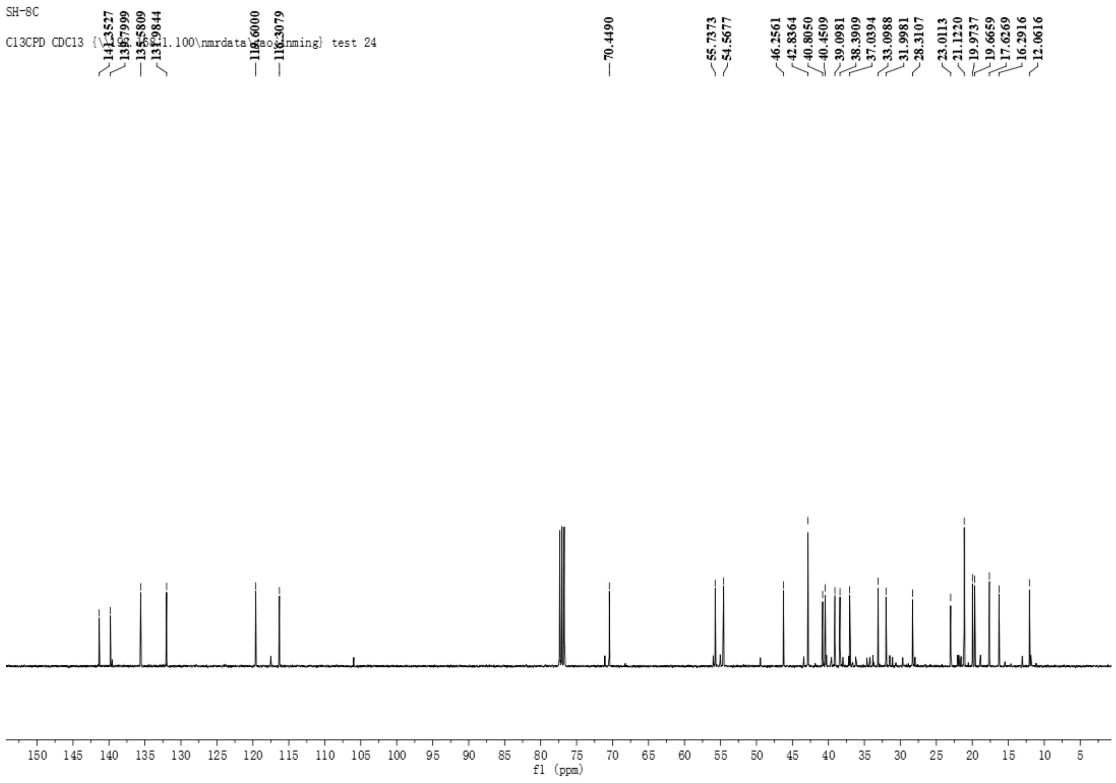

Figure S39. ^{13}C NMR spectrum of compound **7** (100MHz, $CDCl_3$)

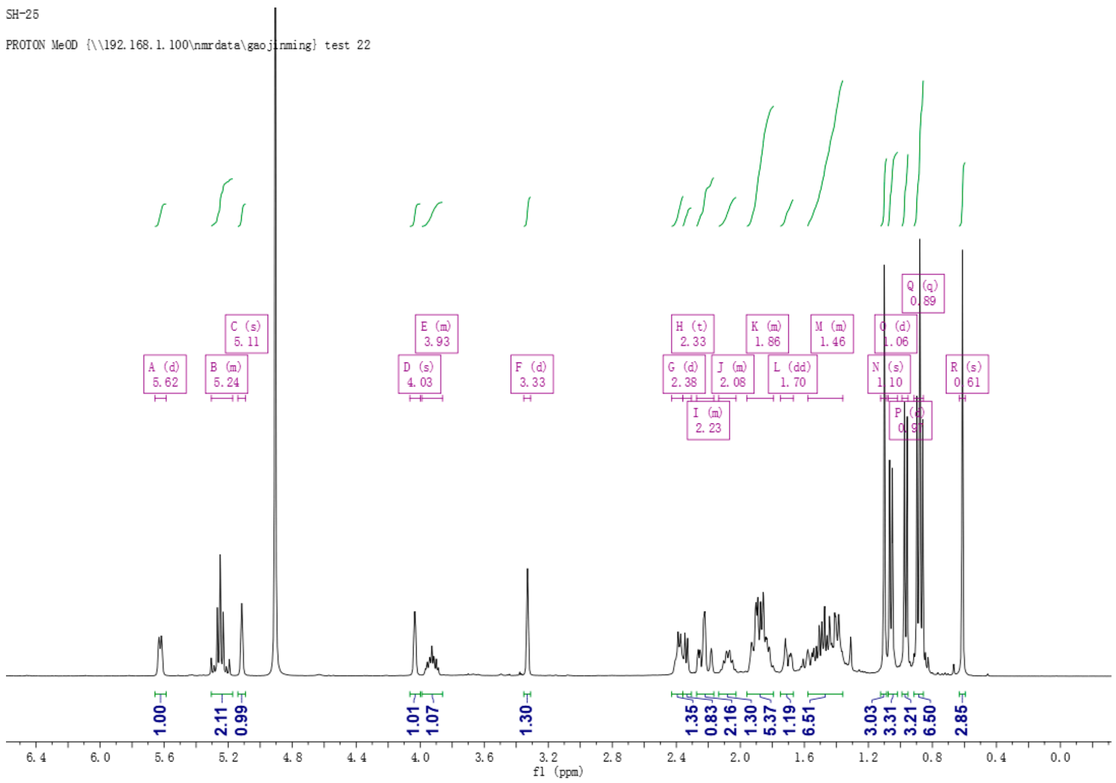

Figure S40. ^{1}H NMR spectrum of compound **8** (400MHz, methanol-d_4)

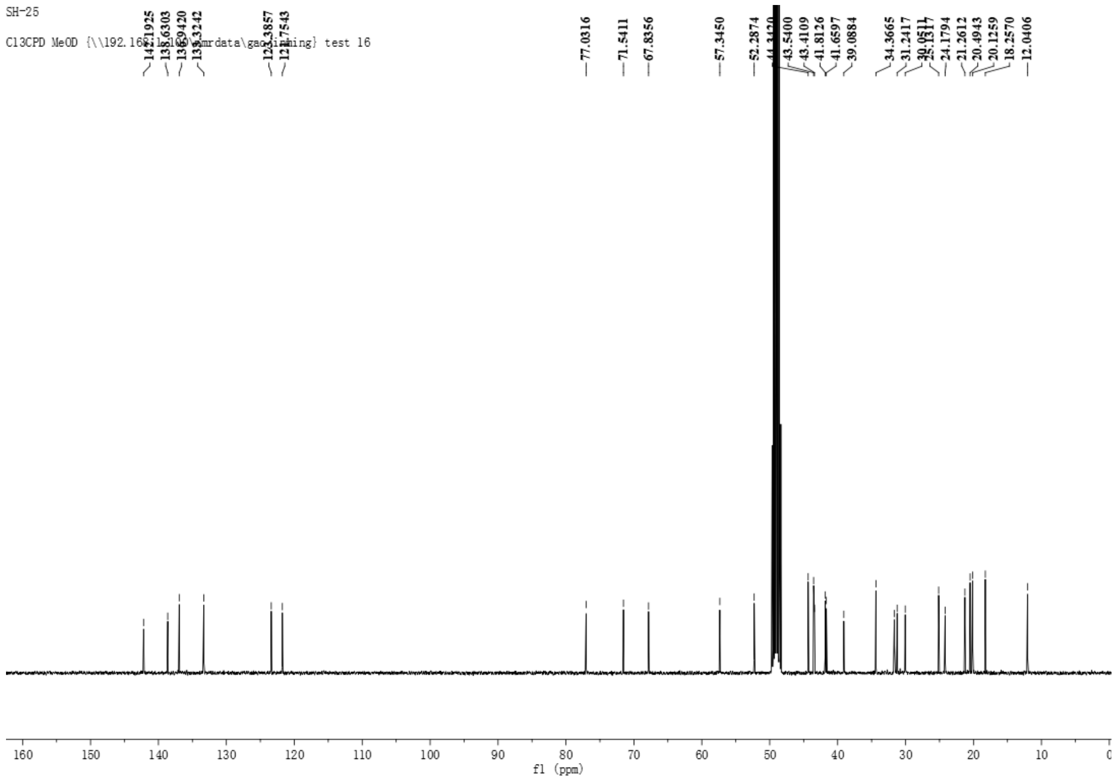

Figure S41. ^{13}C NMR spectrum of compound **8** (100MHz, methanol-d_4)

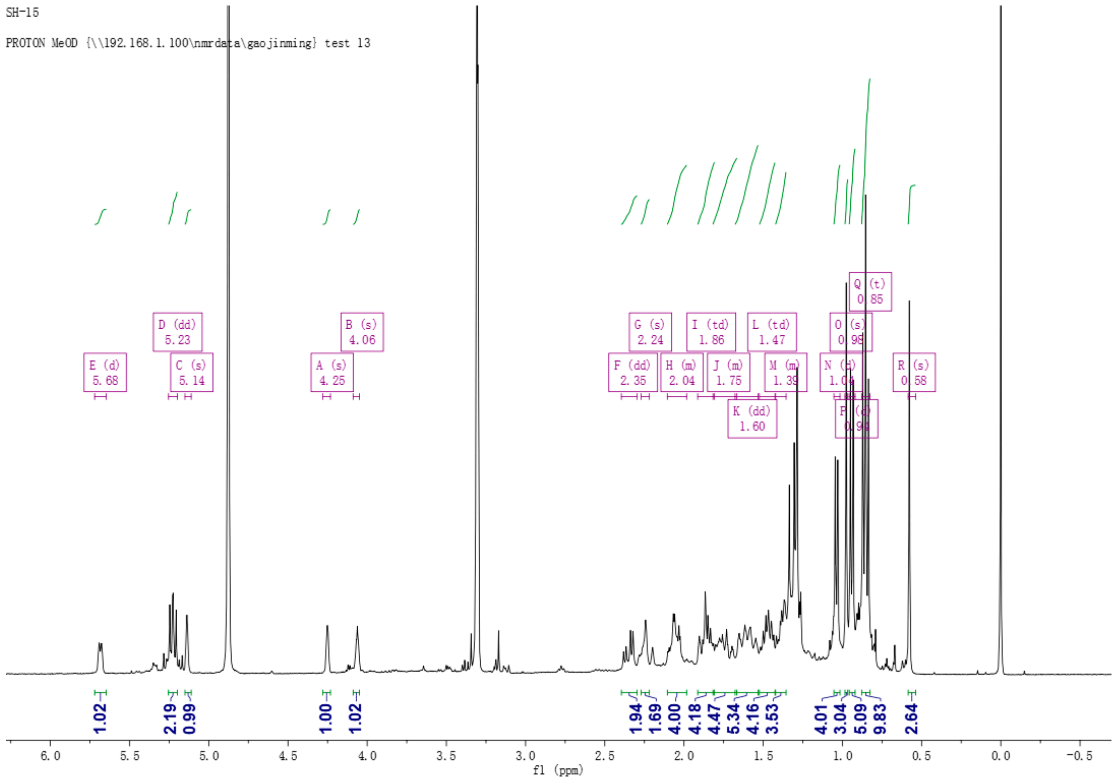

Figure S42. ^{1}H NMR spectrum of compound **9** (400MHz, methanol-d_4)

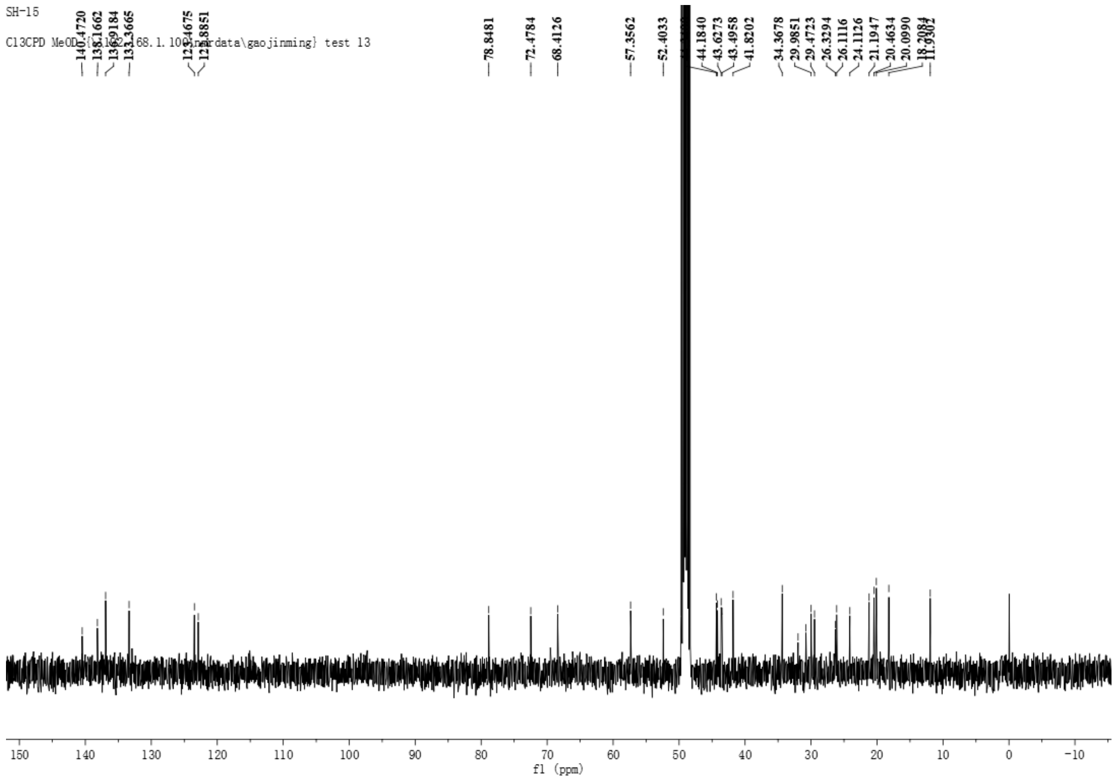

Figure S43. ^{13}C NMR spectrum of compound **9** (100MHz, methanol-*d*4)

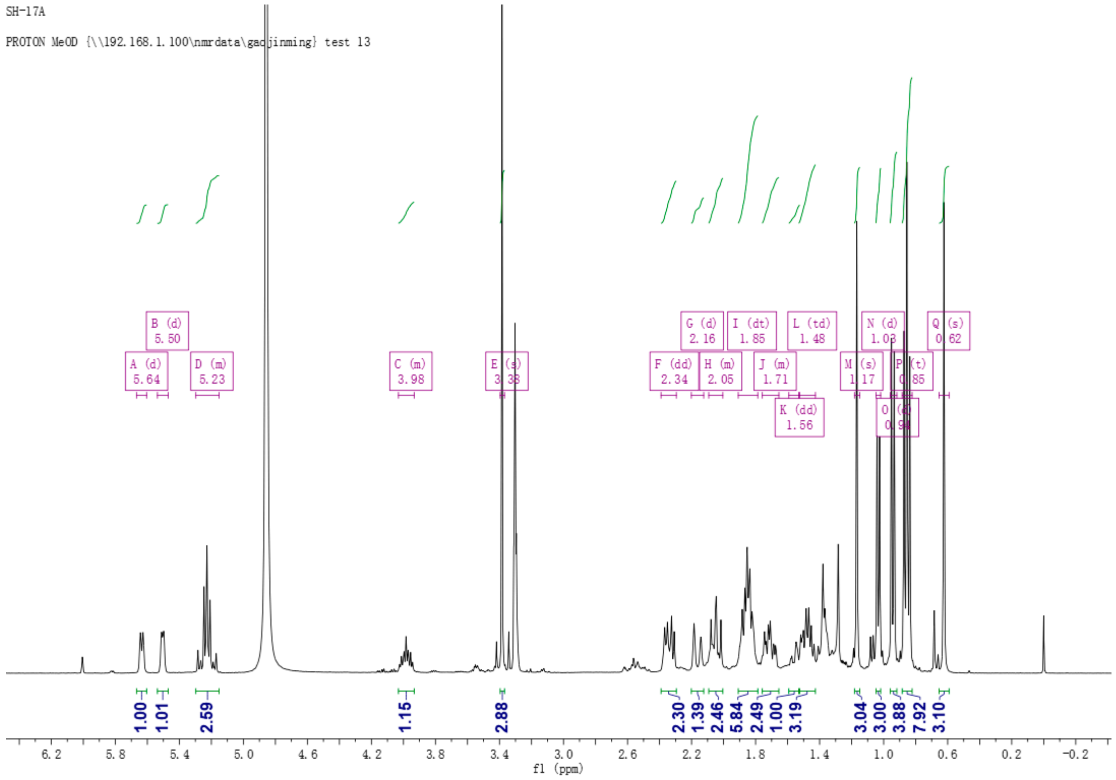

Figure S44. ^{1}H NMR spectrum of compound **10** (400MHz, methanol-*d*4)

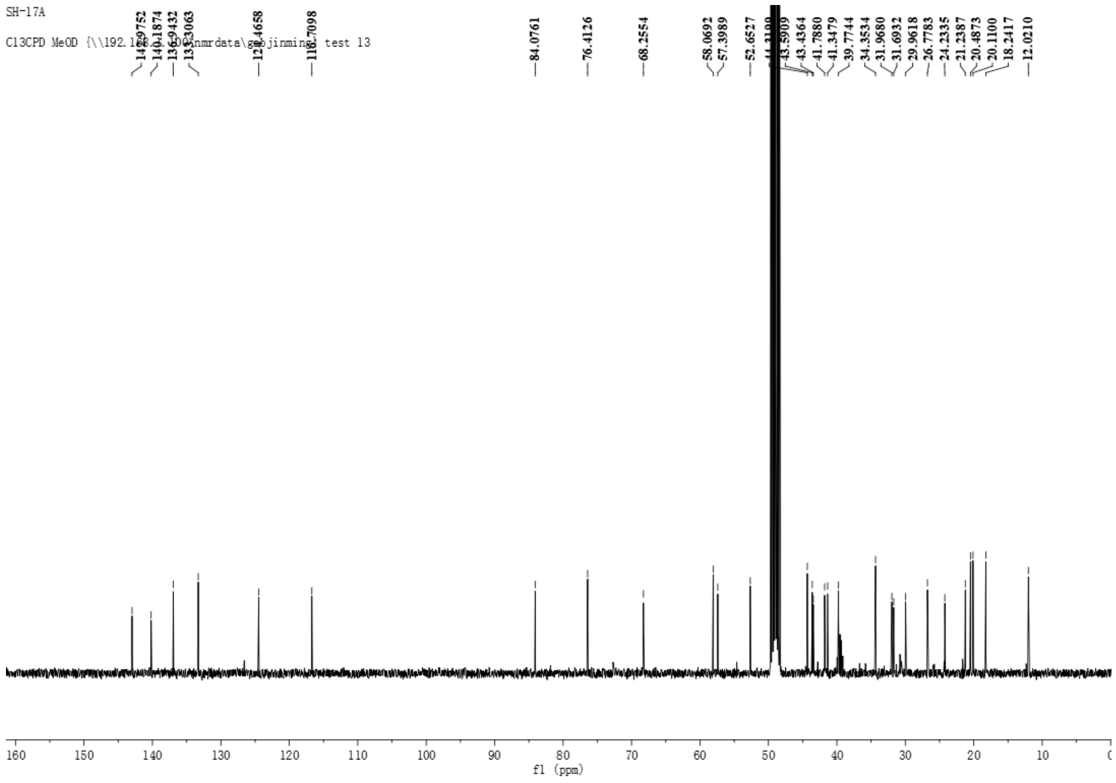

Figure S45. ^{13}C NMR spectrum of compound **10** (100MHz, methanol-d_4)

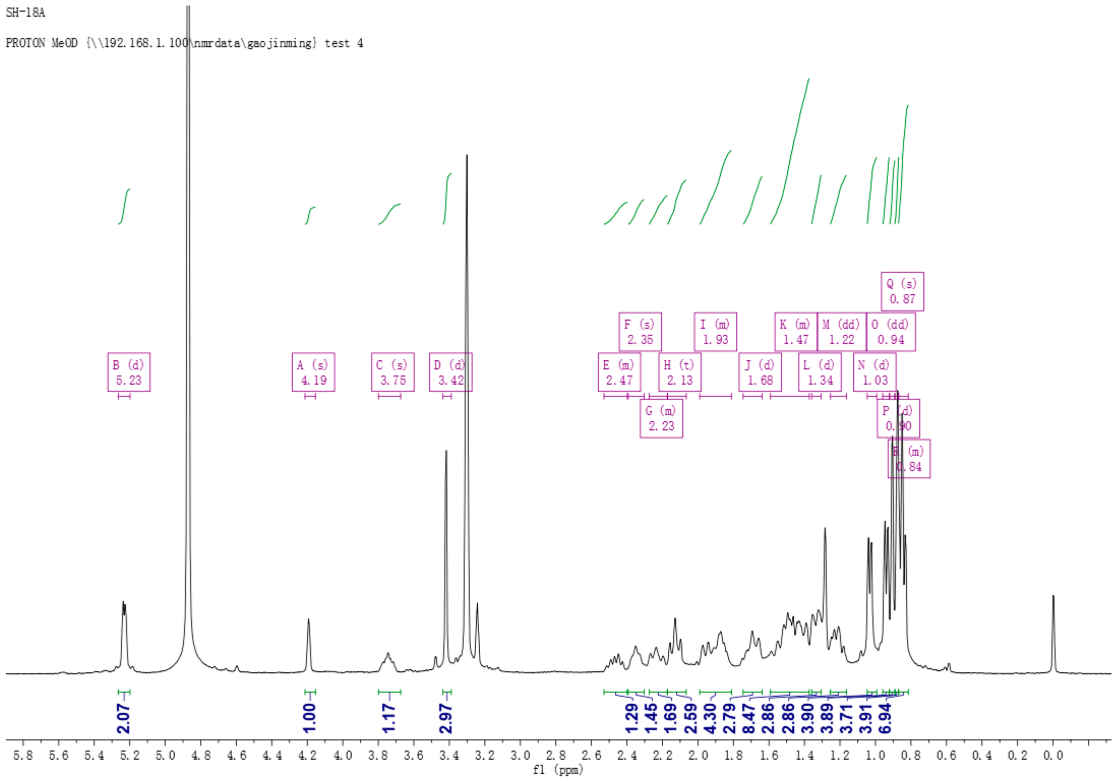

Figure S46. ^{1}H NMR spectrum of compound **11** (400MHz, methanol-d_4)

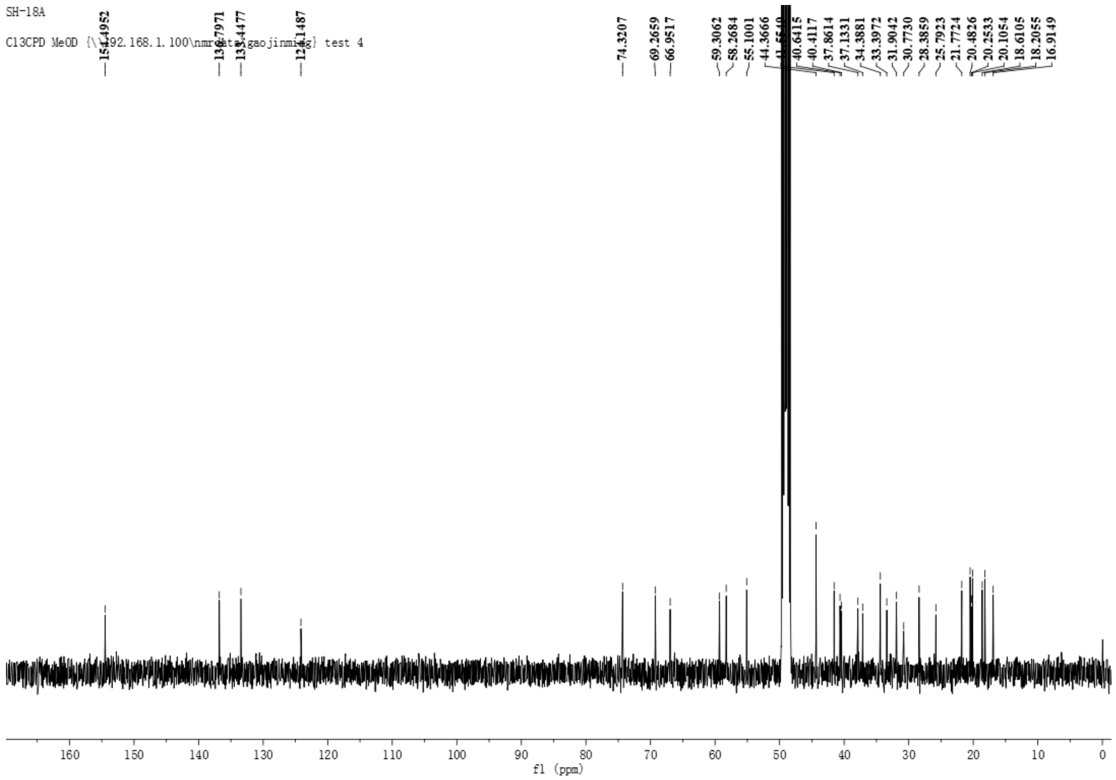

Figure S47. ^{13}C NMR spectrum of compound **11** (100MHz, methanol-d_4)

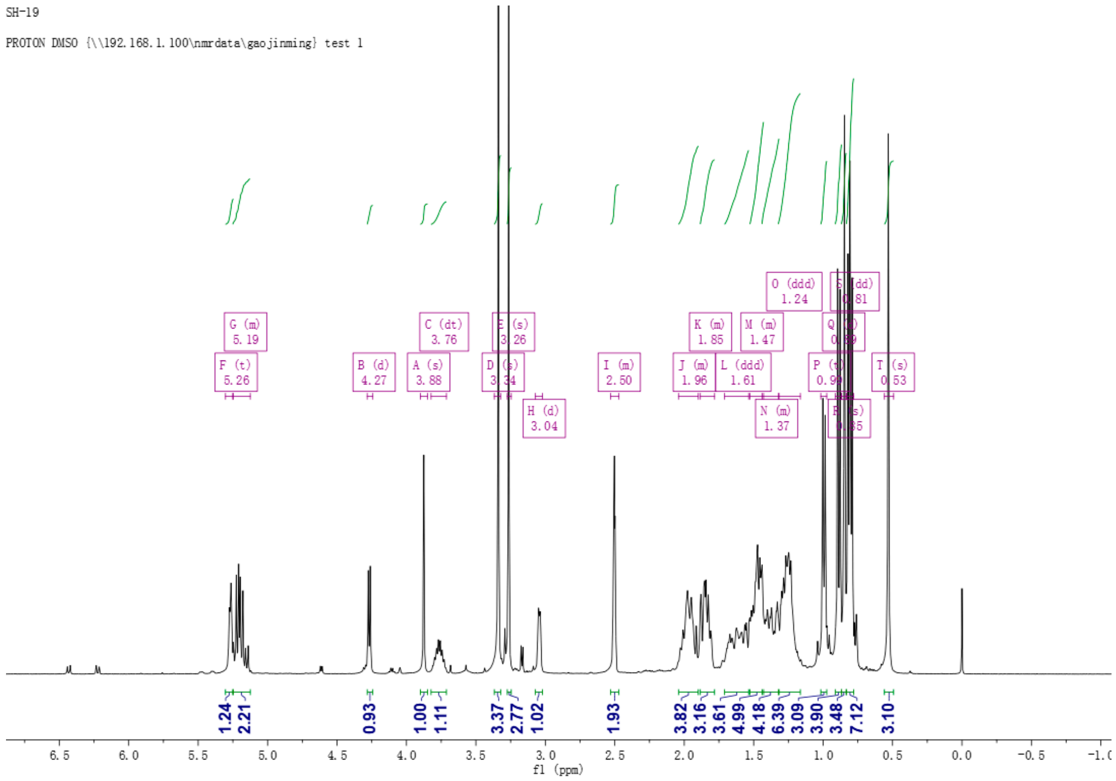

Figure S48. ^{1}H NMR spectrum of compound **12** (400MHz, DMSO-d_6)

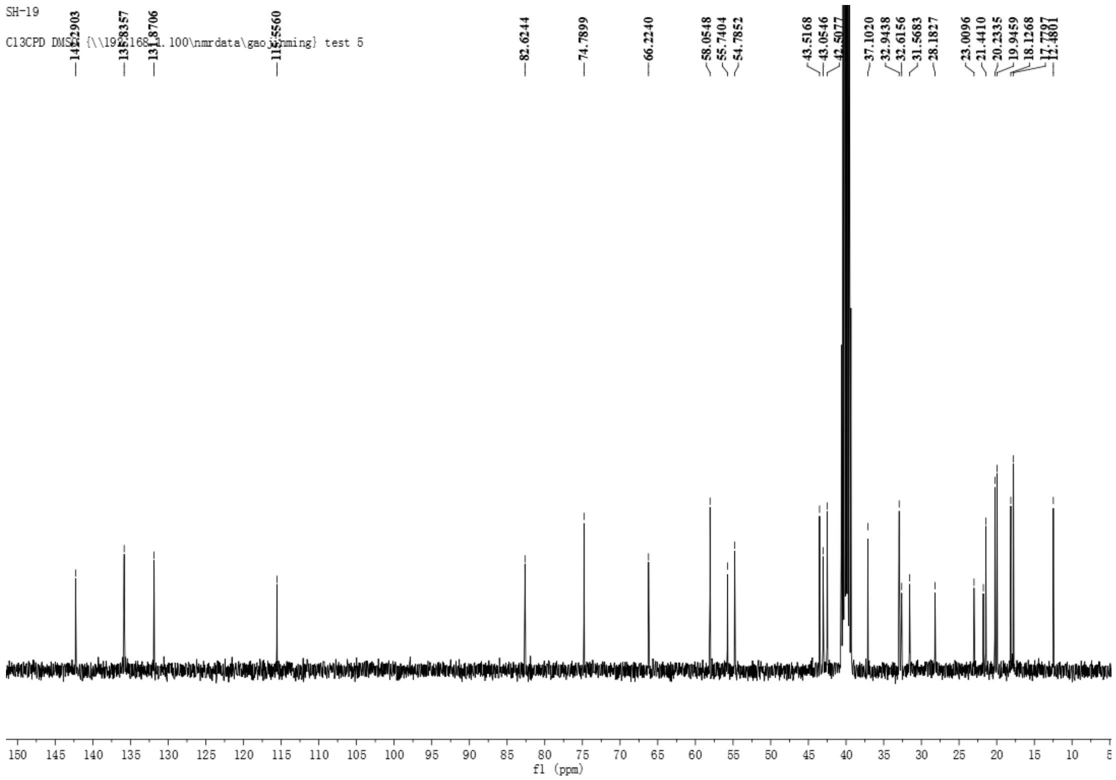

Figure S49. ^{13}C NMR spectrum of compound **12** (100MHz, DMSO-d_6)

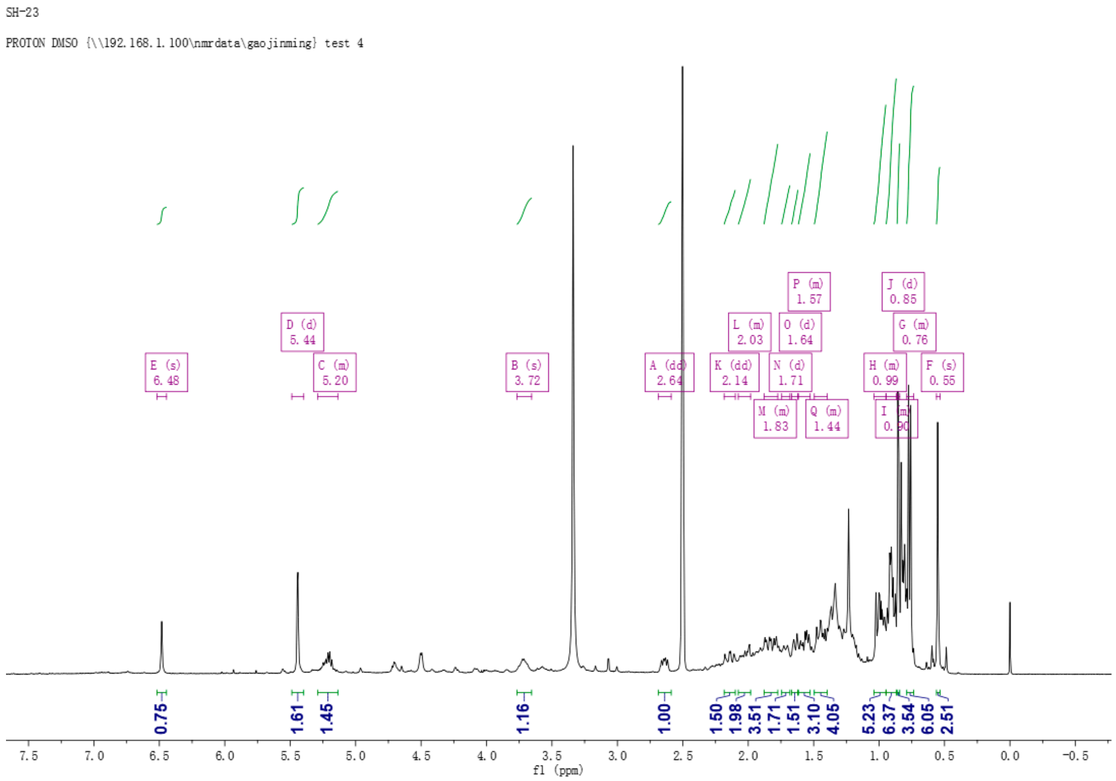

Figure S50. ^{1}H NMR spectrum of compound **13** (400MHz, DMSO-d_6)

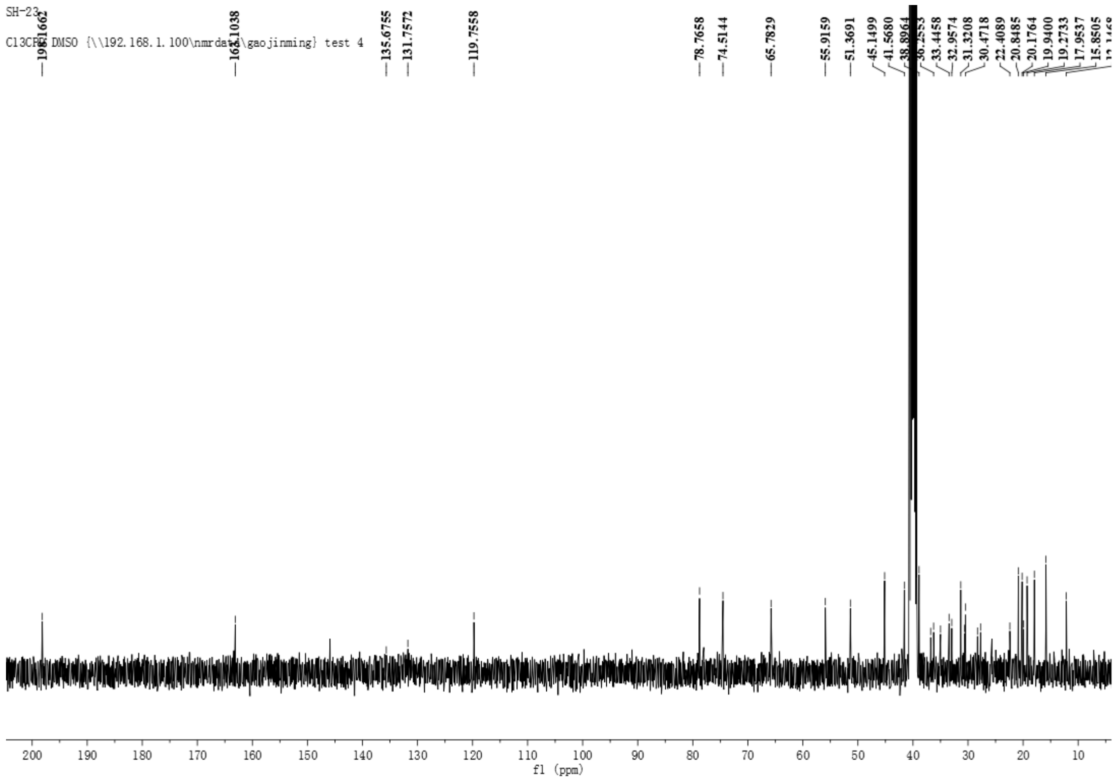

Figure S51. ^{13}C NMR spectrum of compound **13** (100MHz, DMSO-d_6)

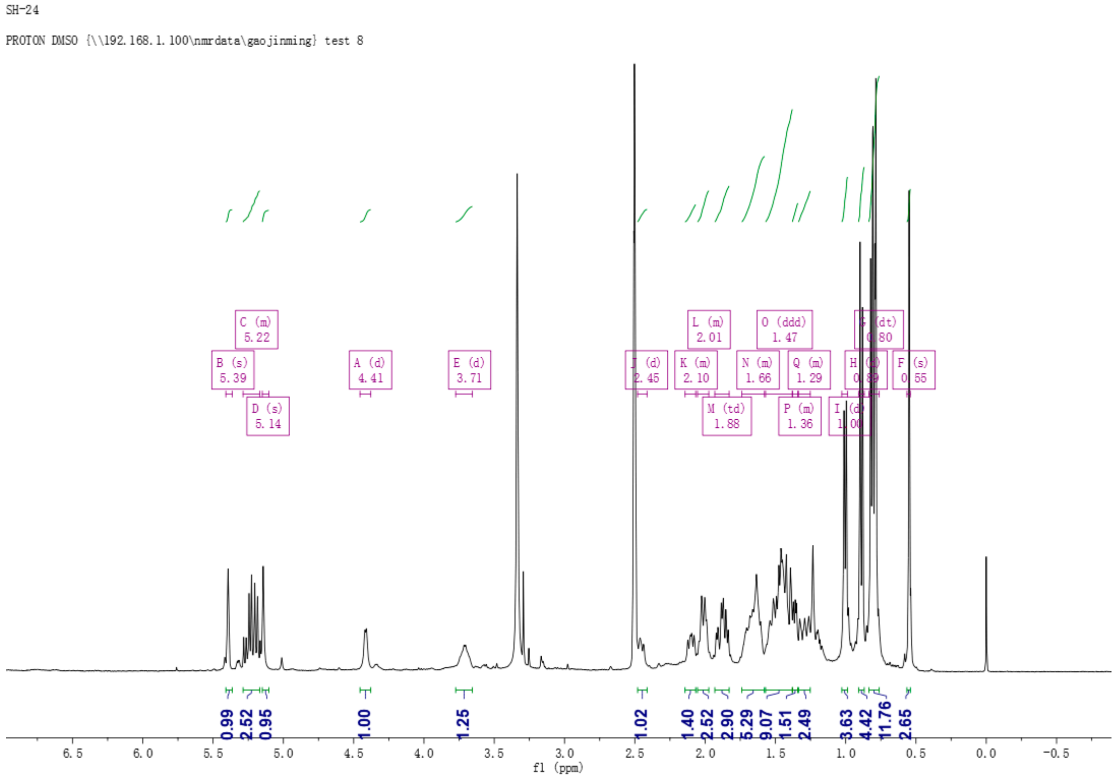

Figure S52. ^{1}H NMR spectrum of compound **14** (400MHz, DMSO-d_6)

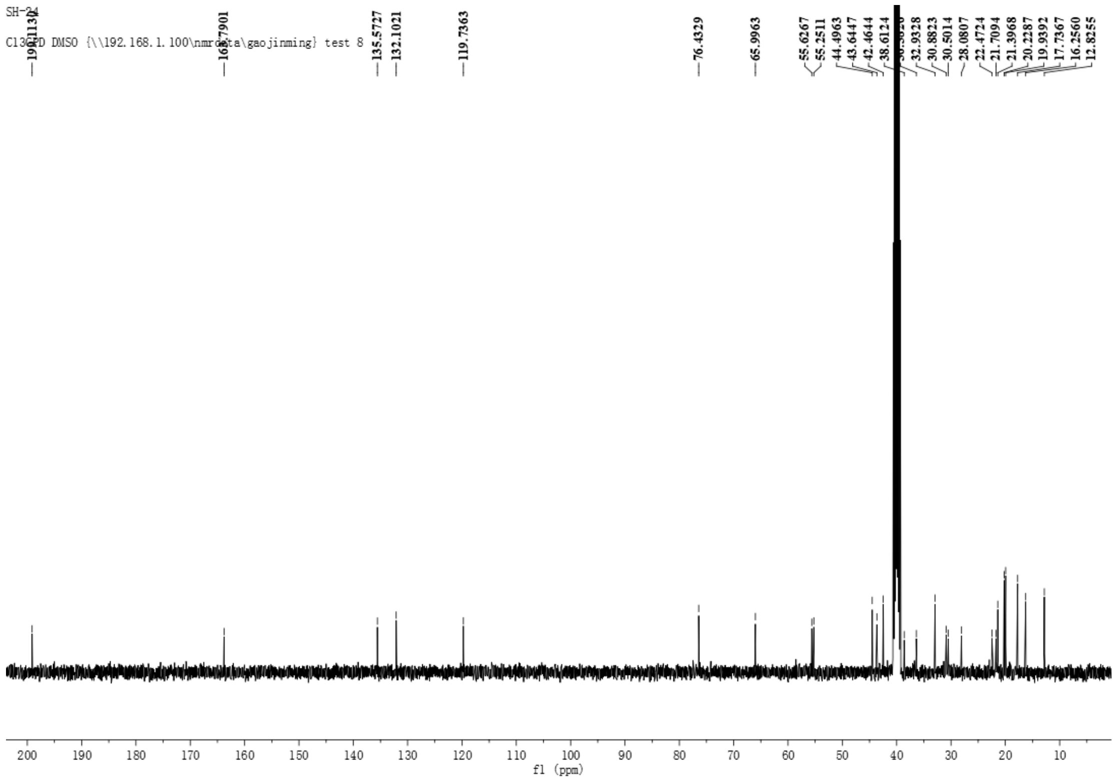

Figure S53. ^{13}C NMR spectrum of compound **14** (100MHz, DMSO-d_6)

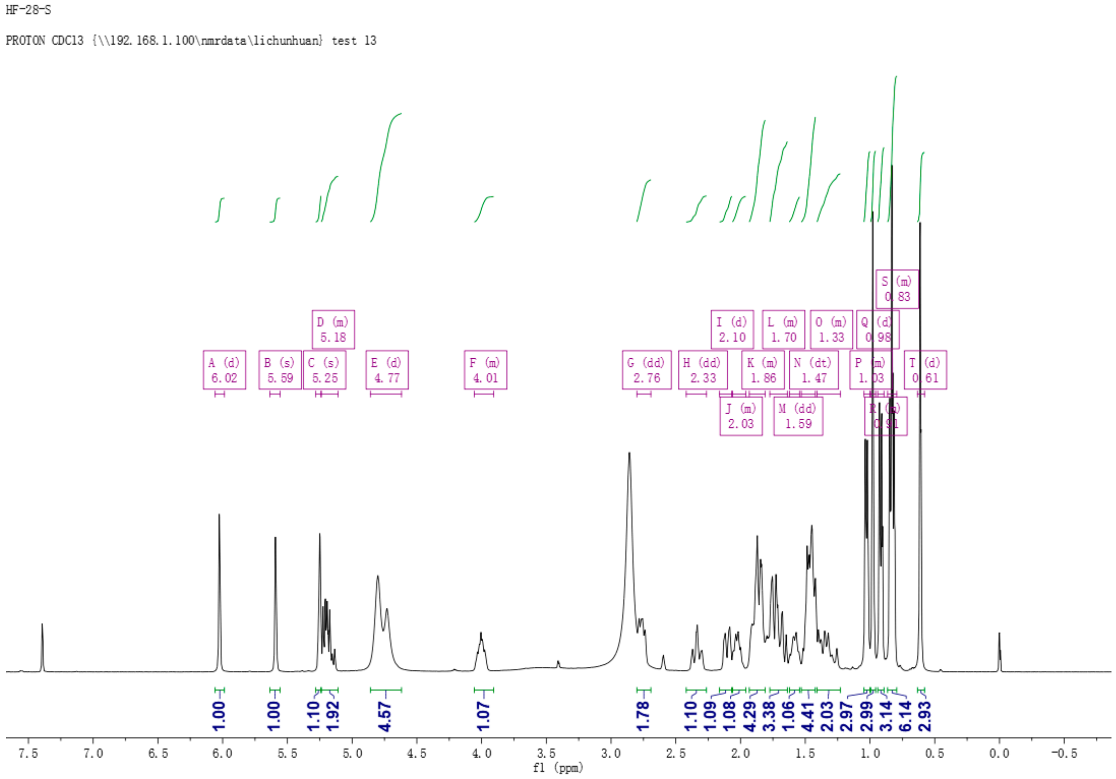

Figure S54. ^{1}H NMR spectrum of compound **15** (400MHz, $CDCl_3$)

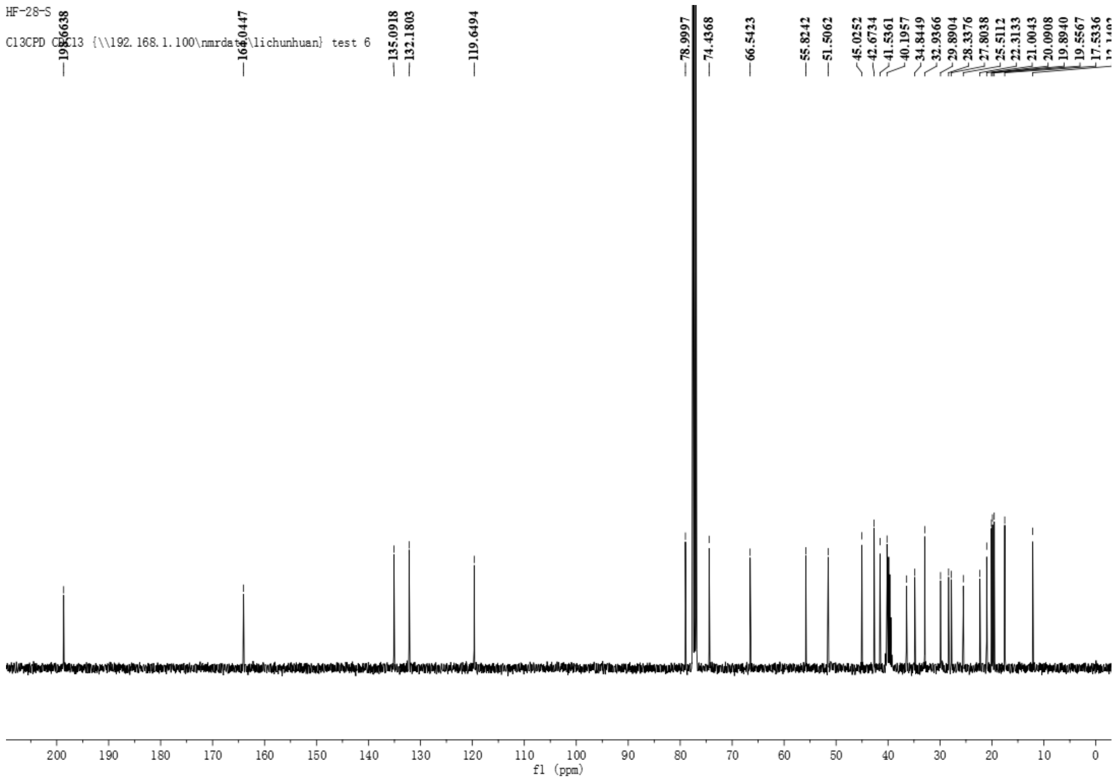

Figure S55. ^{13}C NMR spectrum of compound **15** (100MHz, $CDCl_3$)

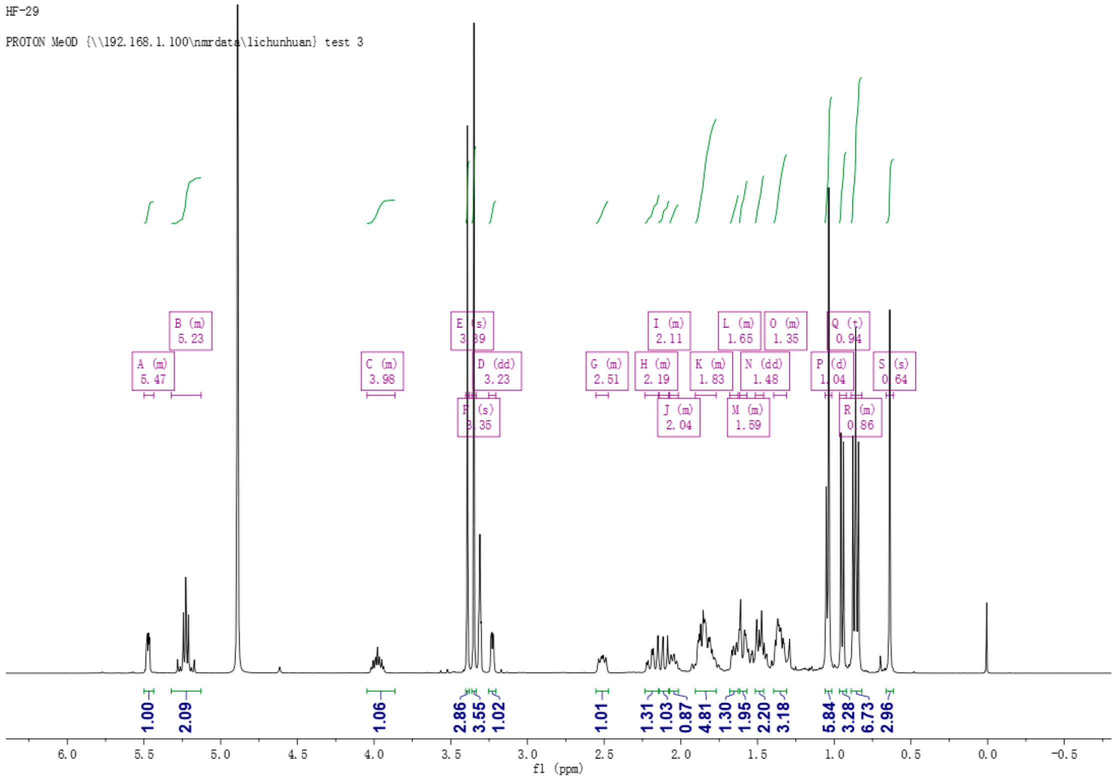

Figure S56. ^{1}H NMR spectrum of compound **16** (400MHz, methanol-d_4)

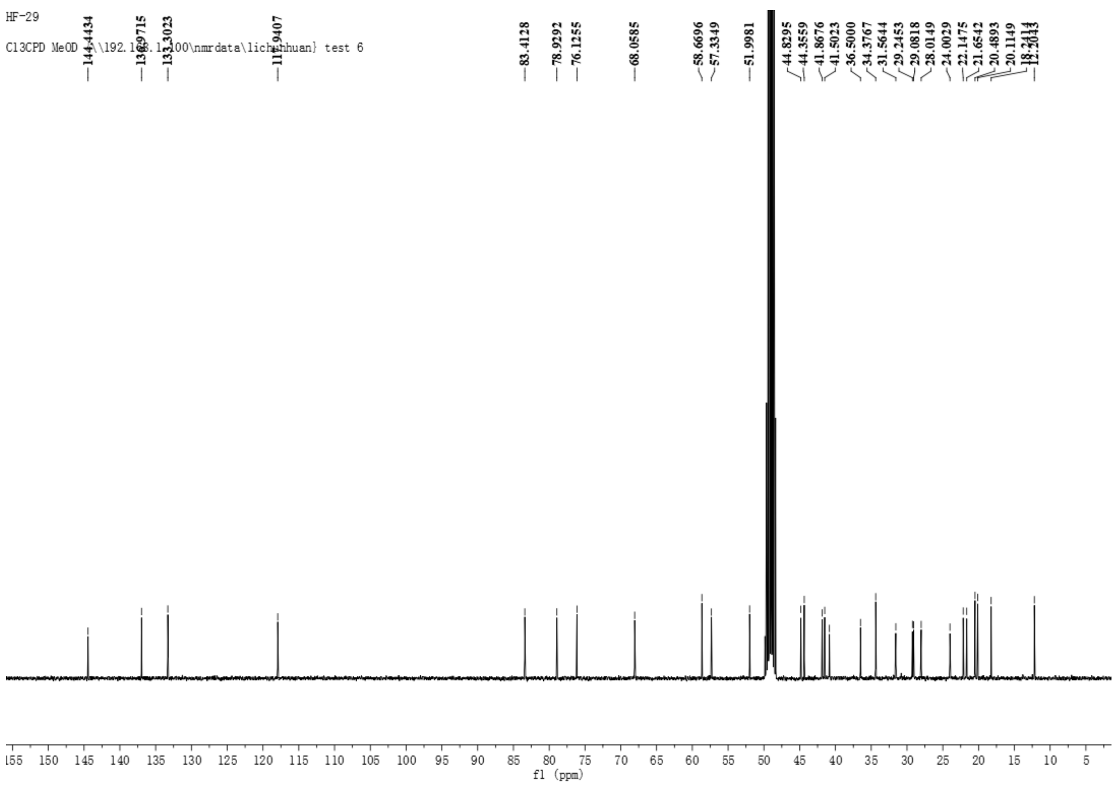

Figure S57. ^{13}C NMR spectrum of compound **16** (100MHz, methanol-d_4)

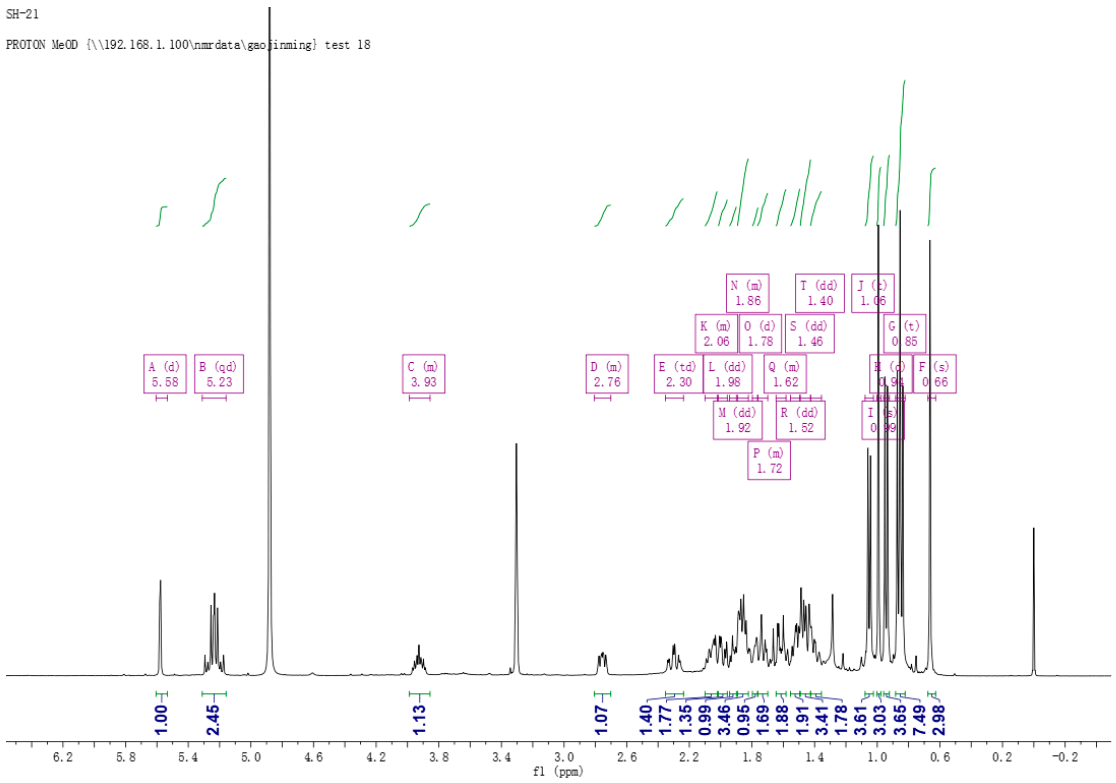

Figure S58. ^{1}H NMR spectrum of compound **17** (400MHz, methanol-d_4)

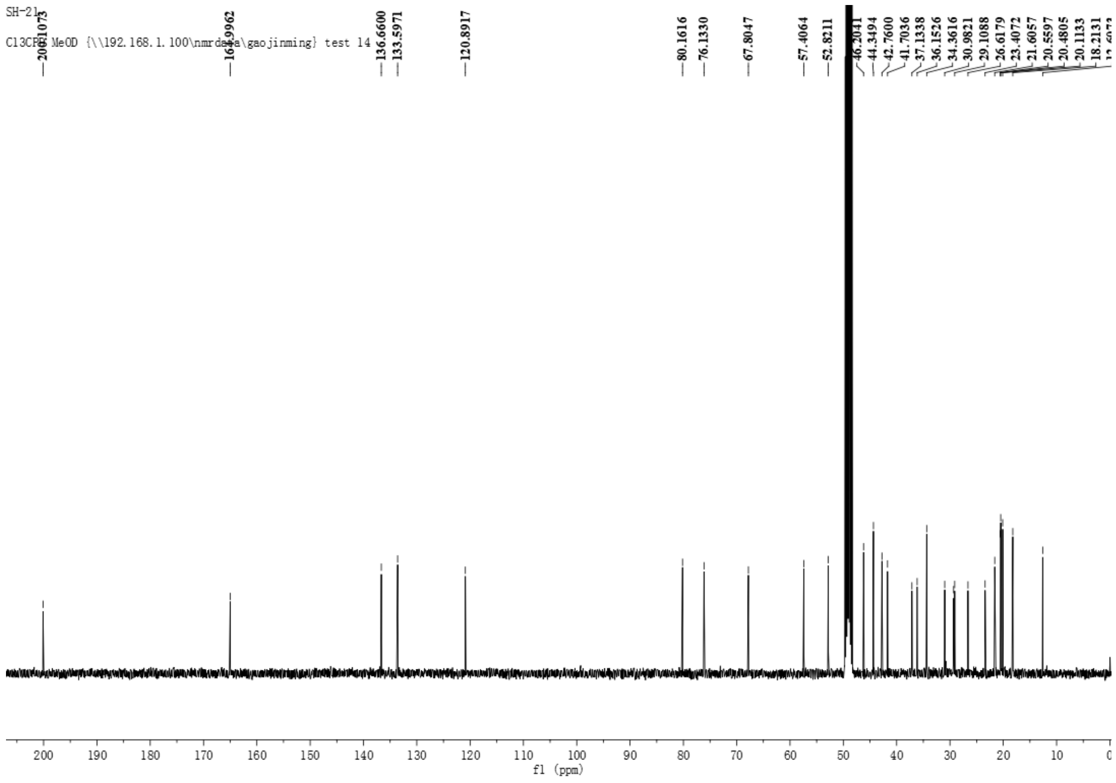

Figure S59. ^{13}C NMR spectrum of compound **17** (100MHz, methanol-d_4)

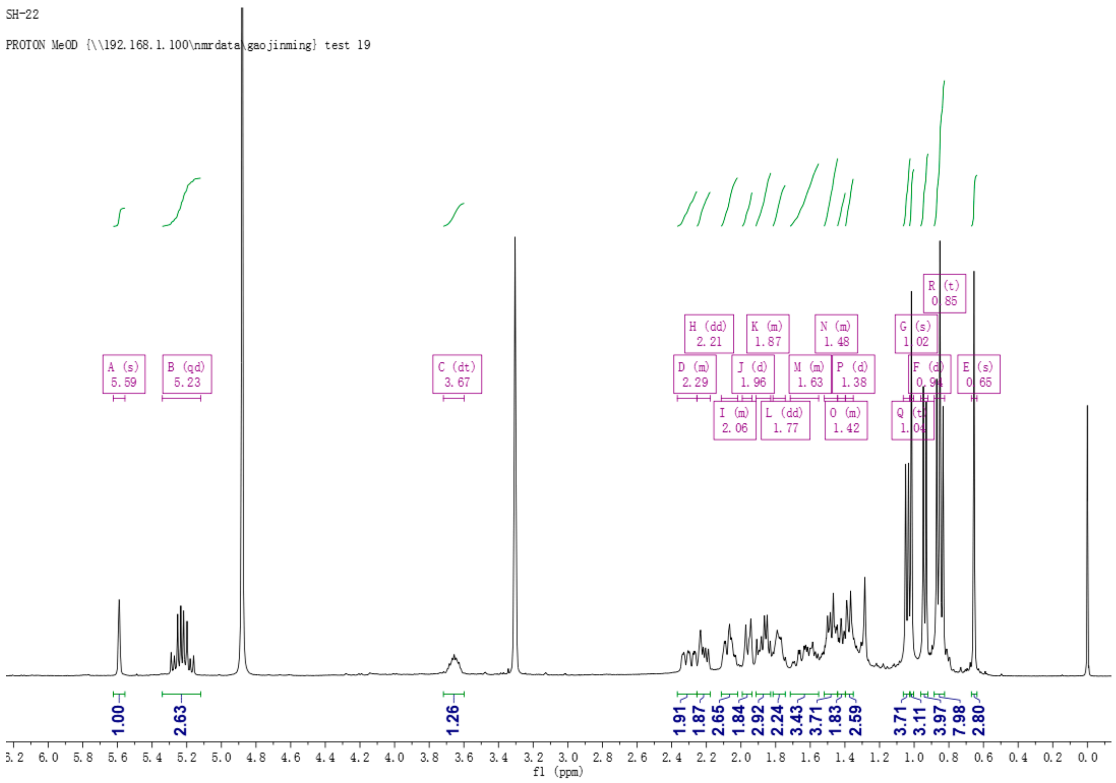

Figure S60. ^{1}H NMR spectrum of compound **18** (400MHz, methanol-d_4)

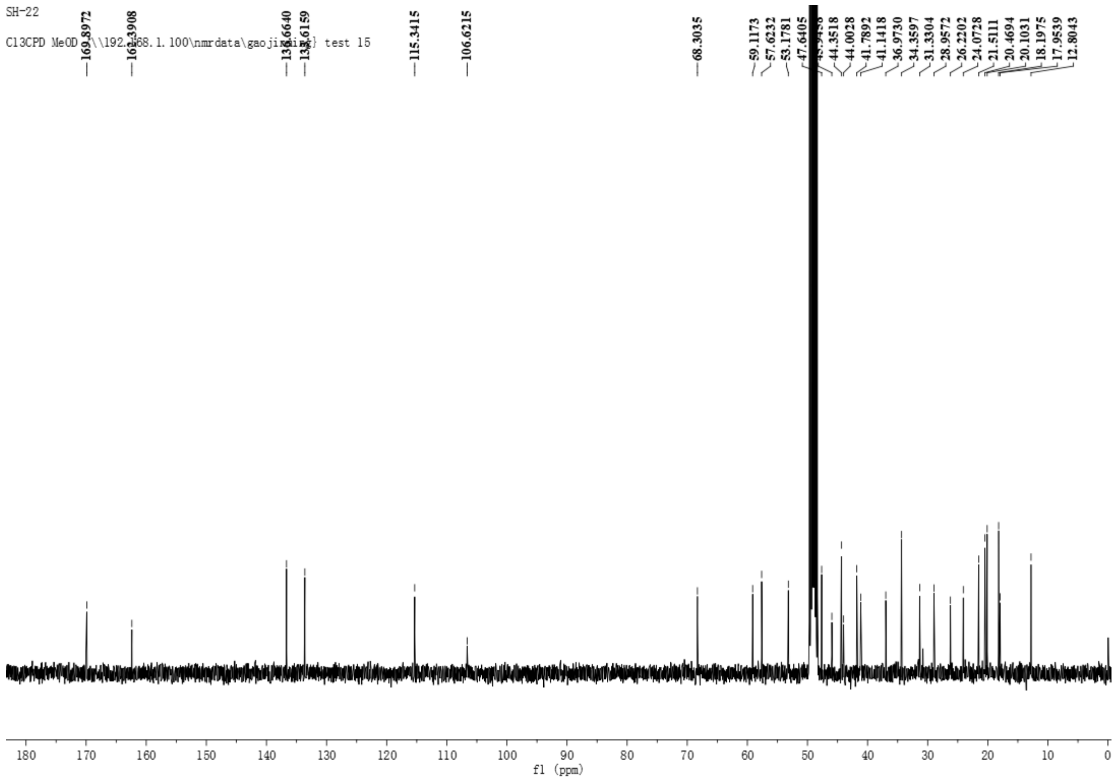

Figure S61. ^{13}C NMR spectrum of compound **18** (100MHz, methanol-d_4)

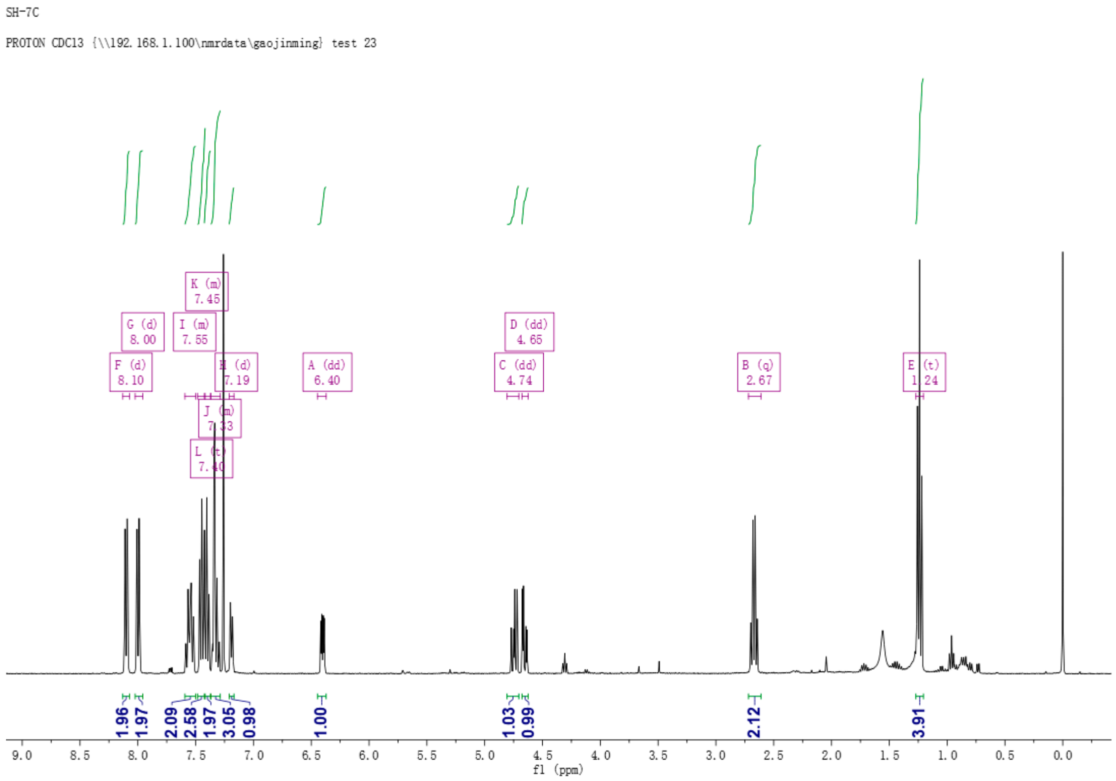

Figure S62. ^{1}H NMR spectrum of compound **19** (400MHz, $CDCl_3$)

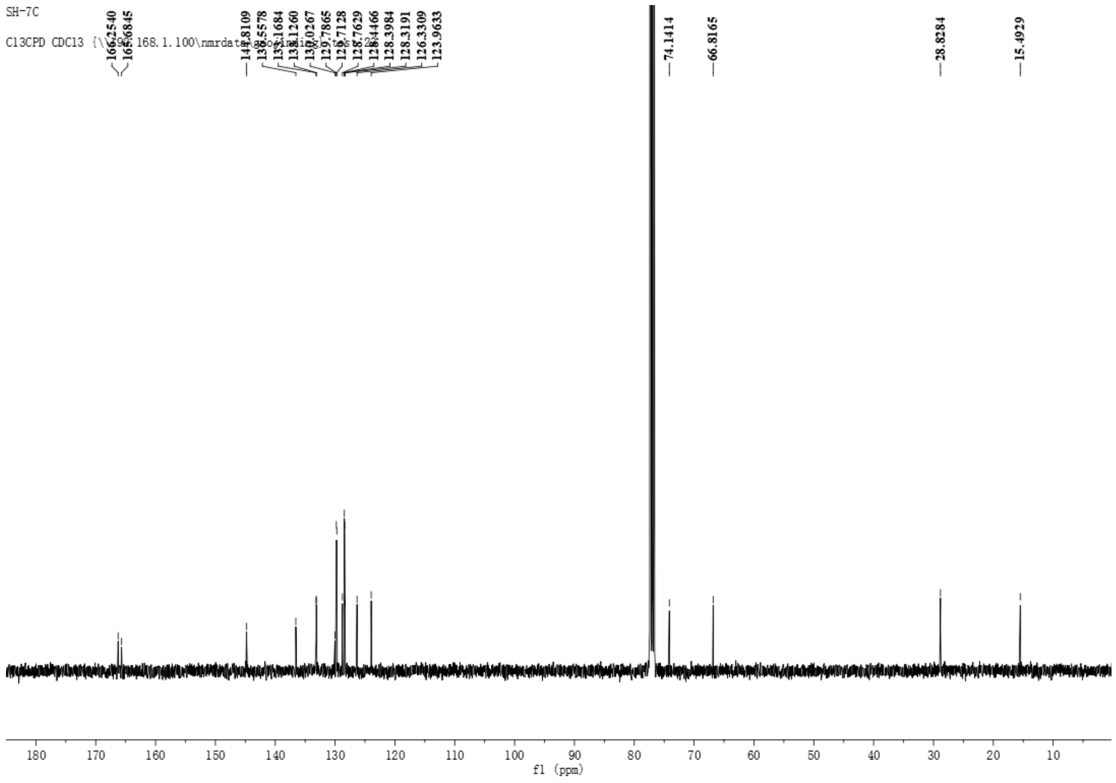

Figure S63. ^{13}C NMR spectrum of compound **19** (100MHz, $CDCl_3$)

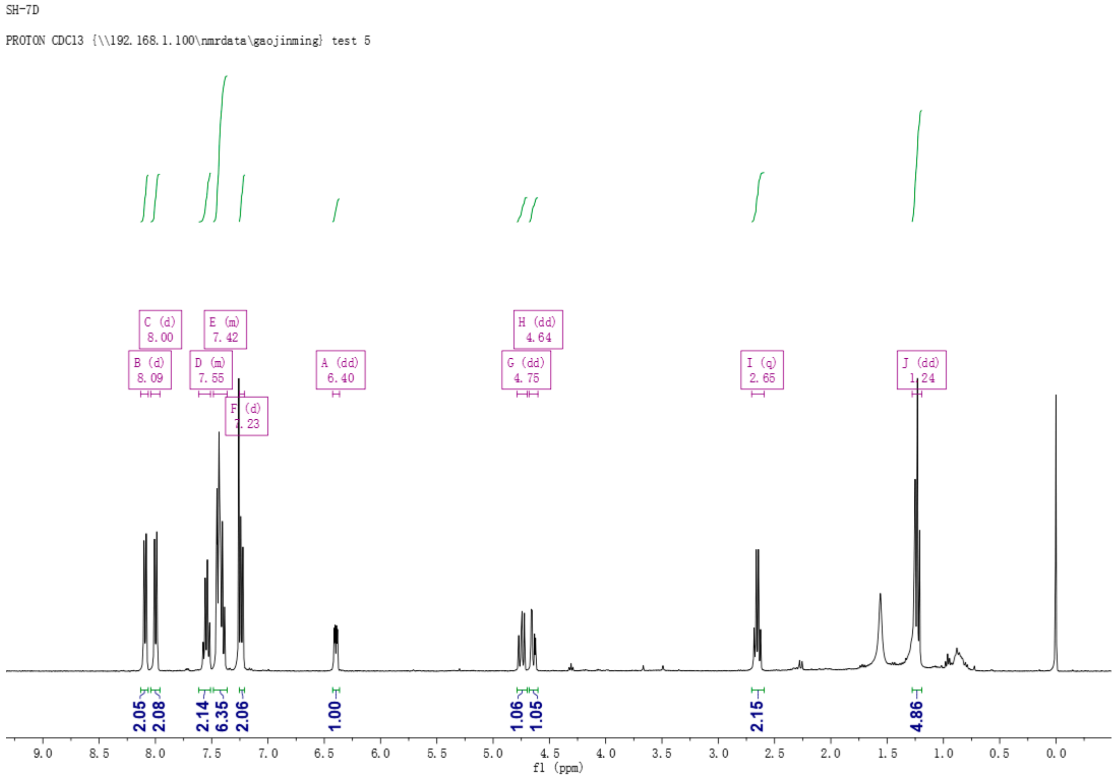

Figure S64. ^{1}H NMR spectrum of compound **20** (400MHz, $CDCl_3$)

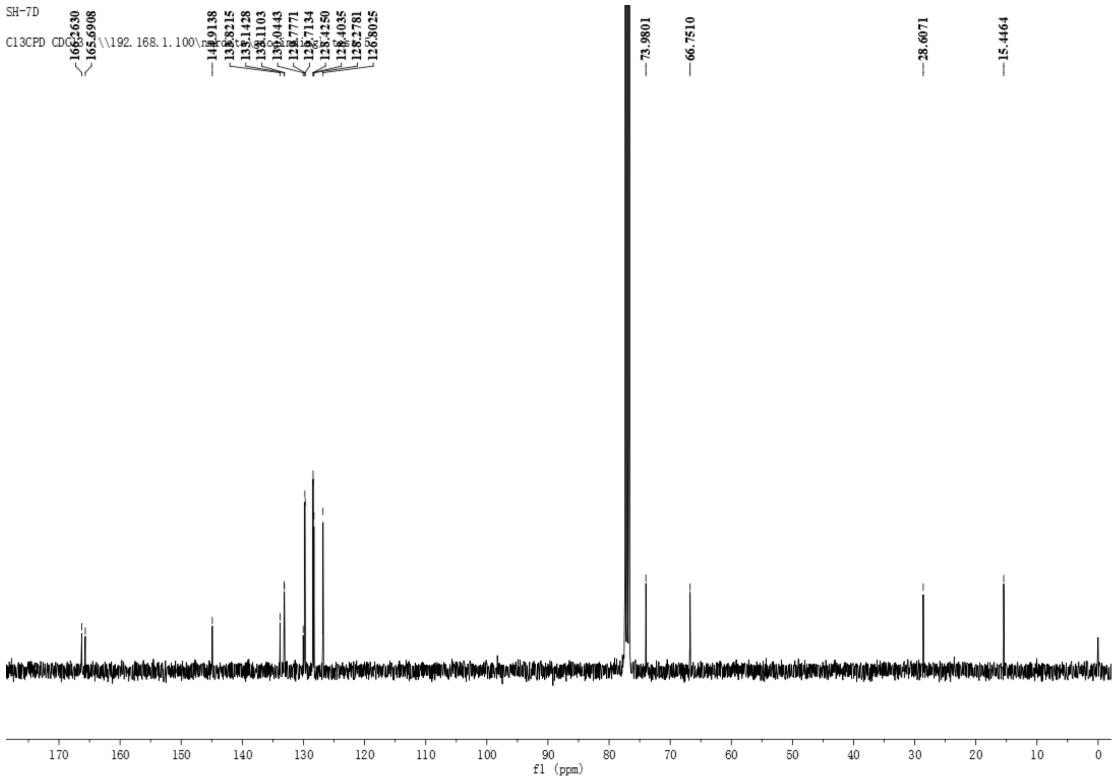

Figure S65. ^{13}C NMR spectrum of compound **20** (100MHz, $CDCl_3$)

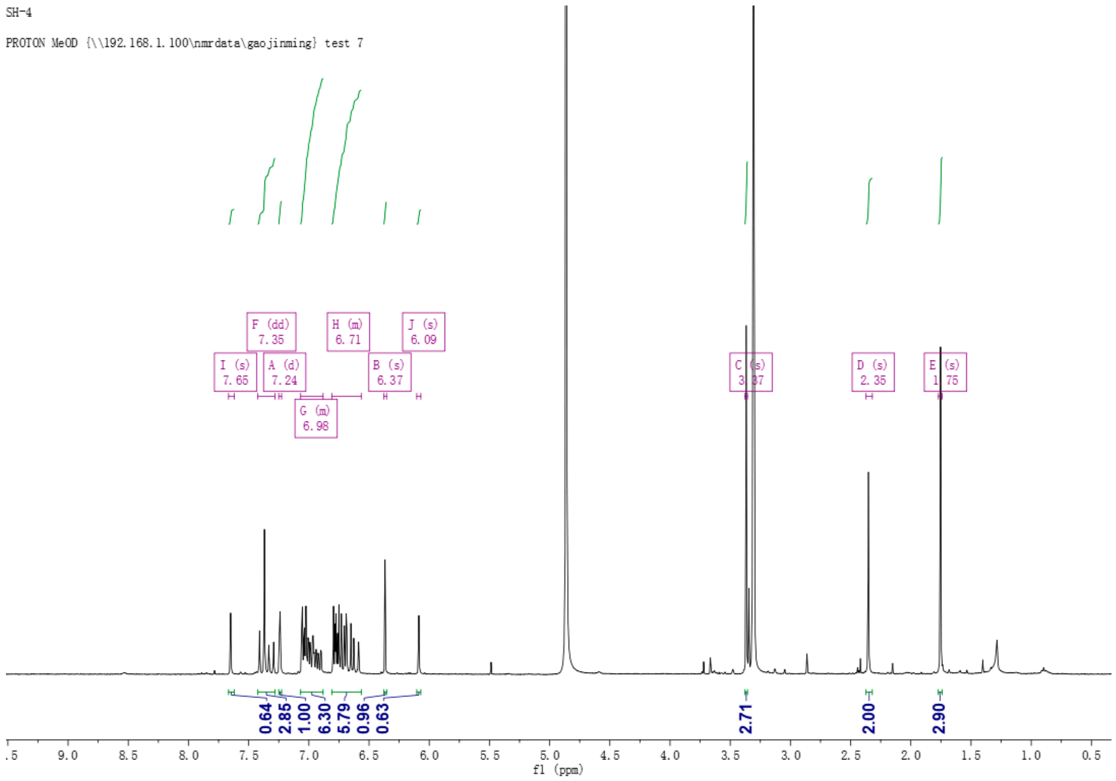

Figure S66. ^{1}H NMR spectrum of compound **21** (400MHz, methanol-d_4)

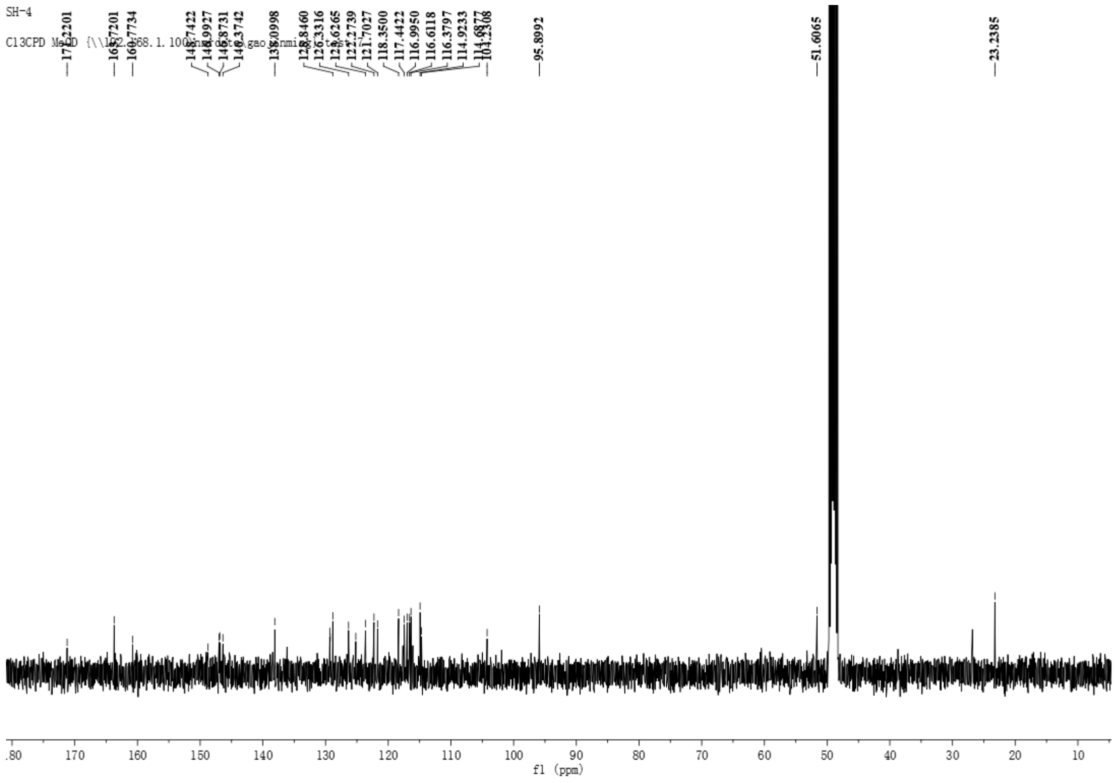

Figure S67. ^{13}C NMR spectrum of compound **21** (100MHz, methanol-d_4)

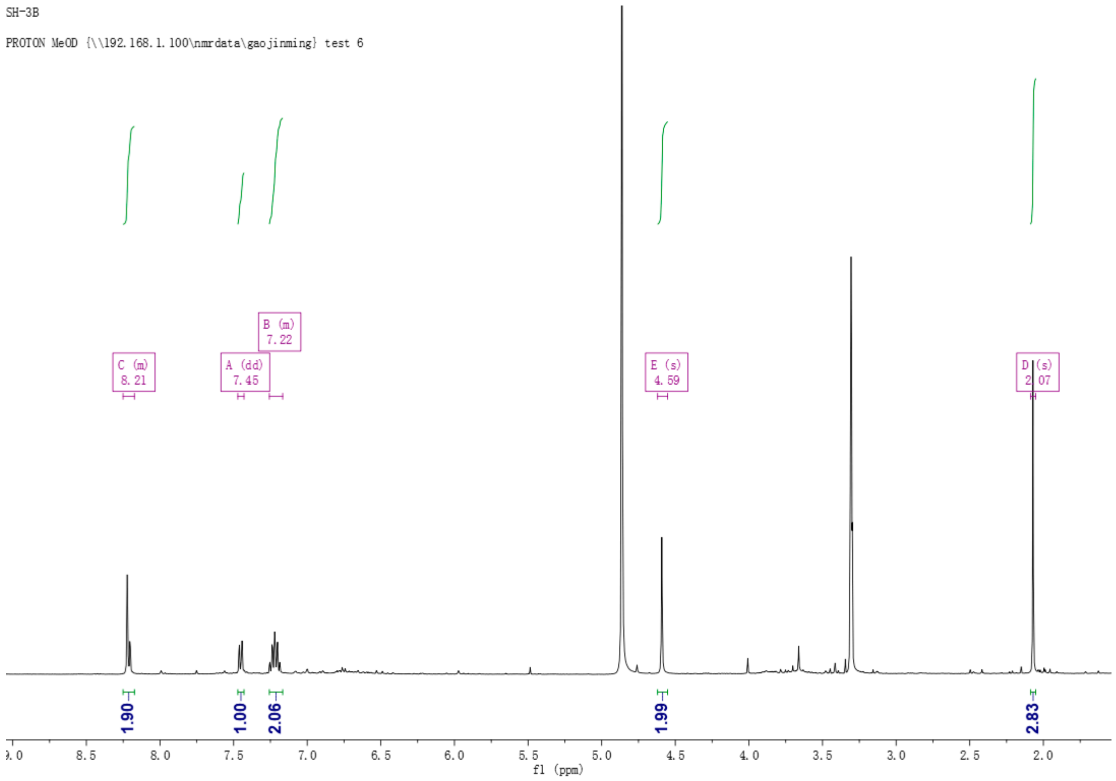

Figure S68. ^{1}H NMR spectrum of compound **24** (400MHz, methanol-d_4)

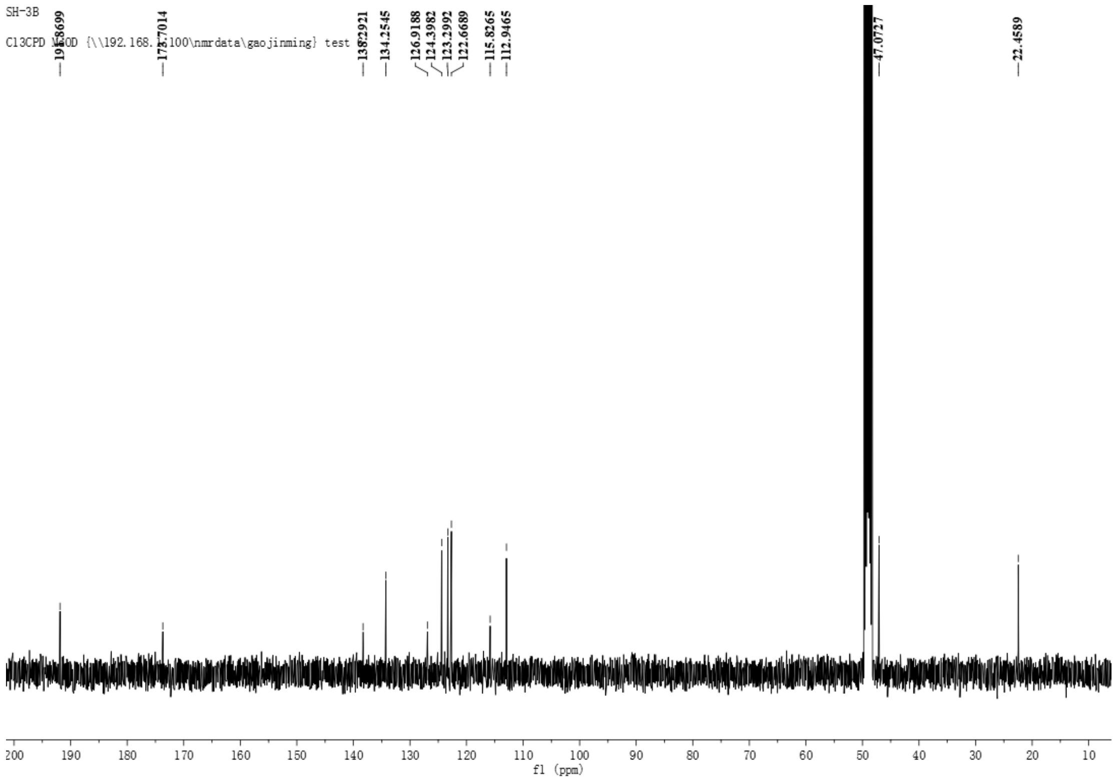

Figure S69. ^{13}C NMR spectrum of compound **24** (100MHz, methanol-d_4)

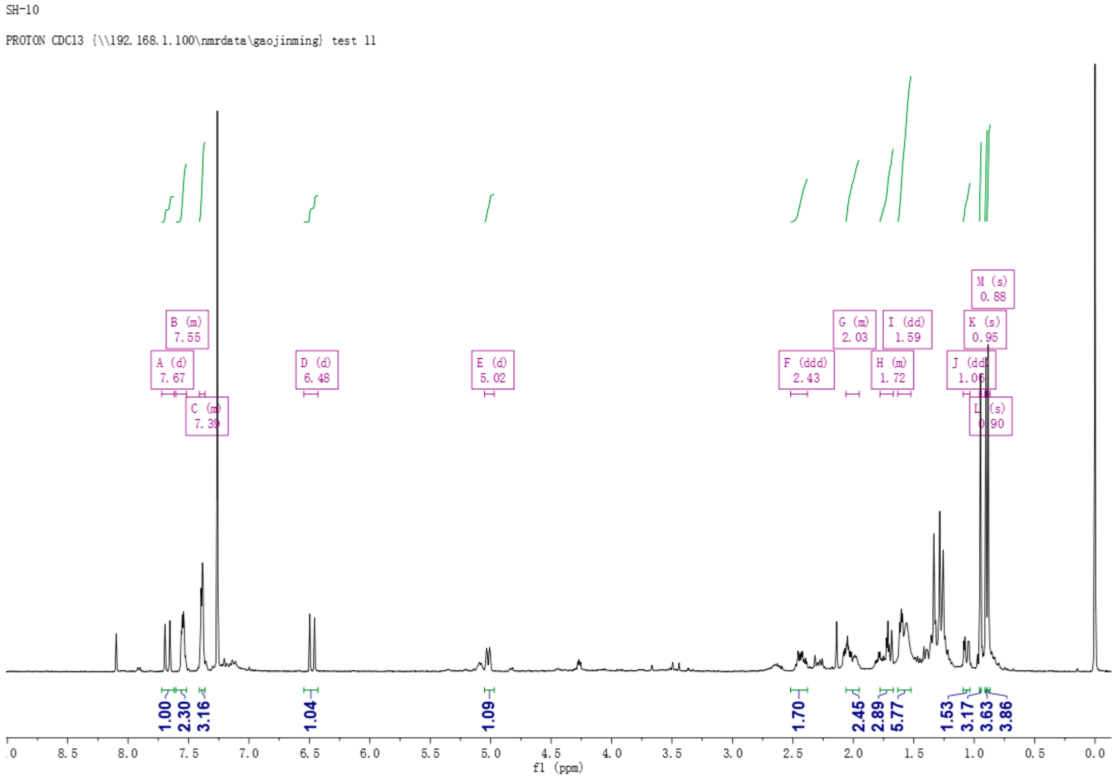

Figure S70. ^{1}H NMR spectrum of compound **25** (400MHz, $CDCl_3$)

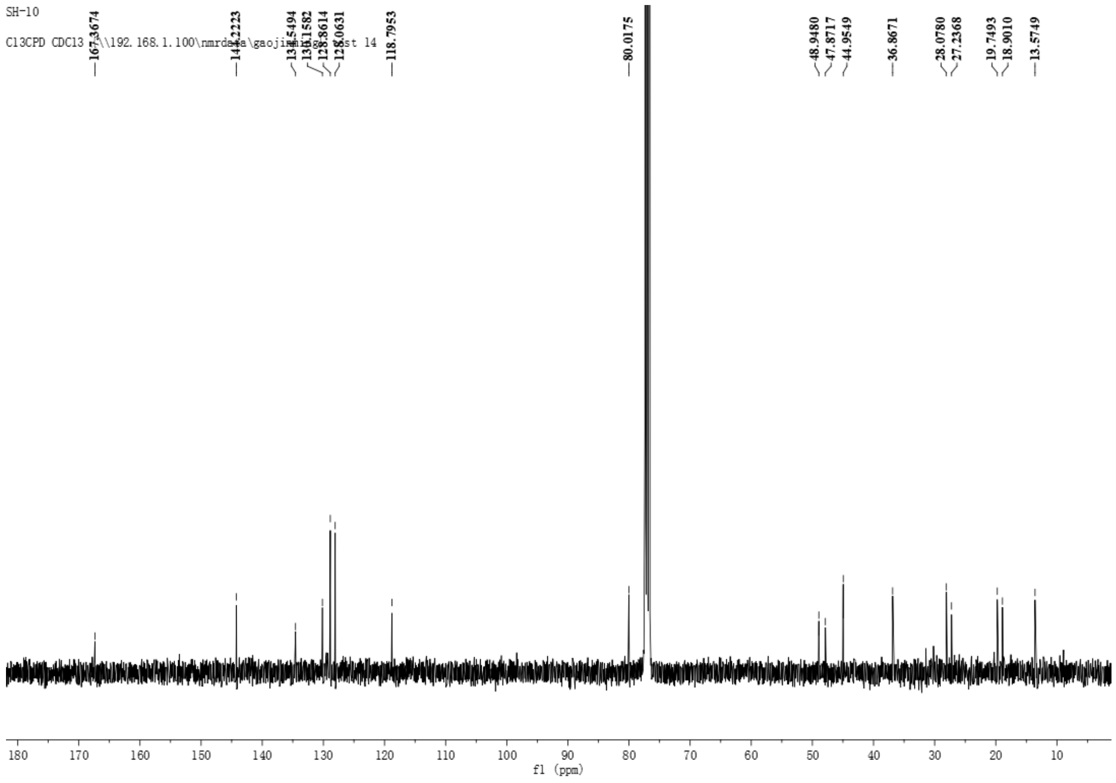

Figure S71. ^{13}C NMR spectrum of compound **25** (100MHz, $CDCl_3$)

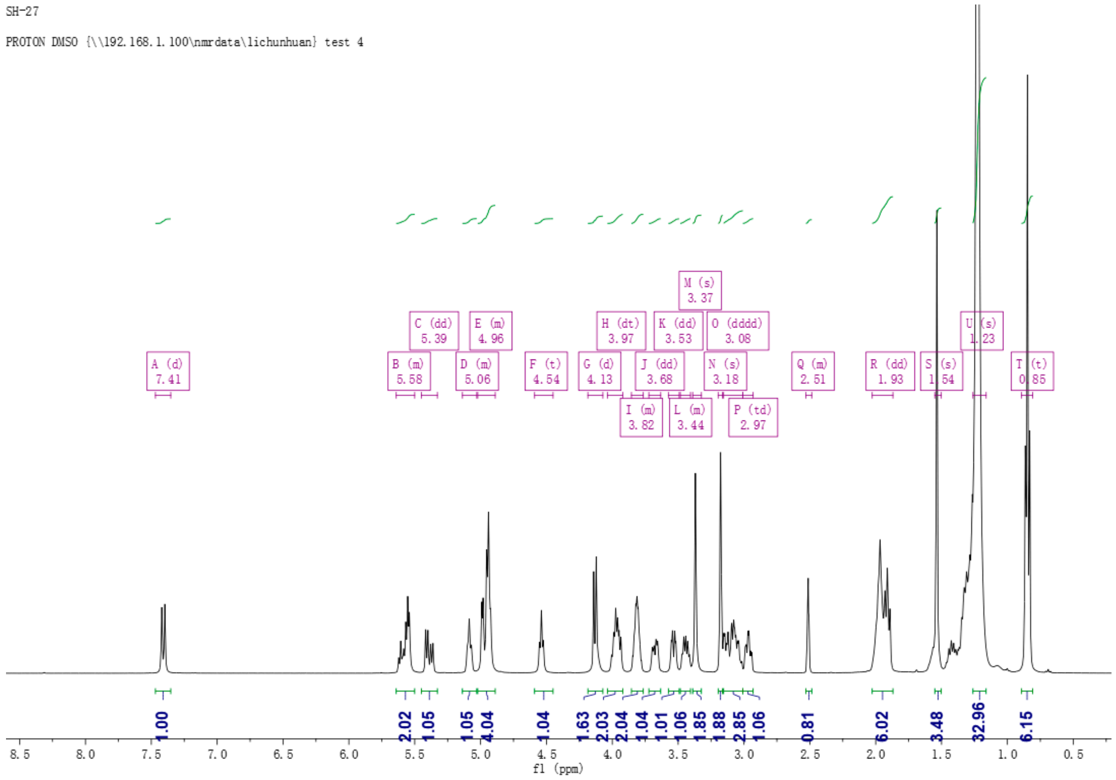

Figure S72. ^{1}H NMR spectrum of compound **26** (400MHz, DMSO-d_6)

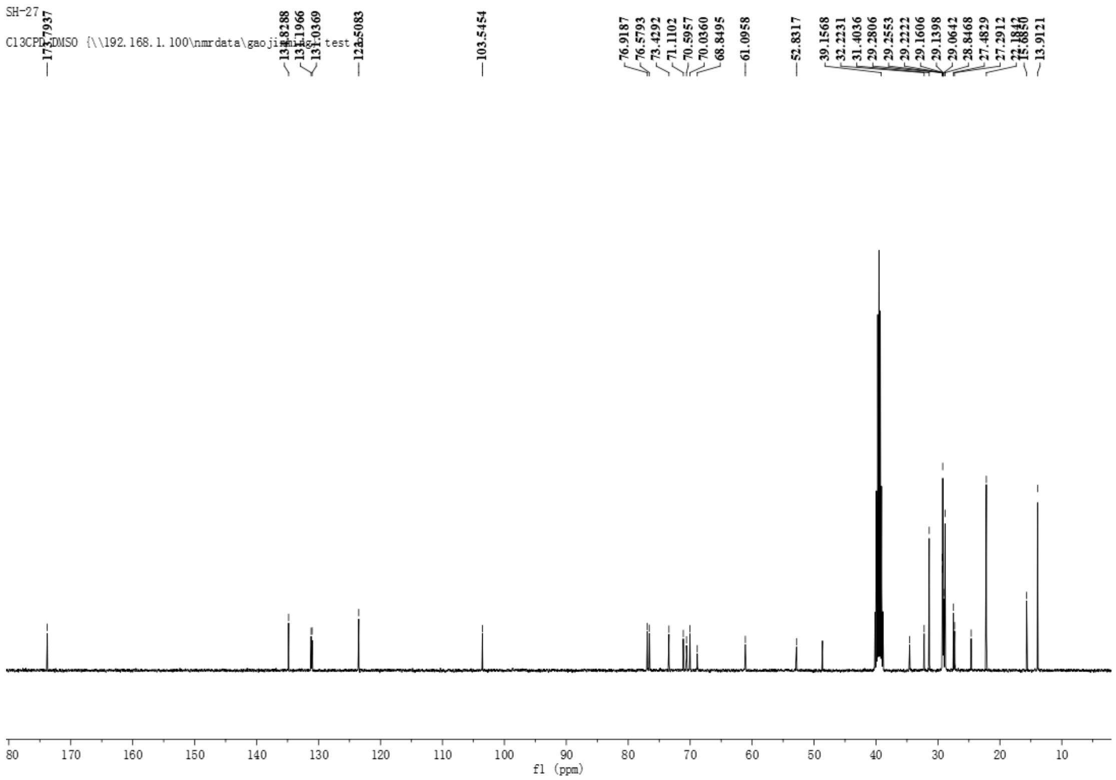

Figure S73. ^{13}C NMR spectrum of compound **26** (100MHz, DMSO-d_6)

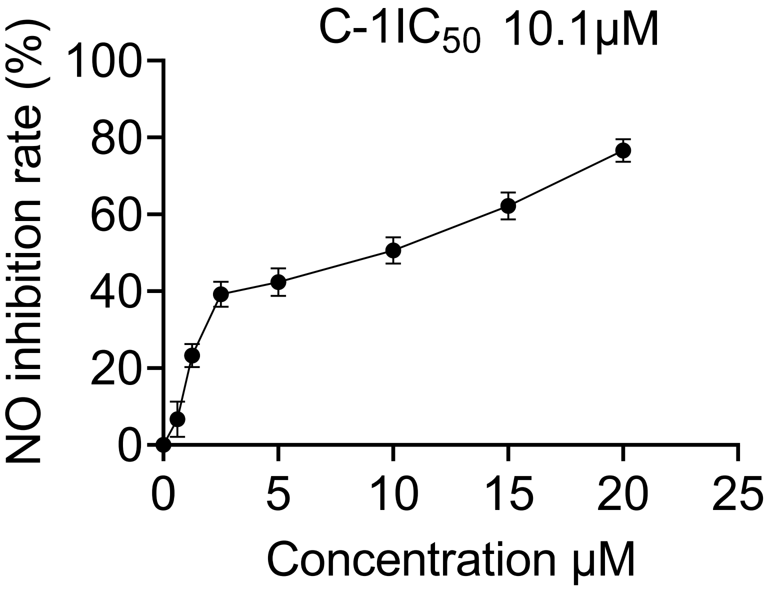

Figure S74. Dose–response curve for the compound **1**.

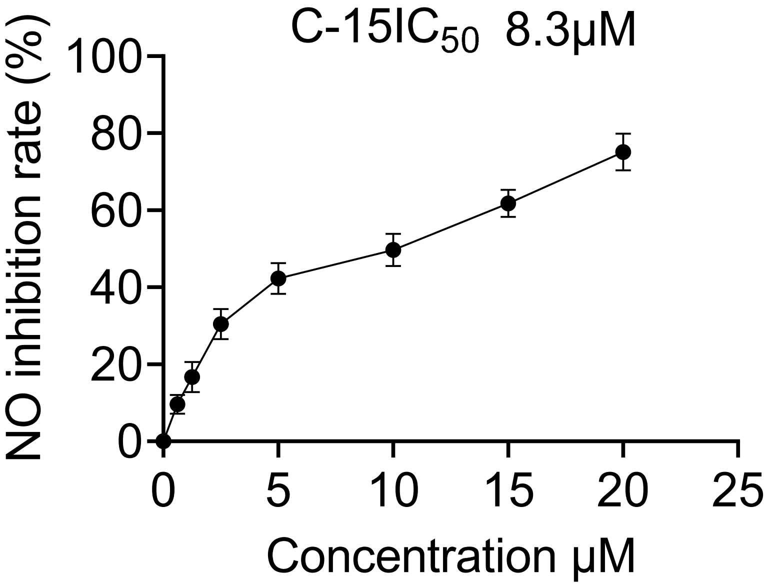

Figure S75. Dose–response curve for the compound **15**.

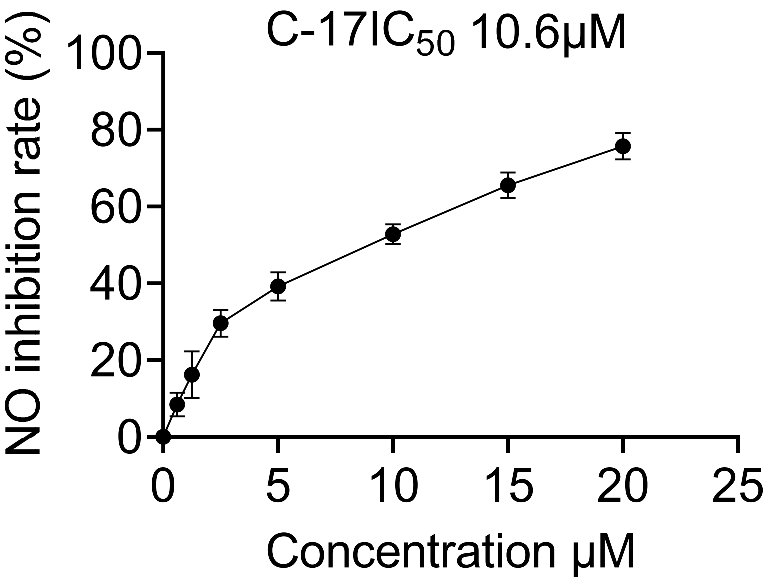

Figure S76. Dose–response curve for the compound **17**.

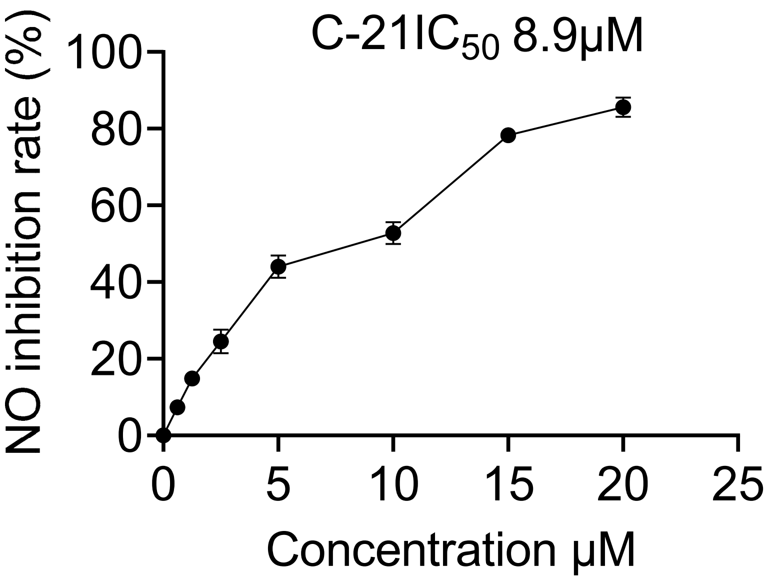

Figure S77. Dose–response curve for the compound **21**.

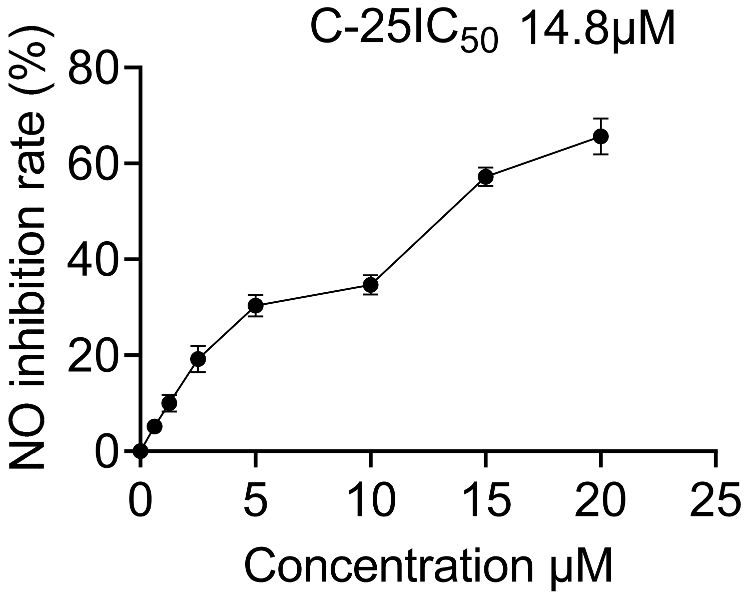

Figure S78. Dose–response curve for the compound **25**.
